# Effectiveness and acceptability of noninvasive brain and nerve stimulation techniques for migraine prophylaxis: a network meta-analysis of randomized controlled trials

**DOI:** 10.1186/s10194-022-01401-3

**Published:** 2022-02-20

**Authors:** Yu-Chen Cheng, Bing-Yan Zeng, Chao-Ming Hung, Kuan-Pin Su, Yi-Cheng Wu, Yu-Kang Tu, Pao-Yen Lin, Brendon Stubbs, Andre F. Carvalho, Chih-Sung Liang, Tien-Yu Chen, Chih-Wei Hsu, Andre R. Brunoni, Mein-Woei Suen, Yow-Ling Shiue, Ping-Tao Tseng, Ming-Kung Wu, Cheng-Ta Li

**Affiliations:** 1 Department of Neurology, Fu Jen Catholic University Hospital, Fu Jen Catholic University, New Taipei City, Taiwan; 2 Department of Neurology, School of Medicine, National Yang-Ming University, Taipei, Taiwan; 3 Division of Community & Rehabilitation Psychiatry, Department of Psychiatry, Taipei Veterans General Hospital, Taipei, Taiwan; 4 Department of Internal Medicine, E-DA Dachang Hospital, Kaohsiung, Taiwan; 5 Division of General Surgery, Department of Surgery, E-Da Cancer Hospital, Kaohsiung, Taiwan; 6 School of Medicine, College of Medicine, I-Shou University, Kaohsiung, Taiwan; 7 Department of Psychiatry & Mind-Body Interface Laboratory (MBI-Lab), China Medical University Hospital, Taichung, Taiwan; 8 College of Medicine, China Medical University, Taichung, Taiwan; 9 An-Nan Hospital, China Medical University, Tainan, Taiwan; 10 Department of Sports Medicine, Landseed International Hospital, Taoyuan, Taiwan; 11 Institute of Epidemiology & Preventive Medicine, College of Public Health, National Taiwan University, Taipei, Taiwan; 12 Department of Dentistry, National Taiwan University Hospital, Taipei, Taiwan; 13 Department of Psychiatry, Kaohsiung Chang Gung Memorial Hospital and Chang Gung University College of Medicine, No.123, Dapi Rd., Niaosong Dist, Kaohsiung City, 833 Taiwan; 14 Institute for Translational Research in Biomedical Sciences, Kaohsiung Chang Gung Memorial Hospital, Kaohsiung, Taiwan; 15 Department of Psychological Medicine, Institute of Psychiatry, Psychology and Neuroscience, King’s College London, London, UK; 16 Physiotherapy Department, South London and Maudsley NHS Foundation Trust, London, UK; 17 Positive Ageing Research Institute (PARI), Faculty of Health, Social Care Medicine and Education, Anglia Ruskin University, Chelmsford, UK; 18 Innovation in Mental and Physical Health and Clinical Treatment (IMPACT) Strategic Research Centre, School of Medicine, Barwon Health, Deakin University, Geelong, VIC Australia; 19 Department of Psychiatry, Beitou branch, Tri-Service General Hospital; School of Medicine, National Defense Medical Center, Taipei, Taiwan; 20 Graduate Institute of Medical Sciences, National Defense Medical Center, Taipei, Taiwan; 21 Department of Psychiatry, Tri-Service General Hospital, School of Medicine, National Defense Medical Center, Taipei, Taiwan; 22 Institute of Brain Science, National Yang Ming Chiao Tung University, Taipei, 112 Taiwan; 23 Service of Interdisciplinary Neuromodulation, National Institute of Biomarkers in Psychiatry, Laboratory of Neurosciences (LIM-27), Departamento e Instituto de Psiquiatria, Faculdade de Medicina da USP, São Paulo, Brazil; 24 Departamento de Ciências Médicas, Faculdade de Medicina da USP, São Paulo, Brazil; 25 Department of Psychology, College of Medical and Health Science, Asia University, Taichung, Taiwan; 26 Gender Equality Education and Research Center, Asia University, Taichung, Taiwan; 27 Department of Medical Research, Asia University Hospital, Asia University, Taichung, Taiwan; 28 Department of Medical Research, China Medical University Hospital, China Medical University, Taichung, Taiwan; 29 Institute of Biomedical Sciences, National Sun Yat-sen University, Kaohsiung, Taiwan; 30 Prospect Clinic for Otorhinolaryngology & Neurology, No. 252, Nanzixin Road, Nanzi District, Kaohsiung City, 81166 Taiwan; 31 Division of Psychiatry, School of Medicine, National Yang Ming Chiao Tung University, No. 201, Sec. 2, Shipai Road, Beitou District, Taipei City, 11267 Taiwan; 32 Institute of Brain Science and Brain Research Center, School of Medicine, National Yang Ming Chiao Tung University, Taipei, Taiwan; 33 Functional Neuroimaging and Brain Stimulation Lab, Taipei Veterans General Hospital, No. 201, Sec. 2, Shipai Road, Beitou District, Taipei City, 11267 Taiwan

**Keywords:** Migraine, Non-invasive brain stimulation, Non-invasive nerve stimulation, Network meta-analysis, Response rate

## Abstract

**Background:**

Current pharmacologic prophylactic strategies for migraine have exhibited limited efficacy, with response rates as low as 40%–50%. In addition to the limited efficacy, the acceptability of those pharmacologic prophylactic strategies were unacceptable. Although noninvasive brain/nerve stimulation strategies may be effective, the evidence has been inconsistent. The aim of this network meta-analysis (NMA) was to compare strategies of noninvasive brain/nerve stimulation for migraine prophylaxis with respect to their effectiveness and acceptability.

**Methods:**

The PubMed, Embase, ScienceDirect, ProQuest, ClinicalTrials.gov, ClinicalKey, Cochrane CENTRAL, Web of Science, and ClinicalTrials.gov databases were systematically searched to date of June 4th, 2021 for randomized controlled trials (RCTs). Patients with diagnosis of migraine, either episodic migraine or chronic migraine, were included. All NMA procedures were conducted under the frequentist model.

**Results:**

Nineteen RCTs were included (*N* = 1493; mean age = 38.2 years; 82.0% women). We determined that the high frequency repetitive transcranial magnetic stimulation (rTMS) over C3 yielded the most decreased monthly migraine days among all the interventions [mean difference = − 8.70 days, 95% confidence intervals (95%CIs): − 14.45 to − 2.95 compared to sham/control groups]. Only alternating frequency (2/100 Hz) transcutaneous occipital nerve stimulation (tONS) over the Oz (RR = 0.36, 95%CIs: 0.16 to 0.82) yielded a significantly lower drop-out rate than the sham/control groups did.

**Conclusions:**

The current study provided a new direction for the design of more methodologically robust and larger RCTs based on the findings of the potentially beneficial effect on migraine prophylaxis in participants with migraine by different noninvasive brain/nerve stimulation, especially the application of rTMS and tONS.

**Trial registration:**

CRD42021252638. The current study had been approval by the Institutional Review Board of the Tri-Service General Hospital, National Defense Medical Center (TSGHIRB No. B-109-29).

**Supplementary Information:**

The online version contains supplementary material available at 10.1186/s10194-022-01401-3.

## Introduction

Migraine, including episodic migraine and chronic migraine, is a highly prevalent neurological disorder worldwide. Reported prevalence rates have ranged from 9.1% to 18.2% [1, 2]. Furthermore, migraine affects women twice as often as men, and the disease is most prevalent at 30–40 years old [2]. Migraine was also estimated to be the second most common source of disability worldwide among neurological disorders [3]. Due to its high prevalence and high disease burden globally, migraine is an important disease that warrants attention.

Pharmacological prophylactic therapies for migraine include beta blockers, calcium channel blockers, angiotensin-II receptor antagonists, anti-epileptics, and antidepressants [4, 5]. However, response rates for these therapies appear to be modest (i.e., approximately 40%–50%) [6]. Moreover, the long-term compliance rates of preventive therapeutic strategies are low at 20%–30% [7]. Therefore, the development of nonpharmacological strategies for migraine prophylaxis is a need in the field that has remained unmet. In particular, noninvasive neuromodulation strategies have been suggested to be effective for migraine prophylaxis [4, 8, 9]. The trigemino-vascular theory (TGVT), based on previous clinical evidences, suggests that the physiopathology of migraine involves three orders neurons, which included the ophthalmic branch of trigeminal nerve, trigeminocervical complex (TCC), and the ventroposteromedial thalamic nucleus [10]. Through this pathway, the neuron firing finally projected to the sensory cortex and resulted in migraine-associated symptoms [11]. In addition, abnormal central pain processing may play a key role in pain modulation and central sensitization among patients with chronic migraine [12]. Therefore, modulation of the sensory trigeminal inputs both at the level of the TCC and the ventroposteromedial thalamic nucleus thereby became a reasonable strategy for the management of migraine [13].

The noninvasive brain and nerve stimulation interventions potentially modulate these pathophysiological mechanisms of migraine through modulation of cortical excitability or acting on peripheral nerves to mitigate aberrations in pain-processing pathways [6]. Noninvasive brain stimulation methods, including transcranial magnetic stimulation (TMS) and transcranial direct current stimulation (tDCS), usually target the pain matrix and related neural networks with the aim of exciting or inhibiting the cerebral cortex to normalize pain-processing transmission. Noninvasive nerve stimulation is targeted at the A-delta and C fibers of the trigeminovascular system because these fibers have been reported to be associated with the pain signal during a migraine episode [14]. Furthermore, unlike the necessity of multiple sessions of TMS in psychiatric disease (such as major depressive disorder) [15–17], a varied number of sessions of TMS has been found to be effective in migraine prophylaxis, either when conducted as single-pulse TMS (sTMS) [18], a single session of repetitive TMS (rTMS) [19], or multiple sessions of rTMS [20]. Electrical stimulation of the somatic branches of the ophthalmic nerve and occipital nerve may activate A-beta fibers, which inhibit second-order nociception in the spinotrigeminal nucleus. Peripheral nerve stimulation has a wider range of targets and may involve noninvasive vagus nerve stimulation (nVNS), percutaneous electrical nerve stimulation (PENS), transcutaneous occipital nerve stimulation (tONS), and supraorbital transcutaneous stimulation (STS) [14, 21, 22].

Network meta-analysis (NMA) has the advantage of allowing for multiple comparisons of efficacy between individual noninvasive brain and nerve stimulations for migraine prophylaxis. Such NMA evidence can thus inform clinical practice [23]. Our present NMA had the primary aim of comparing treatment strategies with respect to their effectiveness (with specific respect to migraine prophylaxis) and their acceptability in patients with migraine.

## Methods

### General guidelines applied in the current study

The current NMA adhered to the latest PRISMA2020 (Preferred Reporting Items for Systematic Reviews and Meta-Analyses) guidelines (eTable 1) [24] and AMSTAR2 (A MeaSurement Tool to Assess systematic Reviews) guideline [25]. The current study had been approval by the Institutional Review Board of the Tri-Service General Hospital, National Defense Medical Center (TSGHIRB No. B-109-29) and been registered on PROSPERO (PROSPERO registration: CRD42021252638).

### Search strategy and selection criteria

We conducted a systematic search for publications using the following search terms: (deep transcranial magnetic stimulation OR dTMS OR repetitive transcranial magnetic stimulation OR rTMS OR TMS OR non-invasive brain stimulation OR theta burst stimulation OR transcranial direct current stimulation OR TBS OR tDCS OR vagus nerve stimulation OR vagal nerve stimulation OR tVNS OR nVNS OR VNS OR static magnetic field stimulation OR SMS OR tSMS) AND (migraine OR migrain* OR migraine disorder) AND (random OR randomized OR randomised). We searched the databases of PubMed, Embase, ScienceDirect, ProQuest, ClinicalTrials.gov, ClinicalKey, Cochrane CENTRAL, and Web of Science. The grey literature had been searched on ClinicalTrials.gov. The final date of the literature search was done on June 4th, 2021 (eTable 2). No language restriction was imposed. In addition to these database searches, we manually searched for potentially eligible articles cited in review articles and pairwise meta-analyses [26–39].

### Inclusion criteria and exclusion criteria

The PICO of the current study included: (1) Patient: migraine patients with either episodic migraine or chronic migraine; (2) Intervention: non-invasive brain/nerve stimulation; (3) Comparator: sham-control or active control; and (4) Outcome: changes of migraine frequency or response rate (which was defined as below). We only included RCTs with human participants that investigated the efficacy of noninvasive brain and nerve stimulation in migraine prophylaxis. The intervention arms of interest were set to be noninvasive brain and nerve stimulation as applied to patients with migraine; such migraine could be episodic migraine, chronic migraine, or mixed episodic/chronic migraine.

Studies were excluded if they (1) were not clinical trials, (2) were not RCTs, (3) did not report the target outcomes of interest, or (4) were not specific to patients with migraine. In situation that the same set of data had been used by multiple studies, we only included the most informative study with the largest sample.

### Data extraction

Two authors independently screened the studies for inclusion, extracted the relevant data from the manuscripts, and assessed the risk of bias in the included studies. Where these authors disagreed, the corresponding author adjudicated the disagreement. If the manuscripts lacked relevant data, we contacted the corresponding authors or co-authors to obtain the originally used data. We followed the research process of previous network meta-analyses [40–46].

### Outcomes

Because the aim of therapy for migraine is not complete remission but the reduction of migraine frequency [19, 47], we chose the changes in monthly migraine days and response rate as the primary outcomes. Specifically, about the data extraction of outcome “changes in monthly migraine days”, because not all the migraine patients could clearly classify the current headache episode into migraine or other-type of headache, the RCTs applying headache diary might have some methodological limitation. Therefore, if there was both “changes in monthly migraine days” and “changes in monthly headache days” in one RCT, we choose to use “changes in monthly migraine days” first. If there is no “changes in monthly migraine days” available in one RCT, we will choose to extract “changes in monthly headache days”. A successful response rate was defined as a ≥ 50% reduction in migraine frequency or pain-free rate, depending on a given study’s definition. Our secondary outcome was posttreatment migraine pain severity and changes in frequency of acute rescue medication use. The acceptability was set as the drop-out rate, which was defined as a participant leaving the study before the end of the trial for any reason.

### Cochrane risk of bias tool

Two authors independently evaluated the risk of bias (interrater reliability = 0.87) for each domain, per the Cochrane risk of bias tool [48]. Studies were then further classified by risk of bias.

### Statistical analysis

We performed the NMA on STATA version 16.0 (StataCorp LLC, College Station, TX, USA). For continuous data, we estimated the summary standardized mean difference (SMD) in situation of different kinds of rating scales and the mean difference (MD) in situation of uniform units in individual outcome. For categorical data, we estimated the summary rate ratio (RR). The SMD, MD, and RR were estimated with their corresponding 95% confidence interval (95%CIs). For categorical data, we applied a 0.5 zero-cell correction in the meta-analysis procedure. However, for studies with 0 in both the intervention and control arms, we did not apply such correction because bias might be increased by doing so [49, 50]. We used the frequentist model of NMA to compare the effect sizes (ES) between studies with the same intervention. All comparisons were made using a two-tailed test, where *p <* 0.05 indicated statistical significance. Heterogeneity among the included studies was evaluated using the tau statistic, which is the estimated standard deviation of the effect across the included studies.

As for the analytical procedure of this study, we employed a mixed comparison with generalized linear mixed model to analyze the direct and indirect comparisons among the NMA [51]. Specifically, indirect comparisons were made by assuming transitivity—that is, we assumed that the hitherto unknown difference between treatments A and B could be determined from known differences between A and C and between B and C, where C is a third treatment. Subsequently, to compare between the multiple treatment arms, we combined the direct and indirect evidence obtained from the included studies [52]. Direct evidence for the difference between any two treatment arms was obtained from at least one of the studies comparing both treatments. Indirect evidence for the difference in ES between two treatment arms was obtained through the aforementioned method of assuming transitivity. We used the *mvmeta* command in STATA [53]. We used the method of restricted maximum likelihood to evaluate between-study variance [54].

To increase the clinical applicability of our findings, we calculated the relative ranking probabilities between the treatment effects of all treatments on the target outcomes. Specifically, we used the surface under the cumulative ranking curve (SUCRA), which is the percentage of the mean rank of each intervention relative to the worst imaginary intervention without uncertainty [55]. Low SUCRA values corresponded to higher ranks of migraine prophylaxis.

We evaluated inconsistencies between the direct and indirect evidence in the network using (1) the loop-specific approach and (2) determinations of local inconsistency through the node-splitting method. We then used the design-by-treatment model to evaluate global inconsistency (i.e., across the entire NMA) [56]. The quality of evidence was evaluated with the GRADE tools. To be specific, we evaluated the GRADE ratings according to the rationale of the articles published in the BMJ [57] and the Lancet [58]. Finally, per the rationale of a previous NMA study [15], we assessed the effectiveness of the different sham interventions to additionally justify our assumption of transitivity. Specifically, we computed the sham therapy effect for tDCS sham therapy, nVNS sham therapy, rTMS sham therapy, sTMS sham therapy, STS sham therapy, tONS sham therapy, and PENS sham therapy by the traditional pairwise meta-analysis using Comprehensive Meta-Analysis (version 3; Biostat, Englewood, NJ, USA) [59]. To maintain the quality of pairwise meta-analysis, we only conducted the pairwise meta-analysis in situation of at least two studies included. In addition, we arrange further subgroup analysis to justify our assumption hypothesis. To be specific, we arrange subgroup analysis based on participants with episodic migraine (i.e. migraine days < 15 days/month) or chronic migraine (i.e. migraine days > 15 days/month).

## Results

**Fig. 1:**
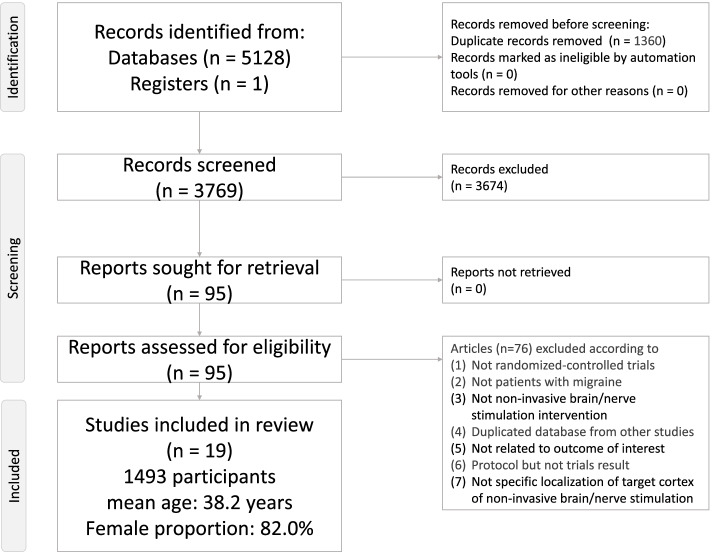
Flowchart of the network meta-analysis procedure. Flowchart illustrating the procedure of the present network meta-analysis

**Fig. 2:**
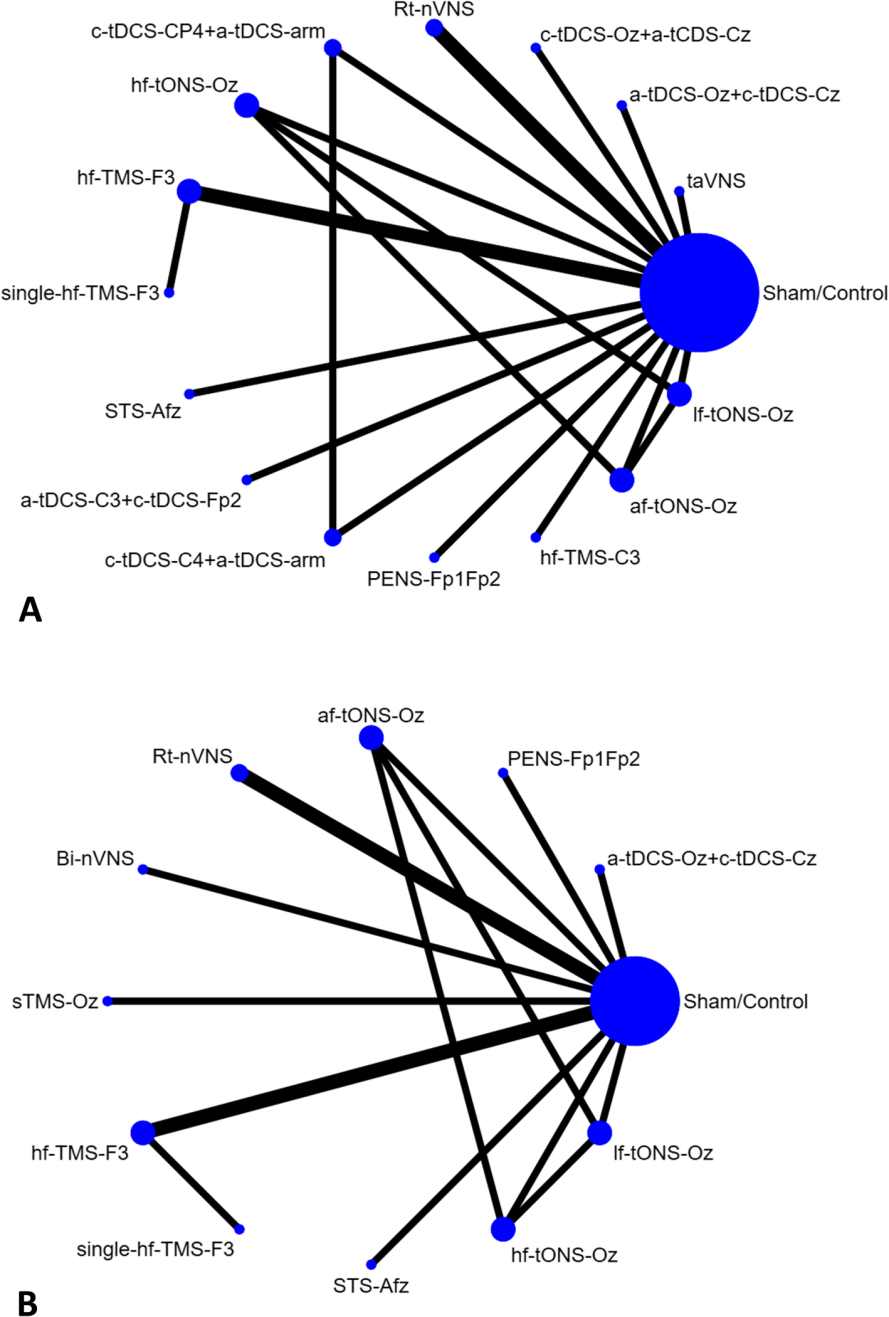
Network structure of primary outcome: (**A**) changes in monthly migraine days and (**B**) response rate. AB Overall network structure of the current network meta-analysis for the primary outcome of response rate. The lines between nodes represent direct comparisons in various trials, and the size of each circle is proportional to the number of participants receiving each specific treatment. The thickness of the lines is proportional to the number of trials connected to the network

After the initial screening procedure, 95 articles were considered for a full-text review (Fig. 1: Flowchart of the network meta-analysis procedure). However, three articles had been excluded because all the recruited patients among these articles were comorbid with medication overuse, which was inconsistent with the other studies and violate the similarity hypothesis [60–62]. Overall 76 articles were excluded for various reasons (Fig. 1 and eTable 3), leaving 19 articles for final inclusion in the network meta-analysis [18–20, 22, 63–77] (eTable 4). Among these 19 articles, the Lipton, R.B. (2010) allowed the patients to bring the machine back home to keep the TMS treatment [18]. According to the description of this article, the blindness (i.e. masking) was similar with the other included RCTs. The post-study survey to assess errors and overall user-friendliness of the TMS device revealed that patients rarely experienced errors and rated the TMS device to have 8 on a 10-point scale for overall user-friendliness [18]. Therefore, we decided to include this study due to the fair quality control of the home-based TMS in this study in comparison with the other hospital-based study. The overall network structure of the treatment arms is illustrated in Fig. 2A-B (Network structure of primary outcome: (A) changes in monthly migraine days and (B) response rate). Because of the significantly different baseline migraine severities between the study groups in Conforto (2014) [66], we did not use the outcome result from this study but did use their data for drop-out rate.

### Characteristics of included studies

The 19 RCTs, which were published between 2004 and 2021, had 1493 participants in total. The mean age was 38.2 years (range of mean age in the RCTs: 30.4 to 51.7 years), and 82.0% of participants were women (range of proportion of female participants for each RCT: 53.8% to 100.0%). The mean follow-up duration was 11.4 weeks (range of follow-up duration in the RCTs: 4 to 62 weeks). All RCTs did not prohibit the concurrent use of antimigraine medication.

### Primary outcome: the changes in monthly migraine days

**Fig. 3:**
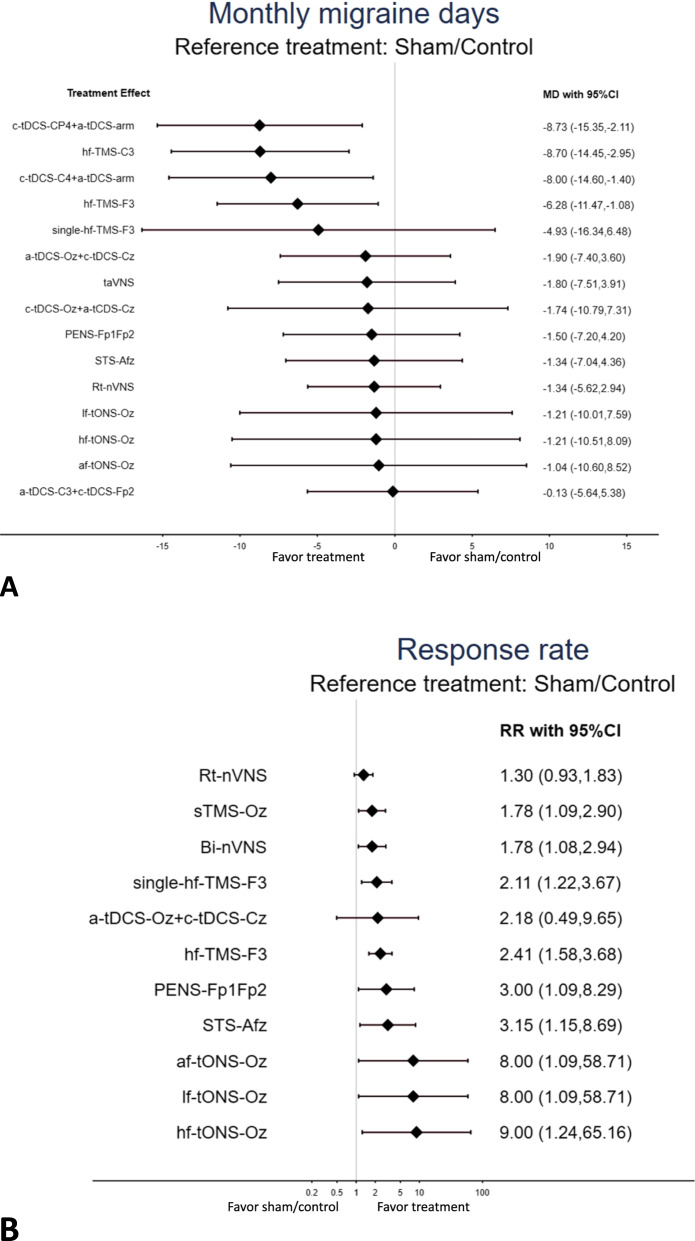
Forest plot of primary outcome: (**A**) changes in monthly migraine days and (**B**) response rate. When the effect size was (**A**) < 0 (presented as the mean difference) or (B) > 1 (presented as the rate ratio), the specified treatment yielded (A) a better improvement in monthly migraine days or (**B**) a higher response rate than its corresponding sham/control group did

The main result revealed that cathode tDCS over CP4 + anode at left upper arm (c-tDCS-CP4 + a-tDCS-arm; MD = -8.73 days, 95%CIs: − 15.35 to − 2.11), high frequency rTMS over C3 (hf-TMS-C3; MD = -8.70 days, 95%CIs: − 14.45 to − 2.95), cathode tDCS over C4 + anode at left upper arm (c-tDCS-C4 + a-tDCS-arm; MD = -8.00 days, 95%CIs: − 14.60 to − 1.40), and high frequency rTMS over F3 (hf-TMS-F3; MD = -6.28, 95%CIs: − 11.47 to − 1.08) were significantly associated with better reduced monthly migraine days than the sham/control groups did (Table 1, Fig. 2A, and Fig. 3A (Forest plot of primary outcome: changes in monthly migraine days)). According to the SUCRA results, hf-TMS-C3 yielded the most decreased monthly migraine days among all the interventions (eTable 5A).

**Table 1:** League table of the outcome of changes in monthly migraine days

hf-TMS-C3															*-8.70 (-10.41,-6.99)
0.03 (-8.74,8.80)	c-tDCS-CP4+a-tDCS-arm	-0.73 (-1.98,0.52)													*-8.73 (-12.44,-5.02)
-0.70 (-9.45,8.05)	-0.73 (-6.35,4.89)	c-tDCS-C4+a-tDCS-arm													*-8.00 (-11.66,-4.34)
-2.42 (-10.17,5.33)	-2.45 (-10.87,5.97)	-1.72 (-10.12,6.67)	hf-TMS-F3	-1.35 (-9.90,7.20)											-6.81 (-13.86,0.24)
-3.77 (-16.55,9.01)	-3.80 (-16.99,9.39)	-3.07 (-16.25,10.11)	-1.35 (-11.50,8.81)	single-hf-TMS-F3											
-6.80 (-14.75,1.15)	-6.83 (-15.44,1.78)	-6.10 (-14.69,2.49)	-4.38 (-11.94,3.19)	-3.03 (-15.70,9.64)	a-tDCS-Oz+c-tDCS-Cz										*-1.90 (-2.27,-1.53)
-6.96 (-17.68,3.76)	-6.99 (-18.21,4.22)	-6.26 (-17.46,4.94)	-4.54 (-14.97,5.90)	-3.19 (-17.76,11.37)	-0.16 (-10.75,10.43)	c-tDCS-Oz+a-tCDS-Cz									-1.74 (-8.94,5.46)
-6.90 (-15.00,1.20)	-6.93 (-15.67,1.81)	-6.20 (-14.92,2.52)	-4.48 (-12.20,3.24)	-3.13 (-15.89,9.63)	-0.10 (-8.03,7.83)	0.06 (-10.64,10.76)	taVNS								*-1.80 (-3.38,-0.22)
-7.20 (-15.29,0.89)	-7.23 (-15.97,1.51)	-6.50 (-15.22,2.22)	-4.78 (-12.49,2.94)	-3.43 (-16.19,9.32)	-0.40 (-8.32,7.52)	-0.24 (-10.93,10.46)	-0.30 (-8.37,7.77)	PENS-Fp1Fp2							-1.50 (-3.05,0.05)
-7.49 (-18.00,3.02)	-7.52 (-18.53,3.50)	-6.79 (-17.78,4.21)	-5.06 (-15.28,5.16)	-3.72 (-18.13,10.69)	-0.69 (-11.06,9.69)	-0.52 (-13.15,12.10)	-0.59 (-11.08,9.90)	-0.29 (-10.77,10.20)	lf-tONS-Oz			0.00 (-7.72,7.72)	-0.17 (-8.20,7.86)		-1.21 (-8.10,5.67)
-7.36 (-15.45,0.73)	-7.39 (-16.13,1.35)	-6.66 (-15.38,2.06)	-4.94 (-12.65,2.78)	-3.59 (-16.35,9.16)	-0.56 (-8.48,7.36)	-0.40 (-11.09,10.30)	-0.46 (-8.53,7.61)	-0.16 (-8.22,7.90)	0.13 (-10.36,10.61)	STS-Afz					-1.34 (-2.89,0.21)
*-7.36 (-14.53,-0.20)	-7.39 (-15.28,0.49)	-6.66 (-14.53,1.20)	-4.94 (-11.52,1.64)	-3.59 (-15.70,8.51)	-0.56 (-7.53,6.41)	-0.40 (-10.41,9.61)	-0.46 (-7.60,6.67)	-0.16 (-7.29,6.97)	0.12 (-9.66,9.91)	-0.00 (-7.13,7.13)	Rt-nVNS				-0.71 (-2.06,0.64)
-7.49 (-18.42,3.45)	-7.52 (-18.93,3.90)	-6.79 (-18.19,4.62)	-5.06 (-15.72,5.59)	-3.72 (-18.44,11.00)	-0.69 (-11.49,10.12)	-0.52 (-13.50,12.45)	-0.59 (-11.50,10.33)	-0.29 (-11.19,10.62)	0.00 (-9.47,9.47)	-0.13 (-11.03,10.78)	-0.12 (-10.36,10.12)	hf-tONS-Oz	-0.17 (-8.75,8.40)		-1.21 (-8.72,6.30)
-7.66 (-18.81,3.49)	-7.69 (-19.32,3.94)	-6.96 (-18.57,4.65)	-5.24 (-16.12,5.64)	-3.89 (-18.77,10.99)	-0.86 (-11.88,10.17)	-0.70 (-13.86,12.46)	-0.76 (-11.89,10.37)	-0.46 (-11.59,10.67)	-0.17 (-9.90,9.55)	-0.30 (-11.43,10.83)	-0.30 (-10.77,10.17)	-0.17 (-10.35,10.00)	af-tONS-Oz		-1.04 (-8.87,6.79)
*-8.57 (-16.53,-0.61)	-8.60 (-17.21,0.01)	-7.87 (-16.46,0.72)	-6.15 (-13.72,1.43)	-4.80 (-17.47,7.87)	-1.77 (-9.55,6.01)	-1.61 (-12.20,8.99)	-1.67 (-9.60,6.26)	-1.37 (-9.30,6.56)	-1.08 (-11.46,9.30)	-1.21 (-9.14,6.72)	-1.21 (-8.18,5.77)	-1.08 (-11.89,9.73)	-0.91 (-11.94,10.12)	a-tDCS-C3+c-tDCS-Fp2	-0.13 (-0.63,0.37)
*-8.70 (-14.45,-2.95)	*-8.73 (-15.35,-2.11)	*-8.00 (-14.60,-1.40)	*-6.28 (-11.47,-1.08)	-4.93 (-16.34,6.48)	-1.90 (-7.40,3.60)	-1.74 (-10.79,7.31)	-1.80 (-7.51,3.91)	-1.50 (-7.20,4.20)	-1.21 (-10.01,7.59)	-1.34 (-7.04,4.36)	-1.34 (-5.62,2.94)	-1.21 (-10.51,8.09)	-1.04 (-10.60,8.52)	-0.13 (-5.64,5.38)	Sham/Control

Pairwise (upper-right portion) and network (lower-left portion) meta-analysis results are presented as estimate effect sizes for the outcome of improvement of monthly migraine days. Interventions are reported in order of mean ranking of monthly migraine days improvement, and outcomes are expressed as mean difference (MD) (95% confidence intervals). For the pairwise meta-analyses, MD of less than 0 indicate that the treatment specified in the row got more improvement than that specified in the column. For the network meta-analysis (NMA), MD of less than 0 indicate that the treatment specified in the column got more improvement than that specified in the row. Bold results marked with * indicate statistical significance

The assumption of transitivity was verified using the interaction test [15, 59]. There was no significantly different sham therapy effect between nVNS sham therapy, rTMS sham therapy and tDCS sham therapy (*p* = 0.151; eFigure 1A). However, there was significantly nVNS sham therapy effect detected (SMD = -0.330, 95%CIs: − 0.619 to − 0.040, *p* = 0.025).

The subgroup analysis based on chronic migraine or episodic migraine revealed similar findings. In the chronic migraine subgroup, hf-TMS-F3 (MD = -10.97, 95%CIs: − 17.09 to − 4.84) and hf-TMS-C3 (MD = -8.70 days, 95%CIs: − 10.71 to − 6.69) were significantly associated with better reduced monthly migraine days than the sham/control groups did; in the episodic migraine subgroup, anode tDCS over Oz + cathode over Cz (a-tDCS-Oz + c-tDCS-Cz; MD = -1.90 days, 95%CIs: − 2.27 to − 1.53) and transcutaneous auricular vagus nerve stimulation (taVNS; MD = -1.80 days, 95%CIs: − 3.38 to − 0.22) were significantly associated with better reduced monthly migraine days than the sham/control groups did (eFigure 2A-B, eFigure 3A-B, eTable 5B-C, and eTable 6A-B).

### Primary outcome: posttreatment response rate

The main result of the NMA revealed that the most interventions yielded significantly better response rates than the sham/control groups. These interventions included high frequency tONS over Oz (hf-tONS-Oz; RR = 9.00, 95%CIs: 1.24 to 65.16], low frequency tONS over Oz (lf-tONS-Oz; RR = 8.00, 95%CIs: 1.09 to 58.71), alternating frequency tONS over Oz (af-tONS-Oz; RR = 8.00, 95%CIs: 1.09 to 58.71), supraorbital transcutaneous stimulator over Afz (STS-Afz; RR = 3.15, 95%CIs: 1.15 to 8.69), percutaneous electrical nerve stimulation over Fp1Fp2 (PENS-Fp1Fp2; RR = 3.00, 95%CIs: 1.09 to 8.29), high frequency rTMS over F3 (hf-TMS-F3; RR = 2.41, 95%CIs: 1.58 to 3.68), single session high frequency rTMS over F3 (single-hf-TMS-F3; RR = 2.11, 95%CIs: 1.22 to 3.67), bilateral vagus nerve stimulation (Bi-nVNS; RR = 1.78, 95%CIs: 1.08 to 2.94), and single-pulse TMS over Oz (sTMS-Oz; RR = 1.78, 95%CIs: 1.09 to 2.90) (Table 2, Fig. 2B, and Fig. 3B (Forest plot of primary outcome: response rate)). According to the SUCRA results, hf-tONS-Oz yielded the highest response rate among all the interventions (eTable 5D).

**Table 2:** League table of the response rate

hf-tONS-Oz	1.13 (0.53,2.37)	1.13 (0.53,2.37)									*9.00 (1.24,65.16)
1.13 (0.53,2.37)	lf-tONS-Oz	1.00 (0.46,2.19)									*8.00 (1.09,58.71)
1.13 (0.53,2.37)	1.00 (0.46,2.19)	af-tONS-Oz									*8.00 (1.09,58.71)
2.85 (0.31,26.37)	2.54 (0.27,23.72)	2.54 (0.27,23.72)	STS-Afz								*3.15 (1.15,8.69)
3.00 (0.32,27.77)	2.67 (0.28,24.98)	2.67 (0.28,24.98)	1.05 (0.25,4.42)	PENS-Fp1Fp2							*3.00 (1.09,8.29)
3.73 (0.49,28.22)	3.31 (0.43,25.42)	3.31 (0.43,25.42)	1.31 (0.44,3.92)	1.24 (0.41,3.73)	hf-TMS-F3		1.14 (0.80,1.63)				*2.41 (1.58,3.68)
4.13 (0.35,49.06)	3.67 (0.30,44.08)	3.67 (0.30,44.08)	1.45 (0.24,8.74)	1.38 (0.23,8.33)	1.11 (0.24,5.19)	a-tDCS-Oz+c-tDCS-Cz					2.18 (0.49,9.65)
4.26 (0.55,33.26)	3.79 (0.48,29.95)	3.79 (0.48,29.95)	1.49 (0.47,4.73)	1.42 (0.45,4.51)	1.14 (0.80,1.63)	1.03 (0.21,5.05)	single-hf-TMS-F3				
5.06 (0.66,38.91)	4.50 (0.58,35.04)	4.50 (0.58,35.04)	1.77 (0.58,5.47)	1.69 (0.55,5.21)	1.36 (0.71,2.59)	1.23 (0.26,5.87)	1.19 (0.57,2.48)	sTMS-Oz			*1.78 (1.09,2.90)
5.05 (0.66,38.95)	4.49 (0.58,35.07)	4.49 (0.58,35.07)	1.77 (0.57,5.48)	1.68 (0.54,5.23)	1.36 (0.70,2.61)	1.23 (0.26,5.88)	1.19 (0.56,2.50)	1.00 (0.50,2.01)	Bi-nVNS		*1.78 (1.08,2.94)
6.91 (0.93,51.46)	6.14 (0.81,46.35)	6.14 (0.81,46.35)	2.42 (0.83,7.04)	2.30 (0.79,6.72)	*1.85 (1.08,3.18)	1.67 (0.36,7.70)	1.62 (0.85,3.10)	1.36 (0.75,2.47)	1.37 (0.75,2.50)	Rt-nVNS	1.30 (0.93,1.83)
*9.00 (1.24,65.16)	*8.00 (1.09,58.71)	*8.00 (1.09,58.71)	*3.15 (1.15,8.69)	*3.00 (1.09,8.29)	*2.41 (1.58,3.68)	2.18 (0.49,9.65)	*2.11 (1.22,3.67)	*1.78 (1.09,2.90)	*1.78 (1.08,2.94)	1.30 (0.93,1.83)	Sham/Control

Pairwise (upper-right portion) and network (lower-left portion) meta-analysis results are presented as estimate effect sizes for the outcome of response rate. Interventions are reported in order of mean ranking of treatment response, and outcomes are expressed as response rate ratio (RR) (95% confidence intervals). For the pairwise meta-analyses, RR of more than 1 indicate that the treatment specified in the row got better response than that specified in the column. For the network meta-analysis (NMA), RR of more than 1 indicate that the treatment specified in the column got better response than that specified in the row. Bold results marked with * indicate statistical significance.

*Abbreviation*: 95%CI 95% confidence interval, *af-tONS-Oz* Alternative frequency tONS over Oz, *a-tDCS-C3+c-tDCS-Fp2* Anode tDCS over C3 + cathode tDCS over Fp2, a*-tDCS-F3+c-tDCS-Fp2* Anode tDCS over F3 + cathode tDCS over Fp2, *a-tDCS-Oz+c-tDCS-Cz* Anode tDCS over Oz + cathode over Cz, *Bi-nVNS* Bilateral vagus nerve stimulation, *c-tDCS-C4+a-tDCS-arm* Cathode tDCS over C4 + anode at left upper arm, *c-tDCS-CP4+a-tDCS-arm* Cathode tDCS over CP4 + anode at left upper arm, *c-tDCS-Oz+a-tCDS-Cz* Cathode tDCS over Oz + anode tCDS over Cz, *dTMS-F3* Deep TMS-F3, *ES* Effect size, *hf-TMS-C3* High frequency rTMS over C3, *hf-TMS-F3* High frequency rTMS over F3, *hf-tONS-Oz* High frequency tONS over Oz, *lf-tONS-Oz* Low frequency tONS over Oz, *MD* Mean difference, *NMA* Network meta-analysis, *nVNS* Noninvasive vagus nerve stimulation, *PENS* percutaneous electrical nerve stimulation, *PENS-Fp1Fp2* Percutaneous electrical nerve stimulation over Fp1Fp2, *RCT* randomized controlled trial, *RR* Rate ratio, *rTMS* Repetitive transcranial magnetic stimulation, *Rt-nVNS*: right vagus nerve stimulation, *Sham/Control* Sham control or waiting list, *single-hf-TMS-F3* Single session high frequency rTMS over F3, *SMD* Standardized mean difference, *sTMS* Single-pulse TMS, *sTMS-Oz* single-pulse TMS over Oz, *STS*: supraorbital transcutaneous stimulation, *STS-Afz* Supraorbital transcutaneous stimulator over Afz, *SUCRA* Surface under the cumulative ranking curve, *taVNS* Transcutaneous auricular vagus nerve stimulation, *tDCS* Transcranial direct current stimulation, *TMS* Transcranial magnetic stimulation, *tONS* Transcutaneous occipital nerve stimulation

The assumption of transitivity was verified using the interaction test [15, 59]. There was no significantly different sham therapy effect between nVNS sham therapy and rTMS sham therapy (*p* = 0.432; eFigure 1B).

The subgroup analysis based on chronic migraine or episodic migraine revealed similar findings. In the chronic migraine subgroup, none of the investigated NIBS was associated with significantly different response rate compared to the sham/control groups did; in the episodic migraine subgroup, STS-Afz (RR = 3.15, 95%CIs: 1.15 to 8.69), PENS-Fp1Fp2 (RR = 3.00, 95%CIs: 1.09 to 8.29), Bi-nVNS (RR = 1.78, 95%CIs: 1.08 to 2.94), and sTMS-Oz (RR = 1.78, 95%CIs: 1.09 to 2.90) were significantly associated with better response rate than the sham/control groups did (eFigure 2C-D, eFigure 3C-D, eTable 5E-F, and eTable 6C-D).

### Secondary outcome: changes of migraine pain severity

The main result of the NMA revealed that the most investigated interventions were associated with significantly greater improvements in migraine pain severity than the sham/control treatments did, including c-tDCS-CP4 + a-tDCS-arm (SMD = -4.15, 95%CIs: − 5.31 to − 3.00), c-tDCS-C4 + a-tDCS-arm (SMD = -3.63, 95%CIs: − 4.70 to − 2.56), single session high frequency rTMS over F3 (single-hf-TMS-F3; SMD = − 2.64, 95%CIs: − 4.35 to − 0.93), hf-TMS-F3 (SMD = − 2.22, 95%CIs: − 3.87 to − 0.57), deep TMS-F3 (dTMS-F3; SMD = − 1.47, 95%CIs: − 2.69 to − 0.24), hf-tONS-Oz (SMD = − 0.99, 95%CIs: − 1.93 to − 0.05), lf-tONS-Oz (SMD = − 0.95, 95%CIs: − 1.56 to − 0.34), af-tONS-Oz (SMD = − 0.75, 95%CIs: − 1.35 to − 0.14), and transcutaneous auricular vagus nerve stimulation (taVNS; SMD = − 0.68, 95%CIs: − 1.21 to − 0.15) (eTable 6E, eFigure 2E, and eFigure 3E). According to the SUCRA results, c-tDCS-CP4 + a-tDCS-arm achieved the highest improvement in migraine pain severity among all interventions (eTable 5G).

### Secondary outcome: change of frequency of rescue medication use

We analyzed five articles with five individual treatment arms for changes in frequency of rescue medication use. None of the investigated noninvasive brain and nerve stimulations yielded significant change in the frequency of rescue medication use (eTable 5H, eTable 6F, eFigure 2F, and eFigure 3F).

### Acceptability with respect to drop-out rate

We investigated 13 articles with 15 individual treatment arms in the NMA for acceptability. Only af-tONS-Oz (RR = 0.36, 95%CIs: 0.16 to 0.82) yielded a significantly lower drop-out rate than the sham/control group did (eTable 6G, eFigure 2G, and eFigure 3G). According to the SUCRA results, af-tONS-Oz yielded the lowest drop-out rate among all the interventions (eTable 5I).

### Risk of bias and publication bias

We found that 82.0% (109/133 items), 9.0% (12/133 items), and 9.0% (12/133 items) of the included studies had low, unclear, and high risk of bias, respectively. The vague reporting of allocation concealment and blindness of the outcome assessment contributed to the risk of bias (eFigures 4A-4B).

Funnel plots of publication bias across the included studies (eFigures 5A-J) revealed general symmetry, and the results of Egger’s test indicated no significant publication bias among the articles included in the NMA. In general, the current NMA do not exhibit inconsistency, whether local inconsistency (assessed using the loop-specific approach and the node-splitting method) or global inconsistency (assessed using the design-by-treatment method) (eTable 7–8). The GRADE rating revealed that the quality of evidence of the most comparison in the current NMA ranged from low to medium (eTable 9A-B).

## Discussion

The main finding of the present NMA is that, among all the investigated non-invasive brain/nerve stimulation methods, the specific protocols of tDCS and rTMS (i.e. c-tDCS-CP4 + a-tDCS-arm, hf-TMS-C3, c-tDCS-C4 + a-tDCS-arm, and hf-TMS-F3) were associated with significantly better improvement in monthly migraine days than the sham/control did. In addition, the most of the non-invasive brain/nerve stimulation methods significantly improved response rates in migraine prophylaxis. The hf-TMS-C3 and hf-tONS-Oz were associated with the most effectiveness in outcomes of monthly migraine days and response rate, respectively. The main findings would not be changed in the subgroup analysis of chronic migraine/episodic migraine. Furthermore, c-tDCS-CP4 + a-tDCS-arm, in addition to significantly improving monthly migraine days, were most effective among the interventions in improving migraine pain severity. Finally, all interventions did not significantly decrease drop-out rate, with the exception of af-tONS-Oz.

The current NMA agreed with the significantly positive findings in the previous traditional meta-analysis [31, 32, 34, 35], which demonstrated the potentially beneficial effect by nVNS, tONS, PENS, and rTMS in migraine prophylaxis respectively. Beyond the traditional meta-analyses, the present study leveraged the advantages of NMA design to provide more comprehensive evidence, doing so by conducting individual comparisons between different treatment arms. This method contrasts with the pooling of all treatment arms into one group, as is done in traditional meta-analyses. Furthermore, we furnished evidence that noninvasive brain and nerve stimulation is as acceptable to patients, if not more so, than the sham/control group. Such evidence indicates that noninvasive brain and nerve stimulation is an optimal choice of treatment because of its safety, effectiveness, low drop-out rate, and less invasive method of administration [67, 70].

One of the major findings of the current NMA is that the specific protocols of tDCS and rTMS (i.e. c-tDCS-CP4 + a-tDCS-arm, hf-TMS-C3, c-tDCS-C4 + a-tDCS-arm, and hf-TMS-F3) were associated with significantly better improvement in monthly migraine days than the sham/control did. Among these treatment strategies, the hf-TMS-C3 was ranked to be associated with the most effectiveness in reducing monthly migraine days. The effect size data of hf-TMS-C3 was mainly extracted from one neuronavigation based rTMS study [75]. The stimulation over primary motor cortex (C3) might serve as the portal to reach deep brain structures; to be specific, the stimulation over primary motor cortex would drive corticothalamic output to the brainstem, spinal cord, and also limbic system so that it could modulate the pain matrix [75, 78]. Another treatment strategy of rTMS (i.e. hf-TMS-F3) was designed to target the left dorsolateral prefrontal cortex (F3). The rTMS stimulation over F3 could modulate the affective-emotional circuitry of pain, which was one of the major target in psychiatric research, especially depressive disorder [75, 79]. According to the previous review article, the migraine was highly associated with the depressive disorder [80]. The association between migraine severity and depression severity would have depression-dose dependent effect. To be specific, those who had severe depression had increased risk to become chronic migraine; whereas those mild depressive patients was mainly associated with episodic migraine [81]. Therefore, the potentially beneficial effect by hf-TMS-F3 might be partially derived from its efficacy on depressive disorder management. Additionally, tDCS stimulation over CP4 and C4 regions (i.e. c-tDCS-CP4 + a-tDCS-arm and c-tDCS-C4 + a-tDCS-arm, which represented stimulation over sensory cortex and primary motor cortex respectively) both contributed to significant improvement in monthly migraine days. Therefore, it should be not simply interpret tDCS effect with “activation or inhibition over specific cortex”. Rather, it might have to be interpreted as the theory of “neural noise”. The previous review of computational neuroscience modeling studies on stochastic resonance had shown that the neural noise resulted from tDCS, either in forms of depolarization or hyperpolarization in different target cortex, could contributed to whole brain function alteration [82, 83]. This theory could reflect the potentially beneficial effect by tDCS to improve brain pain-related plasticity [76, 84] through the mechanisms of brain network modulation and modification [76]. However, because these potential mechanism remained the stage of hypothesis, future rigorous and large-scale studies should be conducted to evaluate these hypothesized mechanisms.

The second major finding of the current NMA is that hf-tONS-Oz yielded the highest response rate in migraine prophylaxis among the noninvasive brain and nerve stimulation interventions. Scholars have long investigated the stimulation of the occipital nerve as a treatment for headaches and migraines of various types [6, 7], and findings in the literature for such an intervention were corroborated by a systematic review of occipital nerve stimulation for migraine treatment [85]. The mechanism underlying this treatment possibly relates to the aforementioned TGVT, which suggested that trigeminal nerve-TCC-ventroposteromedial thalamic nucleu cascade, among which converges the dura and cervical sensory afferents [86], was one of the major role of the migraine physiopathology [10]. Thus, modulation of the trigeminal nerve might potentially regulate the sensitization of the central pain pathway [87]. By delivering a continuous impulse, invasive occipital nerve stimulation can alleviate migraine-induced pain [85]. However, this traditional method of occipital nerve stimulation is expensive and invasive. Therefore, noninvasive tONS can ease migraine-induced pain and improve response rate with no adverse effects, unlike its traditional counterpart [22].

### Limitations

Our NMA has several limitations. First, some analyses in our NMA were limited by potential heterogeneity between studies with respect to participant characteristics, such as underlying diseases, concomitant medication, age, and heterogeneous diagnostic criteria and trial duration. Second, some included studies had small sample sizes, which may have resulted in less robust quantitative findings. Nonetheless, we included these studies because studies on our NMA topic are rare in the literature. Our comparison between different treatments allowed us to integrate findings on the effectiveness of noninvasive brain and nerve stimulation for migraine. Future studies should assess the efficacy of noninvasive brain and nerve stimulation interventions for the prevention of migraine in different medical settings, thus allowing clinicians to adapt preventive strategies to specific clinical conditions. Third, because of the weak network structure of our NMA, especially for some secondary outcomes, our results should be interpreted with caution. Fourth, some of the included RCTs did not apply a sham control [19, 69], and a placebo effect could therefore have affected their findings. Furthermore, among trials with a sham control, different sham therapy effects from different modes of sham control should be considered as a potential source of bias (e.g., the significant sham therapy effect from nVNS sham therapy). Fifth, the RCTs adopted different definitions for the primary outcomes. For example, because not all the migraine patients could clearly classify the current headache episode into migraine or other-type of headache, the RCTs applying headache diary might have some potentially methodological limitation. Therefore, the NMA based on headache diary might have potentially methodological heterogeneity despite of the non-significant finding in the inconsistency test or assumption test. Similar issue could be noted in the other primary outcome, the response rate, which included a 50% reduction in migraine frequency or the pain-free rate. Although our NMA found no obvious inconsistency, these different definitions for response might have contributed to bias in our analysis. Finally, the most RCTs in the current NMA had relatively short follow-up durations (mean follow-up duration = 11.4 weeks). Future studies with longer follow-up periods are thus warranted.

## Conclusions

The present NMA demonstrated that the hf-TMS-C3 and hf-tONS-Oz were associated with the most effectiveness in outcomes of monthly migraine days and response rate, respectively. Also, c-tDCS-CP4 + a-tDCS-arm, in addition to significantly improving monthly migraine days, were most effective among the interventions in improving migraine pain severity. The main findings would not be changed in the subgroup analysis of chronic migraine/episodic migraine. Finally, all interventions did not significantly decrease drop-out rate, with the exception of af-tONS-Oz. Because of the limitations of the small sample sizes, heterogeneous primary outcomes and study design among the included RCTs, and relatively short follow-up durations, our findings imply the need for future large-scale RCTs with longer follow-up durations; these will allow us to better determine the preventive effects of noninvasive brain/nerve stimulation in patients with migraine.

## Supplementary Information

**Additional file 1.**

**Additional file 2.**

## Data Availability

The data of the current study would be available upon reasonable request.

## Abbreviations

95%CI: 95% confidence interval
af-tONS-Oz: Alternating frequency tONS over Oz
a-tDCS-C3 + c-tDCS-Fp2: Anode tDCS over C3 + cathode tDCS over Fp2
a-tDCS-F3 + c-tDCS-Fp2: Anode tDCS over F3 + cathode tDCS over Fp2
a-tDCS-Oz + c-tDCS-Cz: anode tDCS over Oz + cathode over Cz
Bi-nVNS: Bilateral vagus nerve stimulation
c-tDCS-C4 + a-tDCS-arm: Cathode tDCS over C4 + anode at left upper arm
c-tDCS-CP4 + a-tDCS-arm: Cathode tDCS over CP4 + anode at left upper arm
c-tDCS-Oz + a-tCDS-Cz: Cathode tDCS over Oz + anode tCDS over Cz
dTMS-F3: Deep TMS-F3
ES: Effect size
hf-TMS-C3: High frequency rTMS over C3
hf-TMS-F3: High frequency rTMS over F3
hf-tONS-Oz: High frequency tONS over Oz
lf-tONS-Oz: Low frequency tONS over Oz
MD: Mean difference
NMA: Network meta-analysis
nVNS: Noninvasive vagus nerve stimulation
PENS: Percutaneous electrical nerve stimulation
PENS-Fp1Fp2: Percutaneous electrical nerve stimulation over Fp1Fp2
RCT: Randomized controlled trial
RR: Rate ratio
rTMS: Repetitive transcranial magnetic stimulation
Rt-nVNS: Right vagus nerve stimulation
Sham/Control: Sham control or waiting list
single-hf-TMS-F3: Single session high frequency rTMS over F3
SMD: Standardized mean difference
sTMS: Single-pulse TMS
sTMS-Oz: Single-pulse TMS over Oz
STS: Supraorbital transcutaneous stimulation
STS-Afz: Supraorbital transcutaneous stimulator over Afz
SUCRA: Surface under the cumulative ranking curve
taVNS: Transcutaneous auricular vagus nerve stimulation
tDCS: Transcranial direct current stimulation
TMS: Transcranial magnetic stimulation
tONS: Transcutaneous occipital nerve stimulation
