# Supplementary material for: Effectiveness and acceptability of noninvasive brain and nerve stimulation techniques for migraine prophylaxis: a network meta-analysis of randomized controlled trials
Source: J Headache Pain. 2022 Feb 20;23(1):28. doi: 10.1186/s10194-022-01401-3 (PMC8903676; doi:10.1186/s10194-022-01401-3)
Supplement: Supplementary file 1 — Additional file 1. [file 10194_2022_1401_MOESM1_ESM.pdf]

eTable 1: PRISMA 2020 checklist of current meta-analysis

Section and Topic	Item #	Checklist item	Page where item is reported
**TITLE**			
Title	1	Identify the report as a systematic review.	1
**ABSTRACT**			
Abstract	2	See the PRISMA 2020 for Abstracts checklist.	6
**INTRODUCTION**			
Rationale	3	Describe the rationale for the review in the context of existing knowledge.	8-9
Objectives	4	Provide an explicit statement of the objective(s) or question(s) the review addresses.	8-9
**METHODS**			
Eligibility criteria	5	Specify the inclusion and exclusion criteria for the review and how studies were grouped for the syntheses.	10-11
Information sources	6	Specify all databases, registers, websites, organisations, reference lists and other sources searched or consulted to identify studies. Specify the date when each source was last searched or consulted.	10-11
Search strategy	7	Present the full search strategies for all databases, registers and websites, including any filters and limits used.	10-11
Selection process	8	Specify the methods used to decide whether a study met the inclusion criteria of the review, including how many reviewers screened each record and each report retrieved, whether they worked independently, and if applicable, details of automation tools used in the process.	10-11
Data collection process	9	Specify the methods used to collect data from reports, including how many reviewers collected data from each report, whether they worked independently, any processes for obtaining or confirming data from study investigators, and if applicable, details of automation tools used in the process.	10-11
Data items	10a	List and define all outcomes for which data were sought. Specify whether all results that were compatible with each outcome domain in each study were sought (e.g. for all measures, time points, analyses), and if not, the methods used to decide which results to collect.	10-11
	10b	List and define all other variables for which data were sought (e.g. participant and intervention characteristics, funding sources). Describe any assumptions made about any missing or unclear information.	11-12
Study risk of bias assessment	11	Specify the methods used to assess risk of bias in the included studies, including details of the tool(s) used, how many reviewers assessed each study and whether they worked independently, and if applicable, details of automation tools used in the process.	11-12
Effect measures	12	Specify for each outcome the effect measure(s) (e.g. risk ratio, mean difference) used in the synthesis or presentation of results.	11-12
Synthesis methods	13a	Describe the processes used to decide which studies were eligible for each synthesis (e.g. tabulating the study intervention characteristics and comparing against the planned groups for each synthesis (item #5)).	11-12
	13b	Describe any methods required to prepare the data for presentation or synthesis, such as handling of missing summary statistics, or data conversions.	11-12
	13c	Describe any methods used to tabulate or visually display results of individual studies and syntheses.	12-13
	13d	Describe any methods used to synthesize results and provide a rationale for the choice(s). If meta-analysis was performed, describe the model(s), method(s) to identify the presence and extent of statistical heterogeneity, and software package(s) used.	12-13
	13e	Describe any methods used to explore possible causes of heterogeneity among study results (e.g. subgroup analysis, meta-regression).	12-13
	13f	Describe any sensitivity analyses conducted to assess robustness of the synthesized results.	12-13
Reporting bias assessment	14	Describe any methods used to assess risk of bias due to missing results in a synthesis (arising from reporting biases).	12-13
Certainty assessment	15	Describe any methods used to assess certainty (or confidence) in the body of evidence for an outcome.	12-13
**RESULTS**			
Study selection	16a	Describe the results of the search and selection process, from the number of records identified in the search to the number of studies included in the review, ideally using a flow diagram.	14-15, Fig 1, eTab 2
	16b	Cite studies that might appear to meet the inclusion criteria, but which were excluded, and explain why they were excluded.	14-15, eTab 3
Study	17	Cite each included study and present its characteristics.	14-15, eTab 4

Section and Topic	Item #	Checklist item	Page where item is reported
characteristics			
Risk of bias in studies	18	Present assessments of risk of bias for each included study.	14-15, eFig 4
Results of individual studies	19	For all outcomes, present, for each study: (a) summary statistics for each group (where appropriate) and (b) an effect estimate and its precision (e.g. confidence/credible interval), ideally using structured tables or plots.	14-15, eTab 4
Results of syntheses	20a	For each synthesis, briefly summarise the characteristics and risk of bias among contributing studies.	14-15, eFig 4
	20b	Present results of all statistical syntheses conducted. If meta-analysis was done, present for each the summary estimate and its precision (e.g. confidence/credible interval) and measures of statistical heterogeneity. If comparing groups, describe the direction of the effect.	15-16, Fig 3, eFig 2
	20c	Present results of all investigations of possible causes of heterogeneity among study results.	15-17, eTab 7-8
	20d	Present results of all sensitivity analyses conducted to assess the robustness of the synthesized results.	15-17, eFig 3
Reporting biases	21	Present assessments of risk of bias due to missing results (arising from reporting biases) for each synthesis assessed.	15-17, eFig 4
Certainty of evidence	22	Present assessments of certainty (or confidence) in the body of evidence for each outcome assessed.	15-17, eTab 9
**DISCUSSION**			
Discussion	23a	Provide a general interpretation of the results in the context of other evidence.	18-19
	23b	Discuss any limitations of the evidence included in the review.	19-20
	23c	Discuss any limitations of the review processes used.	19-20
	23d	Discuss implications of the results for practice, policy, and future research.	20
**OTHER INFORMATION**			
Registration and protocol	24a	Provide registration information for the review, including register name and registration number, or state that the review was not registered.	7
	24b	Indicate where the review protocol can be accessed, or state that a protocol was not prepared.	7
	24c	Describe and explain any amendments to information provided at registration or in the protocol.	7
Support	25	Describe sources of financial or non-financial support for the review, and the role of the funders or sponsors in the review.	24
Competing interests	26	Declare any competing interests of review authors.	24
Availability of data, code and other materials	27	Report which of the following are publicly available and where they can be found: template data collection forms; data extracted from included studies; data used for all analyses; analytic code; any other materials used in the review.	24

The current checklist followed the latest PRISMA 2020 guideline [1].

Reference

[1] Page MJ, McKenzie JE, Bossuyt PM, Boutron I, Hoffmann TC, Mulrow CD, et al. The PRISMA 2020 statement: an updated guideline for reporting systematic reviews. Bmj 2021;372:n71.

eTable 2: Key word applied in each database and result

Database	Keyword	Limit	Date	Result
PubMed	(deep transcranial magnetic stimulation OR dTMS OR repetitive transcranial magnetic stimulation OR rTMS OR TMS OR non-invasive brain stimulation OR theta burst stimulation OR transcranial direct current stimulation OR TBS OR tDCS OR vagus nerve stimulation OR vagal nerve stimulation OR tVNS OR nVNS OR VNS OR static magnetic field stimulation OR SMS OR tSMS) AND (migraine OR migrain* OR migraine disorder) AND (random OR randomized OR randomised)	N/A	2021/6/4	105
Embase	(deep transcranial magnetic stimulation OR dTMS OR repetitive transcranial magnetic stimulation OR rTMS OR TMS OR non-invasive brain stimulation OR theta burst stimulation OR transcranial direct current stimulation OR TBS OR tDCS OR vagus nerve stimulation OR vagal nerve stimulation OR tVNS OR nVNS OR VNS OR static magnetic field stimulation OR SMS OR tSMS) AND (migraine OR migrain* OR migraine disorder) AND (random OR randomized OR randomised)	N/A	2021/6/4	247
ClinicalKey	(non-invasive brain stimulation) AND (migraine OR migraine disorder)	RCTs	2021/6/4	17
Cochrane CENTRAL	(deep transcranial magnetic stimulation OR dTMS OR repetitive transcranial magnetic stimulation OR rTMS OR TMS OR non-invasive brain stimulation OR theta burst stimulation OR transcranial direct current stimulation OR TBS OR tDCS OR vagus nerve stimulation OR vagal nerve stimulation OR tVNS OR nVNS OR VNS OR static magnetic field stimulation OR SMS OR tSMS) AND (migraine OR migrain* OR migraine disorder) AND (random OR randomized OR randomised)	N/A	2021/6/4	173
ProQuest	(deep transcranial magnetic stimulation OR dTMS OR repetitive transcranial magnetic stimulation OR rTMS OR TMS OR non-invasive brain stimulation OR theta burst stimulation OR transcranial direct current stimulation OR TBS OR tDCS OR vagus nerve stimulation OR vagal nerve stimulation OR tVNS OR nVNS OR VNS OR static magnetic field stimulation OR SMS OR tSMS) AND (migraine OR migrain* OR migraine disorder) AND (random OR randomized OR randomised)	N/A	2021/6/4	3521
ScienceDirect	(non-invasive brain stimulation) AND (migraine OR migraine disorder)	research article	2021/6/4	805
Web of Science	(deep transcranial magnetic stimulation OR dTMS OR repetitive transcranial magnetic stimulation OR rTMS OR TMS OR non-invasive brain stimulation OR theta burst stimulation OR transcranial direct current stimulation OR TBS OR tDCS OR vagus nerve stimulation OR vagal nerve stimulation OR tVNS OR nVNS OR VNS OR static magnetic field stimulation OR SMS OR tSMS) AND (migraine OR migrain* OR migraine disorder) AND (random OR randomized OR randomised)	Article	2021/6/4	260
ClinicalTrials.gov	(non-invasive brain stimulation) AND (migraine OR migraine disorder)	N/A	2021/6/4	1

Abbreviation: N/A: not apply

eTable 3: Excluded studies and reasons

Reason	Numbers	Reference
All patients were comorbid with medication overuse, which was inconsistent with the other studies and violate the similarity hypothesis	3	[1-3]
Cross-over study and not provided the detailed outcome data at the end of 1st block	1	[4]
Duplicate samples with other trials included in the current network meta-analysis	3	[5-7]
Lack of adequate control	2	[8, 9]
Meta-analysis	10	[10-19]
Not migraine patients	22	[20-41]
Not non-invasive brain/nerve stimulation intervention	1	[42]
Not randomized controlled trial	6	[43-48]
Not related to outcome of interest	18	[49-66]
Not specific localization of target cortex of rTMS	1	[67]
Not specific localization of electrode of tDCS	3	[68-70]
Review article	4	[71-74]
Study protocol but not result of a study	1	[75]
The baseline migraine history was unbalanced between two treatment groups, which would have potential risk of violation of randomization	1	[76]

Abbreviation: tDCS: transcranial direct current stimulation

References:

1. De Icco R, Putorti A, De Paoli I, Ferrara E, Cremascoli R, Terzaghi M et al: (2021) Anodal transcranial direct current stimulation in chronic migraine and medication overuse headache: A pilot double-blind randomized sham-controlled trial. Clin Neurophysiol 132(1):126-136.
2. Granato A, Fantini J, Monti F, Furlanis G, Musho Ilbeh S, Semenic M et al: (2019) Dramatic placebo effect of high frequency repetitive TMS in treatment of chronic migraine and medication overuse headache. J Clin Neurosci 60:96-100.
3. Grazzi L, Usai S, Bolognini N, Grignani E, Sansone E, Tramacere I et al: (2020) No efficacy of transcranial direct current stimulation on chronic migraine with medication overuse: A double blind, randomised clinical trial. Cephalalgia 40(11):1202-1211.

4. Ahdab R, Mansour AG, Khazen G, El-Khoury C, Sabbouh TM, Salem M et al: (2019) Cathodal Transcranial Direct Current Stimulation of the Occipital cortex in Episodic Migraine: A Randomized Sham-Controlled Crossover Study. J Clin Med 9(1).
5. Martelletti P, Barbanti P, Grazzi L, Pierangeli G, Rainero I, Geppetti P et al: (2018) Consistent effects of non-invasive vagus nerve stimulation (nVNS) for the acute treatment of migraine: additional findings from the randomized, sham-controlled, double-blind PRESTO trial. The journal of headache and pain 19(1):101.
6. Grazzi L, Tassorelli C, de Tommaso M, Pierangeli G, Martelletti P, Rainero I et al: (2018) Practical and clinical utility of non-invasive vagus nerve stimulation (nVNS) for the acute treatment of migraine: a post hoc analysis of the randomized, sham-controlled, double-blind PRESTO trial. The journal of headache and pain 19(1):98.
7. Almaraz AC, Dilli E, Dodick DW: (2010) The effect of prophylactic medications on TMS for migraine aura. Headache 50(10):1630-1633.
8. Shehata HS, Esmail EH, Abdelalim A, El-Jaafary S, Elmazny A, Sabbah A et al: (2016) Repetitive transcranial magnetic stimulation versus botulinum toxin injection in chronic migraine prophylaxis: a pilot randomized trial. J Pain Res 9:771-777.
9. Clarke BM, Upton AR, Kamath MV, Al-Harbi T, Castellanos CM: (2006) Transcranial magnetic stimulation for migraine: clinical effects. The journal of headache and pain 7(5):341-346.
10. de Coo IF, Marin JC, Silberstein SD, Friedman DI, Gaul C, McClure CK et al: (2019) Differential efficacy of non-invasive vagus nerve stimulation for the acute treatment of episodic and chronic cluster headache: A meta-analysis. Cephalalgia 39(8):967-977.
11. Tao H, Wang T, Dong X, Guo Q, Xu H, Wan Q: (2018) Effectiveness of transcutaneous electrical nerve stimulation for the treatment of migraine: a meta-analysis of randomized controlled trials. The journal of headache and pain 19(1):42.
12. Lan L, Zhang X, Li X, Rong X, Peng Y: (2017) The efficacy of transcranial magnetic stimulation on migraine: a meta-analysis of randomized controlled trails. The journal of headache and pain 18(1):86.
13. Brigo F, Storti M, Tezzon F, Manganotti P, Nardone R: (2013) Primary visual cortex excitability in migraine: a systematic review with meta-analysis. Neurol Sci 34(6):819-830.
14. Brigo F, Storti M, Nardone R, Fiaschi A, Bongiovanni LG, Tezzon F et al: (2012) Transcranial magnetic stimulation of visual cortex in migraine patients: a systematic review with meta-analysis. The journal of headache and pain 13(5):339-349.
15. Cai G, Xia Z, Charvet L, Xiao F, Datta A, Androulakis XM: (2021) A Systematic Review and Meta-Analysis on the Efficacy of Repeated Transcranial Direct Current Stimulation for Migraine. J Pain Res 14:1171-1183.
16. Feng Y, Zhang B, Zhang J, Yin Y: (2019) Effects of Non-invasive Brain Stimulation on Headache Intensity and Frequency of Headache Attacks in Patients With Migraine: A Systematic Review and Meta-Analysis. Headache 59(9):1436-1447.
17. Lai YH, Huang YC, Huang LT, Chen RM, Chen C: (2020) Cervical Noninvasive Vagus Nerve Stimulation for Migraine and Cluster Headache: A Systematic Review and Meta-Analysis. Neuromodulation 23(6):721-731.
18. Moisset X, Pereira B, Ciampi de Andrade D, Fontaine D, Lanteri-Minet M, Mawet J: (2020) Neuromodulation techniques for acute and preventive migraine

treatment: a systematic review and meta-analysis of randomized controlled trials. The journal of headache and pain 21(1):142.
19. Shirahige L, Melo L, Nogueira F, Rocha S, Monte-Silva K: (2016) Efficacy of Noninvasive Brain Stimulation on Pain Control in Migraine Patients: A Systematic Review and Meta-Analysis. Headache 56(10):1565-1596.
20. De Icco R, Martinelli D, Bitetto V, Fresia M, Liebler E, Sandrini G et al: (2018) Peripheral vagal nerve stimulation modulates the nociceptive withdrawal reflex in healthy subjects: A randomized, cross-over, sham-controlled study. Cephalalgia 38(10):1658-1664.
21. Gaul C, Magis D, Liebler E, Straube A: (2017) Effects of non-invasive vagus nerve stimulation on attack frequency over time and expanded response rates in patients with chronic cluster headache: a post hoc analysis of the randomised, controlled PREVA study. The journal of headache and pain 18(1):22.
22. Gaul C, Diener HC, Silver N, Magis D, Reuter U, Andersson A et al: (2016) Non-invasive vagus nerve stimulation for PREVention and Acute treatment of chronic cluster headache (PREVA): A randomised controlled study. Cephalalgia 36(6):534-546.
23. Donnell A, T DN, Lawrence M, Gupta V, Zieba T, Truong DQ et al: (2015) High-Definition and Non-invasive Brain Modulation of Pain and Motor Dysfunction in Chronic TMD. Brain stimulation 8(6):1085-1092.
24. Dall'Agnol L, Medeiros LF, Torres IL, Deitos A, Brietzke A, Laste G et al: (2014) Repetitive transcranial magnetic stimulation increases the corticospinal inhibition and the brain-derived neurotrophic factor in chronic myofascial pain syndrome: an explanatory double-blinded, randomized, sham-controlled trial. The journal of pain : official journal of the American Pain Society 15(8):845-855.
25. George MS, Raman R, Benedek DM, Pelic CG, Grammer GG, Stokes KT et al: (2014) A two-site pilot randomized 3 day trial of high dose left prefrontal repetitive transcranial magnetic stimulation (rTMS) for suicidal inpatients. Brain stimulation 7(3):421-431.
26. Barrett DW, Gonzalez-Lima F: (2013) Transcranial infrared laser stimulation produces beneficial cognitive and emotional effects in humans. Neuroscience 230:13-23.
27. Busch V, Zeman F, Heckel A, Menne F, Ellrich J, Eichhammer P: (2013) The effect of transcutaneous vagus nerve stimulation on pain perception--an experimental study. Brain stimulation 6(2):202-209.
28. Duschek S, Schuepbach D, Doll A, Werner NS, Reyes del Paso GA: (2011) Self-regulation of cerebral blood flow by means of transcranial Doppler sonography biofeedback. Annals of behavioral medicine : a publication of the Society of Behavioral Medicine 41(2):235-242.
29. Burns J, Ouvrier RA, Yiu EM, Joseph PD, Kornberg AJ, Fahey MC et al: (2009) Ascorbic acid for Charcot-Marie-Tooth disease type 1A in children: a randomised, double-blind, placebo-controlled, safety and efficacy trial. Lancet Neurol 8(6):537-544.
30. Goadsby PJ, de Coo IF, Silver N, Tyagi A, Ahmed F, Gaul C et al: (2018) Non-invasive vagus nerve stimulation for the acute treatment of episodic and chronic cluster headache: A randomized, double-blind, sham-controlled ACT2 study. Cephalalgia 38(5):959-969.
31. Silberstein SD, Mechtler LL, Kudrow DB, Calhoun AH, McClure C, Saper JR et al: (2016) Non-Invasive Vagus Nerve Stimulation for the ACute Treatment of Cluster Headache: Findings From the Randomized, Double-Blind, Sham-Controlled ACT1 Study. Headache 56(8):1317-1332.
32. Umezaki Y, Badran BW, DeVries WH, Moss J, Gonzales T, George MS: (2016) The Efficacy of Daily Prefrontal Repetitive Transcranial Magnetic Stimulation (rTMS) for Burning Mouth Syndrome (BMS): A Randomized Controlled Single-blind Study. Brain stimulation 9(2):234-242.

33. Landeros-Weisenberger A, Mantovani A, Motlagh MG, de Alvarenga PG, Katsovich L, Leckman JF et al: (2015) Randomized Sham Controlled Double-blind Trial of Repetitive Transcranial Magnetic Stimulation for Adults With Severe Tourette Syndrome. Brain stimulation 8(3):574-581.
34. Plazier M, Ost J, Stassijns G, De Ridder D, Vanneste S: (2015) C2 Nerve Field Stimulation for the Treatment of Fibromyalgia: A Prospective, Double-blind, Randomized, Controlled Cross-over Study. Brain stimulation 8(4):751-757.
35. Steenbergen L, Sellaro R, Stock AK, Verkuil B, Beste C, Colzato LS: (2015) Transcutaneous vagus nerve stimulation (tVNS) enhances response selection during action cascading processes. European neuropsychopharmacology : the journal of the European College of Neuropsychopharmacology 25(6):773-778.
36. Kreuzer PM, Schecklmann M, Lehner A, Wetter TC, Poeppl TB, Rupprecht R et al: (2015) The ACDC pilot trial: targeting the anterior cingulate by double cone coil rTMS for the treatment of depression. Brain stimulation 8(2):240-246.
37. Li X, Malcolm RJ, Huebner K, Hanlon CA, Taylor JJ, Brady KT et al: (2013) Low frequency repetitive transcranial magnetic stimulation of the left dorsolateral prefrontal cortex transiently increases cue-induced craving for methamphetamine: a preliminary study. Drug Alcohol Depend 133(2):641-646.
38. Hwang DS, Kim HK, Seo JC, Shin IH, Kim DH, Kim YS: (2011) Sympathomodulatory effects of Saam acupuncture on heart rate variability in night-shift-working nurses. Complement Ther Med 19 Suppl 1:S33-40.
39. Levkovitz Y, Roth Y, Harel EV, Braw Y, Sheer A, Zangen A: (2007) A randomized controlled feasibility and safety study of deep transcranial magnetic stimulation. Clin Neurophysiol 118(12):2730-2744.
40. Villamar MF, Wivatvongvana P, Patumanond J, Bikson M, Truong DQ, Datta A et al: (2013) Focal modulation of the primary motor cortex in fibromyalgia using 4x1-ring high-definition transcranial direct current stimulation (HD-tDCS): immediate and delayed analgesic effects of cathodal and anodal stimulation. The journal of pain : official journal of the American Pain Society 14(4):371-383.
41. Mansour AG, Ahdab R, Khazen G, El-Khoury C, Sabbouh TM, Salem M et al: (2020) Transcranial Direct Current Stimulation of the Occipital Cortex in Medication Overuse Headache: A Pilot Randomized Controlled Cross-Over Study. J Clin Med 9(4).
42. Kapicioglu S, Gokce E, Kapicioglu Z, Ovali E: (1997) Treatment of migraine attacks with a long-acting somatostatin analogue (octreotide, SMS 201-995). Cephalalgia 17(1):27-30.
43. Hansen JM, Goadsby PJ, Charles AC: (2016) Variability of clinical features in attacks of migraine with aura. Cephalalgia 36(3):216-224.
44. Bohotin V, Fumal A, Vandenheede M, Bohotin C, Schoenen J: (2003) Excitability of visual V1-V2 and motor cortices to single transcranial magnetic stimuli in migraine: a reappraisal using a figure-of-eight coil. Cephalalgia 23(4):264-270.
45. Beh SC, Friedman DI: (2019) Acute vestibular migraine treatment with noninvasive vagus nerve stimulation. Neurology 93(18):e1715-e1719.
46. Neverdahl JP, Omland PM, Uglem M, Engstrom M, Sand T: (2017) Reduced motor cortical inhibition in migraine: A blinded transcranial magnetic stimulation study. Clin Neurophysiol 128(12):2411-2418.
47. Grazzi L, Egeo G, Calhoun AH, McClure CK, Liebler E, Barbanti P: (2016) Non-invasive Vagus Nerve Stimulation (nVNS) as mini-prophylaxis for menstrual/menstrually related migraine: an open-label study. The journal of headache and pain 17(1):91.
48. Chen PR, Lai KL, Fuh JL, Chen SP, Wang PN, Liao KK et al: (2016) Efficacy of continuous theta burst stimulation of the primary motor cortex in reducing

migraine frequency: A preliminary open-label study. J Chin Med Assoc 79(6):304-308.
49. Cosentino G, Di Marco S, Ferlisi S, Valentino F, Capitano WM, Fierro B et al: (2018) Intracortical facilitation within the migraine motor cortex depends on the stimulation intensity. A paired-pulse TMS study. The journal of headache and pain 19(1):65.
50. Conforto AB, Moraes MS, Amaro E, Jr., Young WB, Lois LA, Goncalves AL et al: (2012) Increased variability of motor cortical excitability to transcranial magnetic stimulation in migraine: a new clue to an old enigma. The journal of headache and pain 13(1):29-37.
51. Brighina F, Palermo A, Panetta ML, Daniele O, Aloisio A, Cosentino G et al: (2009) Reduced cerebellar inhibition in migraine with aura: a TMS study. Cerebellum 8(3):260-266.
52. Gunaydin S, Soysal A, Atay T, Arpaci B: (2006) Motor and occipital cortex excitability in migraine patients. Can J Neurol Sci 33(1):63-67.
53. Gerwig M, Niehaus L, Kastrup O, Stude P, Diener HC: (2005) Visual cortex excitability in migraine evaluated by single and paired magnetic stimuli. Headache 45(10):1394-1399.
54. Aurora SK, Welch KM, Al-Sayed F: (2003) The threshold for phosphenes is lower in migraine. Cephalalgia 23(4):258-263.
55. Bohotin V, Fumal A, Vandenheede M, Gerard P, Bohotin C, Maertens de Noordhout A et al: (2002) Effects of repetitive transcranial magnetic stimulation on visual evoked potentials in migraine. Brain 125(Pt 4):912-922.
56. Aurora SK, Cao Y, Bowyer SM, Welch KM: (1999) The occipital cortex is hyperexcitable in migraine: experimental evidence. Headache 39(7):469-476.
57. Afra J, Mascia A, Gerard P, Maertens de Noordhout A, Schoenen J: (1998) Interictal cortical excitability in migraine: a study using transcranial magnetic stimulation of motor and visual cortices. Ann Neurol 44(2):209-215.
58. Aurora SK, Ahmad BK, Welch KM, Bhardhwaj P, Ramadan NM: (1998) Transcranial magnetic stimulation confirms hyperexcitability of occipital cortex in migraine. Neurology 50(4):1111-1114.
59. Lozano-Soto E, Soto-Leon V, Sabbarese S, Ruiz-Alvarez L, Sanchez-Del-Rio M, Aguilar J et al: (2018) Transcranial static magnetic field stimulation (tSMS) of the visual cortex decreases experimental photophobia. Cephalalgia 38(8):1493-1497.
60. Uglem M, Omland PM, Engstrom M, Gravdahl GB, Linde M, Hagen K et al: (2016) Non-invasive cortical modulation of experimental pain in migraine. Clin Neurophysiol 127(6):2362-2369.
61. Khedr EM, Ahmed MA, Mohamed KA: (2006) Motor and visual cortical excitability in migraineurs patients with or without aura: transcranial magnetic stimulation. Neurophysiol Clin 36(1):13-18.
62. Mulleners WM, Chronicle EP, Palmer JE, Koehler PJ, Vredeveld JW: (2001) Visual cortex excitability in migraine with and without aura. Headache 41(6):565-572.
63. Luo W, Zhang Y, Yan Z, Liu X, Hou X, Chen W et al: (2020) The Instant Effects of Continuous Transcutaneous Auricular Vagus Nerve Stimulation at Acupoints on the Functional Connectivity of Amygdala in Migraine without Aura: A Preliminary Study. Neural Plast 2020:8870589.
64. Rocha S, Rodrigues MCA, Mendonca MB, Nogueira F, Boudoux C, Melo L et al: (2021) Could cathodal transcranial direct current stimulation modulate the power spectral density of alpha-band in migrainous occipital lobe? Neurosci Lett 742:135539.

65. Vecchio E, Ricci K, Montemurno A, Delussi M, Invitto S, de Tommaso M: (2016) Effects of left primary motor and dorsolateral prefrontal cortex transcranial direct current stimulation on laser-evoked potentials in migraine patients and normal subjects. Neurosci Lett 626:149-157.
66. Teo WP, Kannan A, Loh PK, Chew E, Sharma VK, Chan YC: (2014) Poor Tolerance of Motor Cortex rTMS in Chronic Migraine. Journal of clinical and diagnostic research : JCDR 8(9):MM01-02.
67. Bhola R, Kinsella E, Giffin N, Lipscombe S, Ahmed F, Weatherall M et al: (2015) Single-pulse transcranial magnetic stimulation (sTMS) for the acute treatment of migraine: evaluation of outcome data for the UK post market pilot program. The journal of headache and pain 16:535.
68. Rocha S, Melo L, Boudoux C, Foerster A, Araujo D, Monte-Silva K: (2015) Transcranial direct current stimulation in the prophylactic treatment of migraine based on interictal visual cortex excitability abnormalities: A pilot randomized controlled trial. J Neurol Sci 349(1-2):33-39.
69. Dasilva AF, Mendonca ME, Zaghi S, Lopes M, Dossantos MF, Spierings EL et al: (2012) tDCS-induced analgesia and electrical fields in pain-related neural networks in chronic migraine. Headache 52(8):1283-1295.
70. Dalla Volta G, Marceglia S, Zavarise P, Antonaci F: (2020) Cathodal tDCS Guided by Thermography as Adjunctive Therapy in Chronic Migraine Patients: A Sham-Controlled Pilot Study. Front Neurol 11:121.
71. Blech B, Starling AJ, Marks LA, Wingerchuk DM, O'Carroll CB: (2020) Is Noninvasive Vagus Nerve Stimulation a Safe and Effective Alternative to Medication for Acute Migraine Control? Neurologist 25(4):97-100.
72. Leahu P, Matei A, Groppa S: (2018) Transcranial magnetic stimulation in migraine prophylaxis. J Med Life 11(2):175-176.
73. Stilling JM, Monchi O, Amoozegar F, Debert CT: (2019) Transcranial Magnetic and Direct Current Stimulation (TMS/tDCS) for the Treatment of Headache: A Systematic Review. Headache 59(3):339-357.
74. Reuter U, McClure C, Liebler E, Pozo-Rosich P: (2019) Non-invasive neuromodulation for migraine and cluster headache: a systematic review of clinical trials. Journal of neurology, neurosurgery, and psychiatry 90(7):796-804.
75. Mohamad Safiai NI, Amir NA, Basri H, Inche Mat LN, Hoo FK, Yusof Khan AHK et al: (2020) Effectiveness and tolerability of repetitive transcranial magnetic stimulation for preventive treatment of episodic migraine: a single-centre, randomised, double-blind, sham-controlled phase 2 trial (Magnet-EM). Trials 21(1):923.
76. Straube A, Ellrich J, Eren O, Blum B, Ruscheweyh R: (2015) Treatment of chronic migraine with transcutaneous stimulation of the auricular branch of the vagal nerve (auricular t-VNS): a randomized, monocentric clinical trial. The journal of headache and pain 16:543.

eTable 4: Characteristics of the included studies

Study name	Baseline illness	Comparison	Subjects	mean age	female %	Treatment/ Follow up duration	CM/EM	Exclude Psychiatry	Country
Kumar, A. (2021)[1]	migraine without aura	High frequency rTMS left primary motor cortex<br>Sham control	10<br>10	33.2±8.2<br>33.8±7.2	60.0<br>50.0	2 weeks + 12 weeks	CM	Exclude	India
Zhang, Y. (2021)[2]	migraine without aura	taVNS-left cymba concha<br>Sham control	33<br>26	30.0±6.5<br>31.0±8.3	69.7<br>88.5	4 weeks + 0 weeks	EM	n/s	China
Pohl, H. (2020)[3]	migraine with or without aura	Anode tDCS-Oz + Cathode tDCS-Cz<br>Sham control	11<br>12	41.0±15.0<br>34.0±10.0	90.1<br>100.0	4 weeks + 4 weeks	EM	n/s	Switzerland
Rahimi, M.D. (2020)[4]	migraine with or without aura	Cathode tDCS-C4 + Anode tDCS-left upper arm<br>Cathode tDCS-CP4 + Anode tDCS-left upper arm<br>Sham control	15<br>15<br>15	35.5±12.0	88.9	10 weeks + 52 week	n/s	n/s	Iran
Diener, H.C. (2019)[5]	migraine with or without aura	nVNS : right and left vagus nerve<br>Sham control	165<br>167	43.5±11.1<br>41.4±12.3	86.1<br>82.6	12 weeks + 0 week	EM	Exclude	Multiple countries
Tassorelli C (2018)[6]	episodic migraine with/without aura	nVNS : right and left vagus nerve<br>Sham control	122<br>126	38.8±11.0<br>39.6±11.8	79.2<br>74.0	4 weeks + 0 week	EM	Exclude	Italy
Andrade, S.M. (2017)[7]	chronic refractory migraine	Anode tDCS-C3 + Cathode tDCS-Fp2<br>Anode tDCS-F3 + Cathode tDCS-Fp2<br>Sham control	6<br>4<br>3	32.3±14.0<br>31.5±13.9<br>34.5±13.4	50.0<br>50.0<br>66.7	4 weeks + 0 week	CM	Exclude	Brazil
Li, H. (2017)[8]	migraine with or without aura	PENS<br>Sham control	31<br>31	35.9±10.6<br>37.1±11.4	90.3<br>93.5	12 weeks + 0 week	EM	Exclude	China
Liu, Y. (2017)[9]	migraine without aura	tONS: 2Hz<br>tONS: 100Hz<br>tONS: 2/100Hz<br>Sham control	22<br>22<br>22<br>22	37.6±10.5<br>35.9±9.9<br>39.5±11.0<br>44.3±8.3	81.8<br>72.7<br>81.8<br>81.8	4 weeks + 12 weeks	n/s	n/s	China

Kalita, J. (2016)[10]	patients with chronic daily headache due to chronic migraine and long duration chronic tension-type headache	High frequency rTMS left frontal three session<br>High frequency rTMS left frontal single session	52<br>46	33.4±8.1<br>30.1±9.0	80.8<br>80.4	4 weeks + 8 week	CM	Exclude	India
Rapinesi, C. (2016)[11]	patients with chronic migraine	dTMS: 10Hz Left DLPFC<br>treatment as usual	7<br>7	53.3±5.8<br>50.1±7.8	71.4<br>57.1	4 weeks + 6 weeks	CM	n/s	Italy
Silberstein, S.D. (2016)[12]	chronic migraine with/without aura	nVNS : right vagus nerve<br>Sham control	30<br>29	40.5±14.2<br>38.8±11.1	86.7<br>86.2	8 weeks + 0 weeks	CM	n/s	USA
Conforto, A.B. (2014)[13]	chronic migraine with or without aura	High frequency rTMS Left DLPFC<br>Sham control	9<br>9	41.4±12.5<br>36.2±11.3	100.0<br>100.0	8 weeks + 0 week	CM	Exclude	Brazil
Misra, U.K. (2013)[14]	patients with chronic daily headache due to migraine	High frequency rTMS Left DLPFC<br>Sham control	50<br>50	35.6±10.1<br>35.1±10.4	88.0<br>88.0	4 weeks + 0 week	n/s	n/s	India
Schoenen, J. (2013)[15]	migraine with or without aura	Supraorbital transcutaneous stimulator<br>Sham control	34<br>33	34.6±11.0<br>39.1±9.9	91.2<br>90.9	13 weeks + 0 week	EM	Exclude	Belgium
Auvichayapat, P. (2012)[16]	migraine with or without aura	Anode tDCS-C3 + Cathode tDCS-Fp2<br>Sham control	22<br>20	28.6±6.8<br>35.1±13.5	70.0<br>70.6	3 weeks + 12 weeks	EM	Exclude	Thailand
Antal, A. (2011)[17]	migraine without aura, migraine with aura, or chronic migraine	Cathode tDCS-Oz + Anode tCDS-Cz<br>Sham control	15<br>15	33.2±10.4<br>32.3±12.3	92.3<br>84.6	6 weeks + 8 weeks	n/s	Exclude	Germany
Lipton, R.B. (2010)[18]	migraine with aura	Single-pulse TMS*<br>Sham control	82<br>82	38.8±11.2<br>40.1±10.8	81.7<br>76.8	12 weeks + 0 week	EM	n/s	USA
Brighina, F. (2004)[19]	chronic migraine with or without aura	High frequency rTMS Left DLPFC<br>Sham control	6<br>5	47.0±7.0	63.6	4 weeks + 4 week	CM	Exclude	Italy

*: in the study by Lipton, R.B. (2010), although it said single pulse for acute treatment, the authors allowed patients brining the machine back home, which exerts similar effect as the other non-invasive brain/nerve stimulation in migraine prophylaxis

Abbreviation: CM: chronic migraine; DLPFC: dorsolateral prefrontal cortex; dTMS: deep transcranial magnetic stimulation; EM: episodic migraine; n/s: not specified; nVNS: non-invasive vagus nerve stimulation; PENS: percutaneous electrical nerve stimulation over Fp1 and Fp2 region; taVNS: transcutaneous auricular vagus nerve stimulation; tDCS: transcranial direct current stimulation; TMS: transcranial magnetic stimulation; tONS: transcutaneous occipital nerve stimulation; USA: United States of America

References:

1. Kumar A, Mattoo B, Bhatia R, Kumaran S, Bhatia R: (2021) Neuronavigation based 10 sessions of repetitive transcranial magnetic stimulation therapy in chronic migraine: an exploratory study. Neurol Sci 42(1):131-139.
2. Zhang Y, Huang Y, Li H, Yan Z, Zhang Y, Liu X et al: (2021) Transcutaneous auricular vagus nerve stimulation (taVNS) for migraine: an fMRI study. Reg Anesth Pain Med 46(2):145-150.
3. Pohl H, Moisa M, Jung HH, Brenner K, Aschmann J, Riederer F et al: (2020) Long-Term Effects of Self-Administered Transcranial Direct Current Stimulation in Episodic Migraine Prevention: Results of a Randomized Controlled Trial. Neuromodulation.
4. Rahimi MD, Fadardi JS, Saeidi M, Bigdeli I, Kashiri R: (2020) Effectiveness of cathodal tDCS of the primary motor or sensory cortex in migraine: A randomized controlled trial. Brain stimulation 13(3):675-682.
5. Diener HC, Goadsby PJ, Ashina M, Al-Karagholi MA, Sinclair A, Mitsikostas D et al: (2019) Non-invasive vagus nerve stimulation (nVNS) for the preventive treatment of episodic migraine: The multicentre, double-blind, randomised, sham-controlled PREMIUM trial. Cephalalgia 39(12):1475-1487.
6. Tassorelli C, Grazzi L, de Tommaso M, Pierangeli G, Martelletti P, Rainero I et al: (2018) Noninvasive vagus nerve stimulation as acute therapy for migraine: The randomized PRESTO study. Neurology 91(4):e364-e373.
7. Andrade SM, de Brito Aranha REL, de Oliveira EA, de Mendonca C, Martins WKN, Alves NT et al: (2017) Transcranial direct current stimulation over the primary motor vs prefrontal cortex in refractory chronic migraine: A pilot randomized controlled trial. J Neurol Sci 378:225-232.
8. Li H, Xu QR: (2017) Effect of percutaneous electrical nerve stimulation for the treatment of migraine. Medicine 96(39):e8108.
9. Liu Y, Dong Z, Wang R, Ao R, Han X, Tang W et al: (2017) Migraine Prevention Using Different Frequencies of Transcutaneous Occipital Nerve Stimulation: A Randomized Controlled Trial. The journal of pain : official journal of the American Pain Society 18(8):1006-1015.
10. Kalita J, Laskar S, Bhoi SK, Misra UK: (2016) Efficacy of single versus three sessions of high rate repetitive transcranial magnetic stimulation in chronic migraine and tension-type headache. J Neurol 263(11):2238-2246.
11. Rapinesi C, Del Casale A, Scatena P, Kotzalidis GD, Di Pietro S, Ferri VR et al: (2016) Add-on deep Transcranial Magnetic Stimulation (dTMS) for the treatment of chronic migraine: A preliminary study. Neurosci Lett 623:7-12.
12. Silberstein SD, Calhoun AH, Lipton RB, Grosberg BM, Cady RK, Dorlas S et al: (2016) Chronic migraine headache prevention with noninvasive vagus nerve stimulation: The EVENT study. Neurology 87(5):529-538.
13. Conforto AB, Amaro E, Jr., Goncalves AL, Mercante JP, Guendler VZ, Ferreira JR et al: (2014) Randomized, proof-of-principle clinical trial of active transcranial magnetic stimulation in chronic migraine. Cephalalgia 34(6):464-472.
14. Misra UK, Kalita J, Bhoi SK: (2013) High-rate repetitive transcranial magnetic stimulation in migraine prophylaxis: a randomized, placebo-controlled study. J Neurol 260(11):2793-2801.
15. Schoenen J, Vandersmissen B, Jeangette S, Herroelen L, Vandenheede M, Gerard P et al: (2013) Migraine prevention with a supraorbital transcutaneous

stimulator: a randomized controlled trial. Neurology 80(8):697-704.

16. Auvichayapat P, Janyacharoen T, Rotenberg A, Tiamkao S, Krisanaprakornkit T, Sinawat S et al: (2012) Migraine prophylaxis by anodal transcranial direct current stimulation, a randomized, placebo-controlled trial. J Med Assoc Thai 95(8):1003-1012.

17. Antal A, Kriener N, Lang N, Boros K, Paulus W: (2011) Cathodal transcranial direct current stimulation of the visual cortex in the prophylactic treatment of migraine. Cephalalgia 31(7):820-828.

18. Lipton RB, Dodick DW, Silberstein SD, Saper JR, Aurora SK, Pearlman SH et al: (2010) Single-pulse transcranial magnetic stimulation for acute treatment of migraine with aura: a randomised, double-blind, parallel-group, sham-controlled trial. Lancet Neurol 9(4):373-380.

19. Brighina F, Piazza A, Vitello G, Aloisio A, Palermo A, Daniele O et al: (2004) rTMS of the prefrontal cortex in the treatment of chronic migraine: a pilot study. J Neurol Sci 227(1):67-71.

eTable 5A: SUCRA of the changes of frequency of migraine attack

Treatment	SUCRA
hf-TMS-C3	13.1
c-tDCS-CP4+a-tDCS-arm	14.5
c-tDCS-C4+a-tDCS-arm	18.2
hf-TMS-F3	25.3
single-hf-TMS-F3	38.4
a-tDCS-Oz+c-tDCS-Cz	57.0
c-tDCS-Oz+a-tCDS-Cz	57.9
taVNS	58.7
PENS-Fp1Fp2	60.1
lf-tONS-Oz	60.6
STS-Afz	61.5
Rt-nVNS	62.1
hf-tONS-Oz	62.1
af-tONS-Oz	62.8
a-tDCS-C3+c-tDCS-Fp2	72.6
Sham/Control	75.2

Sorted by order of mean rank of changes of frequency of migraine attack (the former, the better improvement in frequency of migraine attack)

eTable 5B: SUCRA of the changes of frequency of migraine attack: chronic migraine

Treatment	SUCRA
hf-TMS-F3	15.2
single-hf-TMS-F3	29.6
hf-TMS-C3	33.4
Rt-nVNS	74.9
Sham/Control	96.8

Sorted by order of mean rank of changes of frequency of migraine attack (the former, the better improvement in frequency of migraine attack)

eTable 5C: SUCRA of the changes of frequency of migraine attack: episodic migraine

Treatment	SUCRA
a-tDCS-Oz+c-tDCS-Cz	17.0
taVNS	22.9
PENS-Fp1Fp2	31.0
STS-Afz	37.8
Rt-nVNS	67.2
a-tDCS-C3+c-tDCS-Fp2	82.7
Sham/Control	91.4

Sorted by order of mean rank of changes of frequency of migraine attack (the former, the better improvement in frequency of migraine attack)

eTable 5D: SUCRA of the response rate

Treatment	SUCRA
hf-tONS-Oz	14.6
lf-tONS-Oz	19.3
af-tONS-Oz	20.1
STS-Afz	38.8
PENS-Fp1Fp2	38.9
hf-TMS-F3	45.6
a-tDCS-Oz+c-tDCS-Cz	56.3
single-hf-TMS-F3	56.3
sTMS-Oz	64.8
Bi-nVNS	65.2
Rt-nVNS	83.1
Sham/Control	97.0

Sorted by order of mean rank of response rate (the former, the better response rate)

eTable 5E: SUCRA of the response rate: chronic migraine

Treatment	SUCRA
hf-TMS-F3	23.5
single-hf-TMS-F3	43.0
Rt-nVNS	47.6
Sham/Control	85.9

Sorted by order of mean rank of response rate (the former, the better response rate)

eTable 5F: SUCRA of the response rate: episodic migraine

Treatment	SUCRA
PENS-Fp1Fp2	20.7
STS-Afz	21.4
a-tDCS-Oz+c-tDCS-Cz	42.8
sTMS-Oz	48.0
Bi-nVNS	48.5
Rt-nVNS	73.6
Sham/Control	95.0

Sorted by order of mean rank of response rate (the former, the better response rate)

eTable 5G: SUCRA of the severity of migraine

Treatment	SUCRA
c-tDCS-CP4+a-tDCS-arm	1.1
c-tDCS-C4+a-tDCS-arm	7.4
single-hf-TMS-F3	13.2
hf-TMS-F3	22.1
dTMS-F3	30.8
lf-tONS-Oz	41.3
hf-TMS-C3	41.9
af-tONS-Oz	51.4
taVNS	53.4
a-tDCS-F3+c-tDCS-Fp2	58.5
c-tDCS-Oz+a-tCDS-Cz	62.6
a-tDCS-Oz+c-tDCS-Cz	65.1
hf-tONS-Oz	65.7
Bi-nVNS	77.8
a-tDCS-C3+c-tDCS-Fp2	79.7
STS-Afz	84.6
Sham/Control	93.4

Sorted by order of mean rank of improvement of severity of migraine (the former, the better improvement of severity of migraine)

eTable 5H: SUCRA of the acute rescue medication usage

Treatment	SUCRA
hf-TMS-F3	27.8
PENS-Fp1Fp2	47.6
dTMS-F3	50.1
STS-Afz	52.2
a-tDCS-Oz+c-tDCS-Cz	55.4
Rt-nVNS	56.0
Sham/Control	61.0

Sorted by order of mean rank of improvement of frequency of acute rescue medication usage (the former, the less usage of acute rescue medication)

eTable 5I: SUCRA of the drop-out rate

Treatment	SUCRA
af-tONS-Oz	82.6
taVNS	77.7
a-tDCS-F3+c-tDCS-Fp2	70.7
hf-tONS-Oz	64.7
lf-tONS-Oz	64.6
Bi-nVNS	63.3
Rt-nVNS	47.7
a-tDCS-C3+c-tDCS-Fp2	42.0
STS-Afz	39.7
c-tDCS-Oz+a-tCDS-Cz	38.9
single-hf-TMS-F3	37.9
Sham/Control	33.5
sTMS-Oz	31.1
hf-TMS-F3	28.7
PENS-Fp1Fp2	27.1

Sorted by order of mean rank of drop-out rate (the former, the less drop-out rate)

Abbreviation: 95%CI: 95% confidence interval; af-tONS-Oz: alternating frequency tONS over Oz; a-tDCS-C3+c-tDCS-Fp2: anode tDCS over C3 + cathode tDCS over Fp2; a-tDCS-F3+c-tDCS-Fp2: anode tDCS over F3 + cathode tDCS over Fp2; a-tDCS-Oz+c-tDCS-Cz: anode tDCS over Oz + cathode over Cz; Bi-nVNS: bilateral vagus nerve stimulation; c-tDCS-C4+a-tDCS-arm: cathode tDCS over C4 + anode at left upper arm; c-tDCS-CP4+a-tDCS-arm: cathode tDCS over CP4 + anode at left upper arm; c-tDCS-Oz+a-tCDS-Cz: cathode tDCS over Oz + anode tCDS over Cz; dTMS-F3: deep TMS-F3; ES: effect size; hf-TMS-C3: high frequency rTMS over C3; hf-TMS-F3: high frequency rTMS over F3; hf-tONS-Oz: high frequency tONS over Oz; lf-tONS-Oz: low frequency tONS over Oz; MD: mean difference; NMA: network meta-analysis; nVNS: noninvasive vagus nerve stimulation; PENS: percutaneous electrical nerve stimulation; PENS-Fp1Fp2: percutaneous electrical nerve stimulation over Fp1Fp2; RCT: randomized controlled trial; RR: rate ratio; rTMS: repetitive transcranial magnetic stimulation; Rt-nVNS: right vagus nerve stimulation; Sham/Control: Sham control or waiting list; single-hf-TMS-F3: single session high frequency rTMS over F3; SMD: standardized mean difference; sTMS: single-pulse TMS; sTMS-Oz: single-pulse TMS over Oz; STS: supraorbital transcutaneous stimulation; STS-Afz: supraorbital transcutaneous stimulator over Afz; SUCRA: surface under the cumulative ranking curve; taVNS: transcutaneous auricular vagus nerve stimulation; tDCS: transcranial direct current stimulation; TMS: transcranial magnetic stimulation; tONS: transcutaneous occipital nerve stimulation

eTable 6A: League table of the outcome of changes in monthly migraine days: chronic migraine

hf-TMS-F3	-1.35 (-9.90,7.20)			***-10.97 (-16.85,-5.09)**
-1.35 (-9.97,7.27)	single-hf-TMS-F3			
-2.27 (-8.43,3.89)	-0.92 (-11.58,9.74)	hf-TMS-C3		***-8.70 (-10.41,-6.99)**
***-8.37 (-15.34,-1.39)**	-7.02 (-18.12,4.08)	***-6.10 (-10.26,-1.94)**	Rt-nVNS	-2.60 (-6.35,1.15)
***-10.97 (-17.09,-4.84)**	-9.62 (-20.37,1.13)	***-8.70 (-10.71,-6.69)**	-2.60 (-6.69,1.49)	Sham/Control

Pairwise (upper-right portion) and network (lower-left portion) meta-analysis results are presented as estimate effect sizes for the outcome of improvement of monthly migraine days. Interventions are reported in order of mean ranking of monthly migraine days improvement, and outcomes are expressed as mean difference (MD) (95% confidence intervals). For the pairwise meta-analyses, MD of less than 0 indicate that the treatment specified in the row got more improvement than that specified in the column. For the network meta-analysis (NMA), MD of less than 0 indicate that the treatment specified in the column got more improvement than that specified in the row. Bold results marked with * indicate statistical significance.

eTable 6B: League table of the outcome of changes in monthly migraine days: episodic migraine

a-tDCS-Oz+c-tDCS-Cz						***-1.90 (-2.27,-1.53)**
-0.10 (-1.72,1.52)	taVNS					***-1.80 (-3.38,-0.22)**
-0.40 (-2.00,1.20)	-0.30 (-2.52,1.92)	PENS-Fp1Fp2				-1.50 (-3.05,0.05)
-0.56 (-2.15,1.03)	-0.46 (-2.67,1.75)	-0.16 (-2.35,2.03)	STS-Afz			-1.34 (-2.89,0.21)
***-1.44 (-2.28,-0.60)**	-1.34 (-3.09,0.41)	-1.04 (-2.77,0.69)	-0.88 (-2.60,0.84)	Rt-nVNS		-0.46 (-1.22,0.30)
***-1.77 (-2.39,-1.15)**	***-1.67 (-3.33,-0.01)**	-1.37 (-3.00,0.26)	-1.21 (-2.84,0.42)	-0.33 (-1.24,0.58)	a-tDCS-C3+c-tDCS-Fp2	-0.13 (-0.63,0.37)
***-1.90 (-2.27,-1.53)**	***-1.80 (-3.38,-0.22)**	-1.50 (-3.05,0.05)	-1.34 (-2.89,0.21)	-0.46 (-1.22,0.30)	-0.13 (-0.63,0.37)	Sham/Control

Pairwise (upper-right portion) and network (lower-left portion) meta-analysis results are presented as estimate effect sizes for the outcome of improvement of monthly migraine days. Interventions are reported in order of mean ranking of monthly migraine days improvement, and outcomes are expressed as mean difference (MD) (95% confidence intervals). For the pairwise meta-analyses, MD of less than 0 indicate that the treatment specified in the row got more improvement than that specified in the column. For the network meta-analysis (NMA), MD of less than 0 indicate that the treatment specified in the column got more improvement than that specified in the row. Bold results marked with * indicate statistical significance.

eTable 6C: League table of the response rate: chronic migraine

hf-TMS-F3	1.14 (0.80,1.63)		6.00 (0.38,94.35)
1.14 (0.80,1.63)	single-hf-TMS-F3		
2.00 (0.06,68.21)	1.75 (0.05,60.77)	Rt-nVNS	3.00 (0.33,27.23)
6.00 (0.38,94.34)	5.25 (0.33,84.48)	3.00 (0.33,27.23)	Sham/Control

Pairwise (upper-right portion) and network (lower-left portion) meta-analysis results are presented as estimate effect sizes for the outcome of response rate. Interventions are reported in order of mean ranking of treatment response, and outcomes are expressed as response rate ratio (RR) (95% confidence intervals). For the pairwise meta-analyses, RR of more than 1 indicate that the treatment specified in the row got better response than that specified in the column. For the network meta-analysis (NMA), RR of more than 1 indicate that the treatment specified in the column got better response than that specified in the row. Bold results marked with * indicate statistical significance.

eTable 6D: League table of the response rate: episodic migraine

PENS-Fp1Fp2						***3.00 (1.09,8.29)**
0.95 (0.23,3.99)	STS-Afz					***3.15 (1.15,8.69)**
1.38 (0.23,8.33)	1.45 (0.24,8.74)	a-tDCS-Oz+c-tDCS-Cz				2.18 (0.49,9.65)
1.69 (0.55,5.21)	1.77 (0.58,5.47)	1.23 (0.26,5.87)	sTMS-Oz			***1.78 (1.09,2.90)**
1.68 (0.54,5.23)	1.77 (0.57,5.48)	1.23 (0.26,5.88)	1.00 (0.50,2.01)	Bi-nVNS		***1.78 (1.08,2.94)**
2.35 (0.80,6.87)	2.47 (0.85,7.20)	1.71 (0.37,7.86)	1.39 (0.77,2.53)	1.39 (0.76,2.56)	Rt-nVNS	1.28 (0.91,1.80)
***3.00 (1.09,8.29)**	***3.15 (1.15,8.69)**	2.18 (0.49,9.65)	***1.78 (1.09,2.90)**	***1.78 (1.08,2.94)**	1.28 (0.91,1.80)	Sham/Control

Pairwise (upper-right portion) and network (lower-left portion) meta-analysis results are presented as estimate effect sizes for the outcome of response rate. Interventions are reported in order of mean ranking of treatment response, and outcomes are expressed as response rate ratio (RR) (95% confidence intervals). For the pairwise meta-analyses, RR of more than 1 indicate that the treatment specified in the row got better response than that specified in the column. For the network meta-analysis (NMA), RR of more than 1 indicate that the treatment specified in the column got better response than that specified in the row. Bold results marked with * indicate statistical significance.

eTable 6E: League table of the migraine pain severity

c-tDCS-CP4+a-tDCS-arm	-0.52 (-1.24,0.21)															***-4.15 (-5.31,-3.00)**
-0.52 (-1.24,0.21)	c-tDCS-C4+a-tDCS-arm															***-3.63 (-4.70,-2.57)**
-1.51 (-3.57,0.55)	-0.99 (-3.01,1.02)	single-hf-TMS-F3	-0.42 (-0.88,0.03)													
-1.93 (-3.94,0.08)	-1.41 (-3.37,0.55)	-0.42 (-0.88,0.03)	hf-TMS-F3													***-2.22 (-3.87,-0.58)**
***-2.68 (-4.37,-1.00)**	***-2.17 (-3.79,-0.54)**	-1.18 (-3.28,0.93)	-0.76 (-2.81,1.30)	dTMS-F3												***-1.47 (-2.69,-0.24)**
***-3.20 (-4.51,-1.89)**	***-2.68 (-3.91,-1.45)**	-1.69 (-3.51,0.12)	-1.27 (-3.03,0.48)	-0.52 (-1.88,0.85)	lf-tONS-Oz		-0.20 (-0.80,0.39)					-0.45 (-1.05,0.14)				***-0.95 (-1.56,-0.34)**
***-3.16 (-4.65,-1.67)**	***-2.64 (-4.06,-1.22)**	-1.65 (-3.60,0.30)	-1.23 (-3.13,0.67)	-0.47 (-2.02,1.07)	0.04 (-1.08,1.16)	hf-TMS-C3										***-0.99 (-1.93,-0.05)**
***-3.40 (-4.71,-2.10)**	***-2.89 (-4.11,-1.66)**	***-1.90 (-3.71,-0.08)**	-1.47 (-3.23,0.28)	-0.72 (-2.08,0.65)	-0.20 (-0.79,0.39)	-0.25 (-1.36,0.87)	af-tONS-Oz					-0.25 (-0.84,0.34)				***-0.75 (-1.35,-0.15)**
***-3.47 (-4.74,-2.20)**	***-2.95 (-4.15,-1.76)**	***-1.96 (-3.75,-0.17)**	-1.54 (-3.27,0.19)	-0.79 (-2.12,0.55)	-0.27 (-1.08,0.54)	-0.31 (-1.39,0.77)	-0.07 (-0.87,0.73)	taVNS								***-0.68 (-1.21,-0.15)**
***-3.53 (-5.24,-1.83)**	***-3.02 (-4.67,-1.37)**	-2.03 (-4.15,0.10)	-1.61 (-3.68,0.47)	-0.85 (-2.61,0.91)	-0.33 (-1.73,1.07)	-0.38 (-1.95,1.20)	-0.13 (-1.53,1.27)	-0.06 (-1.43,1.30)	a-tDCS-F3+c-tDCS-Fp2					-0.37 (-1.64,0.91)		-0.71 (-2.25,0.82)
***-3.63 (-5.00,-2.27)**	***-3.12 (-4.41,-1.82)**	***-2.13 (-3.98,-0.27)**	-1.71 (-3.51,0.10)	-0.95 (-2.38,0.48)	-0.43 (-1.38,0.52)	-0.48 (-1.67,0.71)	-0.23 (-1.18,0.71)	-0.16 (-1.06,0.74)	-0.10 (-1.56,1.36)	c-tDCS-Oz+a-tCDS-Cz						-0.52 (-1.25,0.21)
***-3.67 (-5.09,-2.24)**	***-3.15 (-4.51,-1.80)**	***-2.16 (-4.06,-0.26)**	-1.74 (-3.58,0.11)	-0.98 (-2.46,0.50)	-0.47 (-1.50,0.56)	-0.51 (-1.77,0.75)	-0.26 (-1.29,0.76)	-0.20 (-1.18,0.79)	-0.13 (-1.64,1.38)	-0.03 (-1.14,1.07)	a-tDCS-Oz+c-tDCS-Cz					-0.48 (-1.31,0.35)
***-3.65 (-4.95,-2.35)**	***-3.14 (-4.36,-1.91)**	***-2.14 (-3.95,-0.34)**	-1.72 (-3.47,0.03)	-0.97 (-2.33,0.39)	-0.45 (-1.05,0.14)	-0.49 (-1.61,0.62)	-0.25 (-0.84,0.34)	-0.18 (-0.98,0.61)	-0.12 (-1.51,1.27)	-0.02 (-0.96,0.92)	0.02 (-1.01,1.04)	hf-tONS-Oz				-0.50 (-1.09,0.10)
***-3.91 (-5.09,-2.72)**	***-3.39 (-4.49,-2.29)**	***-2.40 (-4.13,-0.67)**	***-1.98 (-3.64,-0.31)**	-1.22 (-2.47,0.03)	*-0.71 (-1.37,-0.05)	-0.75 (-1.72,0.23)	-0.50 (-1.16,0.15)	-0.44 (-1.02,0.15)	-0.37 (-1.66,0.91)	-0.27 (-1.04,0.50)	-0.24 (-1.11,0.63)	-0.25 (-0.90,0.39)	Bi-nVNS			-0.24 (-0.50,0.01)
***-3.94 (-5.23,-2.66)**	***-3.43 (-4.63,-2.22)**	***-2.44 (-4.23,-0.64)**	***-2.01 (-3.75,-0.28)**	-1.26 (-2.60,0.09)	-0.74 (-1.57,0.08)	-0.79 (-1.88,0.31)	-0.54 (-1.36,0.28)	-0.47 (-1.24,0.30)	-0.41 (-1.62,0.80)	-0.31 (-1.23,0.61)	-0.27 (-1.28,0.73)	-0.29 (-1.11,0.52)	-0.04 (-0.65,0.57)	a-tDCS-C3+c-tDCS-Fp2		-0.21 (-0.76,0.35)
***-4.03 (-5.28,-2.78)**	***-3.51 (-4.68,-2.34)**	***-2.52 (-4.29,-0.75)**	***-2.10 (-3.81,-0.38)**	***-1.34 (-2.66,-0.03)**	***-0.83 (-1.60,-0.05)**	-0.87 (-1.93,0.19)	-0.62 (-1.39,0.14)	-0.56 (-1.27,0.16)	-0.49 (-1.84,0.85)	-0.39 (-1.27,0.48)	-0.36 (-1.32,0.60)	-0.38 (-1.14,0.39)	-0.12 (-0.66,0.42)	-0.08 (-0.82,0.65)	STS-Afz	-0.12 (-0.60,0.36)
***-4.15 (-5.31,-3.00)**	***-3.63 (-4.70,-2.56)**	***-2.64 (-4.35,-0.93)**	***-2.22 (-3.87,-0.57)**	***-1.47 (-2.69,-0.24)**	***-0.95 (-1.56,-0.34)**	***-0.99 (-1.93,-0.05)**	***-0.75 (-1.35,-0.14)**	***-0.68 (-1.21,-0.15)**	-0.62 (-1.88,0.64)	-0.52 (-1.25,0.21)	-0.48 (-1.31,0.35)	-0.50 (-1.09,0.10)	-0.24 (-0.50,0.01)	-0.21 (-0.76,0.35)	-0.12 (-0.60,0.36)	Sham/Control

Pairwise (upper-right portion) and network (lower-left portion) meta-analysis results are presented as estimate effect sizes for the outcome of improvement of migraine pain severity. Interventions are reported in order of mean ranking of migraine pain severity improvement, and outcomes are expressed as standardized mean difference (SMD) (95% confidence intervals). For the pairwise meta-analyses, SMD of less than 0 indicate that the treatment specified in the row got more improvement than that specified in the column. For the network meta-analysis (NMA), SMD of less than 0 indicate that the treatment specified in the column got more improvement than that specified in the row. Bold results marked with * indicate statistical significance.

eTable 6F: League table of changes of frequency of acute rescue medication usage

hf-TMS-F3						-10.70 (-29.74,8.35)
-6.90 (-39.18,25.39)	PENS-Fp1Fp2					***-3.80 (-5.26,-2.34)**
-7.56 (-39.86,24.75)	-0.66 (-37.45,36.13)	dTMS-F3				***-3.14 (-5.06,-1.22)**
-8.67 (-41.07,23.74)	-1.77 (-38.65,35.11)	-1.11 (-38.01,35.79)	STS-Afz			-2.03 (-5.16,1.10)
-10.80 (-43.05,21.46)	-3.90 (-40.64,32.84)	-3.24 (-40.01,33.53)	-2.13 (-38.98,34.72)	a-tDCS-Oz+c-tDCS-Cz		0.10 (-0.23,0.43)
-10.15 (-42.41,22.11)	-3.25 (-40.00,33.50)	-2.59 (-39.36,34.18)	-1.48 (-38.34,35.38)	0.65 (-36.07,37.37)	Rt-nVNS	-0.55 (-1.36,0.26)
-10.70 (-29.83,8.44)	-3.80 (-29.80,22.20)	-3.14 (-29.17,22.89)	-2.03 (-28.18,24.12)	0.10 (-25.86,26.06)	-0.55 (-26.52,25.42)	Sham/Control

Pairwise (upper-right portion) and network (lower-left portion) meta-analysis results are presented as estimate effect sizes for the outcome of improvement in frequency of acute rescue medication usage. Interventions are reported in order of mean ranking of treatment effect, and outcomes are expressed as standardized mean difference (SMD) (95% confidence intervals). For the pairwise meta-analyses, SMD of less than 0 indicate that the treatment specified in the row got better improvement in frequency of acute rescue medication usage than that specified in the column. For the network meta-analysis (NMA), SMD of less than 0 indicate that the treatment specified in the column got better improvement in frequency of acute rescue medication usage than that specified in the row. Bold results marked with * indicate statistical significance.

eTable 6G: League table of the tolerability in aspect of drop-out rate

af-tONS-Oz			0.63 (0.24,1.61)	0.63 (0.24,1.61)							0.36 (0.16,0.82)			
1.26 (0.12,13.30)	taVNS										0.28 (0.03,2.59)			
1.20 (0.05,28.54)	0.96 (0.02,41.51)	a-tDCS-F3+c-tDCS-Fp2					0.28 (0.02,4.66)				0.80 (0.02,32.13)			
0.63 (0.24,1.61)	0.50 (0.05,4.95)	0.52 (0.02,11.84)	hf-tONS-Oz	1.00 (0.46,2.19)							0.57 (0.30,1.08)			
0.63 (0.24,1.61)	0.50 (0.05,4.95)	0.52 (0.02,11.84)	1.00 (0.46,2.19)	lf-tONS-Oz							0.57 (0.30,1.08)			
0.69 (0.11,4.51)	0.55 (0.03,8.82)	0.58 (0.02,18.88)	1.11 (0.18,6.67)	1.11 (0.18,6.67)	Bi-nVNS						0.52 (0.10,2.77)			
0.44 (0.17,1.12)	0.35 (0.04,3.32)	0.37 (0.02,8.06)	0.71 (0.33,1.51)	0.71 (0.33,1.51)	0.64 (0.11,3.60)	Rt-nVNS					0.81 (0.53,1.23)			
0.39 (0.07,2.05)	0.31 (0.02,4.32)	0.32 (0.02,5.30)	0.62 (0.13,3.00)	0.62 (0.13,3.00)	0.56 (0.06,5.13)	0.88 (0.20,3.94)	a-tDCS-C3+c-tDCS-Fp2				0.92 (0.22,3.88)			
0.37 (0.08,1.72)	0.29 (0.02,3.80)	0.31 (0.01,8.51)	0.59 (0.14,2.50)	0.59 (0.14,2.50)	0.53 (0.06,4.45)	0.83 (0.21,3.26)	0.95 (0.14,6.59)	STS-Afz			0.97 (0.26,3.56)			
0.36 (0.05,2.65)	0.28 (0.02,4.99)	0.30 (0.01,10.48)	0.57 (0.08,3.95)	0.57 (0.08,3.95)	0.52 (0.04,6.16)	0.81 (0.12,5.26)	0.92 (0.09,9.40)	0.97 (0.10,9.12)	c-tDCS-Oz+a-tCDS-Cz		1.00 (0.16,6.20)			
0.36 (0.07,1.81)	0.28 (0.02,3.87)	0.30 (0.01,8.58)	0.57 (0.12,2.65)	0.57 (0.12,2.65)	0.52 (0.06,4.58)	0.81 (0.19,3.47)	0.92 (0.12,6.83)	0.97 (0.14,6.54)	1.00 (0.10,9.94)	single-hf-TMS-F3			0.85 (0.39,1.83)	
***0.36 (0.16,0.82)**	0.28 (0.03,2.59)	0.30 (0.01,6.34)	0.57 (0.30,1.08)	0.57 (0.30,1.08)	0.52 (0.10,2.77)	0.81 (0.53,1.23)	0.92 (0.22,3.88)	0.97 (0.26,3.56)	1.00 (0.16,6.20)	1.00 (0.25,4.05)	Sham/Control	0.92 (0.43,1.96)	0.85 (0.26,2.72)	0.75 (0.18,3.08)
0.33 (0.11,1.01)	0.26 (0.03,2.69)	0.27 (0.01,6.37)	0.52 (0.19,1.41)	0.52 (0.19,1.41)	0.47 (0.07,2.99)	0.74 (0.31,1.76)	0.84 (0.17,4.29)	0.89 (0.20,4.01)	0.92 (0.13,6.61)	0.92 (0.19,4.50)	0.92 (0.43,1.96)	sTMS-Oz		
0.30 (0.07,1.27)	0.24 (0.02,2.93)	0.25 (0.01,6.65)	0.48 (0.13,1.83)	0.48 (0.13,1.83)	0.44 (0.06,3.38)	0.68 (0.20,2.36)	0.78 (0.12,4.97)	0.82 (0.14,4.72)	0.85 (0.10,7.39)	0.85 (0.39,1.83)	0.85 (0.26,2.72)	0.92 (0.23,3.72)	hf-TMS-F3	
0.27 (0.05,1.38)	0.21 (0.02,2.93)	0.22 (0.01,6.48)	0.43 (0.09,2.02)	0.43 (0.09,2.02)	0.39 (0.04,3.47)	0.61 (0.14,2.64)	0.69 (0.09,5.18)	0.73 (0.11,4.96)	0.75 (0.07,7.53)	0.75 (0.10,5.48)	0.75 (0.18,3.08)	0.82 (0.16,4.06)	0.89 (0.14,5.53)	PENS-Fp1Fp2

Pairwise (upper-right portion) and network (lower-left portion) meta-analysis results are presented as estimate effect sizes for the outcome of tolerability in aspect of drop-out rate. Interventions are reported in order of mean ranking of tolerability, and outcomes are expressed as odds ratio (OR) (95% confidence intervals). For the pairwise meta-analyses, OR of less than 1 indicate that the treatment specified in the row got more tolerability than that specified in the column. For the network meta-analysis (NMA), OR of less than 1 indicate that the treatment specified in the column got more tolerability than that specified in the row. Bold results marked with * indicate statistical significance.

Abbreviation: 95%CI: 95% confidence interval; af-tONS-Oz: alternating frequency tONS over Oz; a-tDCS-C3+c-tDCS-Fp2: anode tDCS over C3 + cathode tDCS over Fp2; a-tDCS-F3+c-tDCS-Fp2: anode tDCS over F3 + cathode tDCS over Fp2; a-tDCS-Oz+c-tDCS-Cz: anode tDCS over Oz + cathode over Cz; Bi-nVNS: bilateral vagus nerve stimulation; c-tDCS-C4+a-tDCS-arm: cathode tDCS over C4 + anode at left upper arm; c-tDCS-CP4+a-tDCS-arm: cathode tDCS over CP4 + anode at left upper arm; c-tDCS-Oz+a-tCDS-Cz: cathode tDCS over Oz + anode tCDS over Cz; dTMS-F3: deep TMS-F3; ES: effect size; hf-TMS-C3: high frequency rTMS over C3; hf-TMS-F3: high frequency rTMS over F3; hf-tONS-Oz: high frequency tONS over Oz; lf-tONS-Oz: low frequency tONS over Oz; MD: mean difference; NMA: network meta-analysis; nVNS: noninvasive vagus nerve stimulation; PENS: percutaneous electrical nerve stimulation; PENS-Fp1Fp2: percutaneous electrical nerve stimulation over Fp1Fp2; RCT: randomized controlled trial; RR: rate ratio; rTMS: repetitive transcranial magnetic stimulation; Rt-nVNS: right vagus nerve stimulation; Sham/Control: Sham control or waiting list; single-hf-TMS-F3: single session high frequency rTMS over F3; SMD: standardized mean difference; sTMS: single-pulse TMS; sTMS-Oz: single-pulse TMS over Oz; STS: supraorbital transcutaneous stimulation; STS-Afz: supraorbital transcutaneous stimulator over Afz; SUCRA: surface under the cumulative ranking curve; taVNS: transcutaneous auricular vagus nerve stimulation; tDCS: transcranial direct current stimulation; TMS: transcranial magnetic stimulation; tONS: transcutaneous occipital nerve stimulation

eTable 7: Inconsistency of different intervention

Part 1: design-by-treatment and loop inconsistency model

Inconsistency model	chi^2	Prob>chi^2
	changes in monthly migraine days	
design-by-treatment	0.07	0.7950
loop inconsistency	0.07	0.7950
	response rate	
design-by-treatment	0.54	0.4627
loop inconsistency	0.54	0.4627
	migraine severity	
design-by-treatment	0.05	0.8295
loop inconsistency	3.28	0.0700
	changes in frequency of rescue medication use	
design-by-treatment	1.20	0.2732
loop inconsistency	1.20	0.2732
	Drop-out rate	
design-by-treatment	0.88	0.3493
loop inconsistency	0.18	0.6735

Part 2: side-splitting inconsistency model:

Part of changes in monthly migraine days

Side	symmetric		nosymmetric		Treatments used	
	P>z	tau	P>z	tau		
A B	.	.	.	.	A:	Sham/Control
A C	.	.	.	.	B:	taVNS
A D	.	.	.	.	C:	a-tDCS-Oz+c-tDCS-Cz
A E	.	.	.	.	D:	c-tDCS-Oz+a-tCDS-Cz
A F	.	.	.	.	E:	Rt-nVNS

A G	.	.	.	.	F:	c-tDCS-CP4+a-tDCS-arm
A H *	0.999	2.798451	0.999	2.798451	G:	hf-tONS-Oz
A J	.	.	.	.	H:	hf-TMS-F3
A K	.	.	.	.	I:	single-hf-TMS-F3
A L	.	.	.	.	J:	STS-Afz
A M	.	.	.	.	K:	a-tDCS-C3+c-tDCS-Fp2
A N	.	.	.	.	L:	c-tDCS-C4+a-tDCS-arm
A O	.	.	.	.	M:	PENS-Fp1Fp2
A P	.	.	.	.	N:	hf-TMS-C3
F L	.	.	.	.	O:	af-tONS-Oz
G O	.	.	.	.	P:	lf-tONS-Oz
G P	.	.	.	.		
H I *	1	2.798447	.	.		
O P	.	.	.	.		

Part of response rate

Side	symmetric		nosymmetric		Treatments used	
	P>z	tau	P>z	tau		
A B	.	.	.	.	A:	Sham/Control
A C	.	.	.	.	B:	a-tDCS-Oz+c-tDCS-Cz
A D	.	.	.	.	C:	PENS-Fp1Fp2
A E	.	.	.	.	D:	af-tONS-Oz
A F	.	.	.	.	E:	Rt-nVNS
A G	.	.	.	.	F:	Bi-nVNS
A H *	0.997	3.87E-05	0.997	3.87E-05	G:	sTMS-Oz
A J	.	.	.	.	H:	hf-TMS-F3
A K	.	.	.	.	I:	single-hf-TMS-F3
A L	.	.	.	.	J:	STS-Afz
D K	.	.	.	.	K:	hf-tONS-Oz

D L	.	.	.	.	L:	lf-tONS-Oz
H I *	0.998	3.16E-06	.	.		
K L	.	.	.	.		

Part of changes in migraine severity

Side	symmetric		nosymmetric		Treatments used	
	P>z	tau	P>z	tau		
A B	.	.	.	.	A:	Sham/Control
A C	.	.	.	.	B:	taVNS
A D	.	.	.	.	C:	dTMS-F3
A E	.	.	.	.	D:	c-tDCS-Oz+a-tCDS-Cz
A F	.	.	.	.	E:	a-tDCS-Oz+c-tDCS-Cz
A G	.	.	.	.	F:	Bi-nVNS
A H *	0.992	2.48E-07	0.992	2.48E-07	G:	c-tDCS-CP4+a-tDCS-arm
A J	.	.	.	.	H:	hf-TMS-F3
A K	.	.	.	.	I:	single-hf-TMS-F3
A L	.	.	.	.	J:	STS-Afz
A M	.	.	.	.	K:	a-tDCS-C3+c-tDCS-Fp2
A N	.	.	.	.	L:	c-tDCS-C4+a-tDCS-arm
A O *	0.83	0.003309	.	.	M:	af-tONS-Oz
A P	.	.	.	.	N:	hf-TMS-C3
A Q	.	.	.	.	O:	a-tDCS-F3+c-tDCS-Fp2
G L	.	.	.	.	P:	lf-tONS-Oz
H I *	0.82	5.73E-06	.	.	Q:	hf-tONS-Oz
K O *	0.83	0.003349	.	.		
M P	.	.	.	.		
M Q	.	.	.	.		
P Q	.	.	.	.		

Part of changes in frequency of rescue medication use

Side	symmetric		nosymmetric		Treatments used	
	P>z	tau	P>z	tau		
A B	.	.	.	.	A:	Sham/Control
A C	.	.	.	.	B:	a-tDCS-Oz+c-tDCS-Cz
A D	.	.	.	.	C:	dTMS-F3
A E	.	.	.	.	D:	PENS-Fp1Fp2
A F	.	.	.	.	E:	Rt-nVNS
A G	.	.	.	.	F:	STS-Afz
					G:	hf-TMS-F3

Part of drop-out

Side	symmetric		nosymmetric		Treatments used	
	P>z	tau	P>z	tau		
A B	.	.	.	.	A:	Sham/Control
A C	.	.	.	.	B:	taVNS
A D	.	.	.	.	C:	PENS-Fp1Fp2
A E	.	.	.	.	D:	c-tDCS-Oz+a-tCDS-Cz
A F	.	.	.	.	E:	Rt-nVNS
A G	.	.	.	.	F:	Bi-nVNS
A H *	1	9.72E-08	1	9.72E-08	G:	lf-tONS-Oz
A J	.	.	.	.	H:	hf-TMS-F3
A K	.	.	.	.	I:	single-hf-TMS-F3
A L	.	.	.	.	J:	STS-Afz
A M	.	.	.	.	K:	a-tDCS-C3+c-tDCS-Fp2
A N	.	.	.	.	L:	af-tONS-Oz
A O *	0.349	8.06E-08	.	.	M:	sTMS-Oz
G L	.	.	.	.	N:	hf-tONS-Oz
G N	.	.	.	.	O:	a-tDCS-F3+c-tDCS-Fp2
H I *	1	9.81E-07	.	.		

K O *	0.349	6.58E-11	.	.
L N	.	.	.	.

Abbreviation: 95%CI: 95% confidence interval; af-tONS-Oz: alternating frequency tONS over Oz; a-tDCS-C3+c-tDCS-Fp2: anode tDCS over C3 + cathode tDCS over Fp2; a-tDCS-F3+c-tDCS-Fp2: anode tDCS over F3 + cathode tDCS over Fp2; a-tDCS-Oz+c-tDCS-Cz: anode tDCS over Oz + cathode over Cz; Bi-nVNS: bilateral vagus nerve stimulation; c-tDCS-C4+a-tDCS-arm: cathode tDCS over C4 + anode at left upper arm; c-tDCS-CP4+a-tDCS-arm: cathode tDCS over CP4 + anode at left upper arm; c-tDCS-Oz+a-tCDS-Cz: cathode tDCS over Oz + anode tCDS over Cz; dTMS-F3: deep TMS-F3; ES: effect size; hf-TMS-C3: high frequency rTMS over C3; hf-TMS-F3: high frequency rTMS over F3; hf-tONS-Oz: high frequency tONS over Oz; lf-tONS-Oz: low frequency tONS over Oz; MD: mean difference; NMA: network meta-analysis; nVNS: noninvasive vagus nerve stimulation; PENS: percutaneous electrical nerve stimulation; PENS-Fp1Fp2: percutaneous electrical nerve stimulation over Fp1Fp2; RCT: randomized controlled trial; RR: rate ratio; rTMS: repetitive transcranial magnetic stimulation; Rt-nVNS: right vagus nerve stimulation; Sham/Control: Sham control or waiting list; single-hf-TMS-F3: single session high frequency rTMS over F3; SMD: standardized mean difference; sTMS: single-pulse TMS; sTMS-Oz: single-pulse TMS over Oz; STS: supraorbital transcutaneous stimulation; STS-Afz: supraorbital transcutaneous stimulator over Afz; SUCRA: surface under the cumulative ranking curve; taVNS: transcutaneous auricular vagus nerve stimulation; tDCS: transcranial direct current stimulation; TMS: transcranial magnetic stimulation; tONS: transcutaneous occipital nerve stimulation

eTable 8: Estimated between-studies standard deviation of different outcome

Outcome	Estimated between-studies standard deviation
Changes in monthly migraine days	2.7984465
Response rate	2.505e-06
Migraine severity	1.330e-07
Changes in frequency of rescue medication use	13.245631
Drop-out	5.714e-06

eTable 9A: Quality of evidence for primary outcome: changes in monthly migraine days

Comparisons	GRADE					
	Direct		Indirect		Network meta-analysis	
	Mean difference (95% CI)	The final rating of direct evidence	Co-efficiency (Standard error)	The final rating of indirect evidence	Mean difference (95% CI)	Overall quality of evidence
hf-TMS-C3 vs c-tDCS-CP4+a-tDCS-arm					0.03 (-8.74,8.80)	⊕◯◯◯ Very low
hf-TMS-C3 vs c-tDCS-C4+a-tDCS-arm					-0.70 (-9.45,8.05)	⊕◯◯◯ Very low
hf-TMS-C3 vs hf-TMS-F3					-2.42 (-10.17,5.33)	⊕◯◯◯ Very low
hf-TMS-C3 vs single-hf-TMS-F3					-3.77 (-16.55,9.01)	⊕◯◯◯ Very low
hf-TMS-C3 vs a-tDCS-Oz+c-tDCS-Cz					-6.80 (-14.75,1.15)	⊕◯◯◯ Very low
hf-TMS-C3 vs c-tDCS-Oz+a-tCDS-Cz					-6.96 (-17.68,3.76)	⊕◯◯◯ Very low
hf-TMS-C3 vs taVNS					-6.90 (-15.00,1.20)	⊕◯◯◯ Very low
hf-TMS-C3 vs PENS-Fp1Fp2					-7.20 (-15.29,0.89)	⊕◯◯◯ Very low
hf-TMS-C3 vs lf-tONS-Oz					-7.49 (-18.00,3.02)	⊕◯◯◯ Very low
hf-TMS-C3 vs STS-Afz					-7.36 (-15.45,0.73)	⊕◯◯◯ Very low
hf-TMS-C3 vs Rt-nVNS					***-7.36 (-14.53,-0.20)**	⊕⊕◯◯ Low
hf-TMS-C3 vs hf-tONS-Oz					-7.49 (-18.42,3.45)	⊕◯◯◯ Very low
hf-TMS-C3 vs af-tONS-Oz					-7.66 (-18.81,3.49)	⊕◯◯◯ Very low
hf-TMS-C3 vs a-tDCS-C3+c-tDCS-Fp2					***-8.57 (-16.53,-0.61)**	⊕⊕◯◯ Low
hf-TMS-C3 vs Sham/Control	***-8.70 (-10.41,-6.99)**	⊕⊕⊕◯ Medium			***-8.70 (-14.45,-2.95)**	⊕⊕⊕◯ Medium
c-tDCS-CP4+a-tDCS-arm vs c-tDCS-C4+a-tDCS-arm	-0.73 (-1.98,0.52)	⊕⊕◯◯ Low			-0.73 (-6.35,4.89)	⊕⊕◯◯ Low
c-tDCS-CP4+a-tDCS-arm vs hf-TMS-F3					-2.45 (-10.87,5.97)	⊕◯◯◯ Very low
c-tDCS-CP4+a-tDCS-arm vs single-hf-TMS-F3					-3.80 (-16.99,9.39)	⊕◯◯◯ Very low
c-tDCS-CP4+a-tDCS-arm vs a-tDCS-Oz+c-tDCS-Cz					-6.83 (-15.44,1.78)	⊕◯◯◯ Very low
c-tDCS-CP4+a-tDCS-arm vs c-tDCS-Oz+a-tCDS-Cz					-6.99 (-18.21,4.22)	⊕◯◯◯ Very low
c-tDCS-CP4+a-tDCS-arm vs taVNS					-6.93 (-15.67,1.81)	⊕◯◯◯ Very low
c-tDCS-CP4+a-tDCS-arm vs PENS-Fp1Fp2					-7.23 (-15.97,1.51)	⊕◯◯◯ Very low

c-tDCS-CP4+a-tDCS-arm vs lf-tONS-Oz					-7.52 (-18.53,3.50)	⊕◯◯◯ Very low
c-tDCS-CP4+a-tDCS-arm vs STS-Afz					-7.39 (-16.13,1.35)	⊕◯◯◯ Very low
c-tDCS-CP4+a-tDCS-arm vs Rt-nVNS					-7.39 (-15.28,0.49)	⊕◯◯◯ Very low
c-tDCS-CP4+a-tDCS-arm vs hf-tONS-Oz					-7.52 (-18.93,3.90)	⊕◯◯◯ Very low
c-tDCS-CP4+a-tDCS-arm vs af-tONS-Oz					-7.69 (-19.32,3.94)	⊕◯◯◯ Very low
c-tDCS-CP4+a-tDCS-arm vs a-tDCS-C3+c-tDCS-Fp2					-8.60 (-17.21,0.01)	⊕◯◯◯ Very low
c-tDCS-CP4+a-tDCS-arm vs Sham/Control	*-8.73 (-12.44,-5.02)	⊕⊕◯◯ Low			*-8.73 (-15.35,-2.11)	⊕⊕⊕◯ Medium
c-tDCS-C4+a-tDCS-arm vs hf-TMS-F3					-1.72 (-10.12,6.67)	⊕◯◯◯ Very low
c-tDCS-C4+a-tDCS-arm vs single-hf-TMS-F3					-3.07 (-16.25,10.11)	⊕◯◯◯ Very low
c-tDCS-C4+a-tDCS-arm vs a-tDCS-Oz+c-tDCS-Cz					-6.10 (-14.69,2.49)	⊕◯◯◯ Very low
c-tDCS-C4+a-tDCS-arm vs c-tDCS-Oz+a-tCDS-Cz					-6.26 (-17.46,4.94)	⊕◯◯◯ Very low
c-tDCS-C4+a-tDCS-arm vs taVNS					-6.20 (-14.92,2.52)	⊕◯◯◯ Very low
c-tDCS-C4+a-tDCS-arm vs PENS-Fp1Fp2					-6.50 (-15.22,2.22)	⊕◯◯◯ Very low
c-tDCS-C4+a-tDCS-arm vs lf-tONS-Oz					-6.79 (-17.78,4.21)	⊕◯◯◯ Very low
c-tDCS-C4+a-tDCS-arm vs STS-Afz					-6.66 (-15.38,2.06)	⊕◯◯◯ Very low
c-tDCS-C4+a-tDCS-arm vs Rt-nVNS					-6.66 (-14.53,1.20)	⊕◯◯◯ Very low
c-tDCS-C4+a-tDCS-arm vs hf-tONS-Oz					-6.79 (-18.19,4.62)	⊕◯◯◯ Very low
c-tDCS-C4+a-tDCS-arm vs af-tONS-Oz					-6.96 (-18.57,4.65)	⊕◯◯◯ Very low
c-tDCS-C4+a-tDCS-arm vs a-tDCS-C3+c-tDCS-Fp2					-7.87 (-16.46,0.72)	⊕◯◯◯ Very low
c-tDCS-C4+a-tDCS-arm vs Sham/Control	*-8.00 (-11.66,-4.34)	⊕⊕◯◯ Low			*-8.00 (-14.60,-1.40)	⊕⊕⊕◯ Medium
hf-TMS-F3 vs single-hf-TMS-F3	-1.35 (-9.90,7.20)	⊕◯◯◯ Very low	9.84 (14272.78)	⊕◯◯◯ Very low	-1.35 (-11.50,8.81)	⊕⊕⊕◯ Medium
hf-TMS-F3 vs a-tDCS-Oz+c-tDCS-Cz					-4.38 (-11.94,3.19)	⊕◯◯◯ Very low
hf-TMS-F3 vs c-tDCS-Oz+a-tCDS-Cz					-4.54 (-14.97,5.90)	⊕◯◯◯ Very low
hf-TMS-F3 vs taVNS					-4.48 (-12.20,3.24)	⊕◯◯◯ Very low
hf-TMS-F3 vs PENS-Fp1Fp2					-4.78 (-12.49,2.94)	⊕◯◯◯ Very low
hf-TMS-F3 vs lf-tONS-Oz					-5.06 (-15.28,5.16)	⊕◯◯◯ Very low
hf-TMS-F3 vs STS-Afz					-4.94 (-12.65,2.78)	⊕◯◯◯ Very low
hf-TMS-F3 vs Rt-nVNS					-4.94 (-11.52,1.64)	⊕◯◯◯ Very low
hf-TMS-F3 vs hf-tONS-Oz					-5.06 (-15.72,5.59)	⊕◯◯◯ Very low

hf-TMS-F3 vs af-tONS-Oz					-5.24 (-16.12,5.64)	⊕◯◯◯ Very low
hf-TMS-F3 vs a-tDCS-C3+c-tDCS-Fp2					-6.15 (-13.72,1.43)	⊕◯◯◯ Very low
hf-TMS-F3 vs Sham/Control	-6.81 (-13.86,0.24)	⊕◯◯◯ Very low	-0.64 (4378.44)	⊕◯◯◯ Very low	***-6.28 (-11.47,-1.08)**	⊕⊕⊕◯ Medium
single-hf-TMS-F3 vs a-tDCS-Oz+c-tDCS-Cz					-3.03 (-15.70,9.64)	⊕◯◯◯ Very low
single-hf-TMS-F3 vs c-tDCS-Oz+a-tCDS-Cz					-3.19 (-17.76,11.37)	⊕◯◯◯ Very low
single-hf-TMS-F3 vs taVNS					-3.13 (-15.89,9.63)	⊕◯◯◯ Very low
single-hf-TMS-F3 vs PENS-Fp1Fp2					-3.43 (-16.19,9.32)	⊕◯◯◯ Very low
single-hf-TMS-F3 vs lf-tONS-Oz					-3.72 (-18.13,10.69)	⊕◯◯◯ Very low
single-hf-TMS-F3 vs STS-Afz					-3.59 (-16.35,9.16)	⊕◯◯◯ Very low
single-hf-TMS-F3 vs Rt-nVNS					-3.59 (-15.70,8.51)	⊕◯◯◯ Very low
single-hf-TMS-F3 vs hf-tONS-Oz					-3.72 (-18.44,11.00)	⊕◯◯◯ Very low
single-hf-TMS-F3 vs af-tONS-Oz					-3.89 (-18.77,10.99)	⊕◯◯◯ Very low
single-hf-TMS-F3 vs a-tDCS-C3+c-tDCS-Fp2					-4.80 (-17.47,7.87)	⊕◯◯◯ Very low
single-hf-TMS-F3 vs Sham/Control					-4.93 (-16.34,6.48)	⊕◯◯◯ Very low
a-tDCS-Oz+c-tDCS-Cz vs c-tDCS-Oz+a-tCDS-Cz					-0.16 (-10.75,10.43)	⊕◯◯◯ Very low
a-tDCS-Oz+c-tDCS-Cz vs taVNS					-0.10 (-8.03,7.83)	⊕◯◯◯ Very low
a-tDCS-Oz+c-tDCS-Cz vs PENS-Fp1Fp2					-0.40 (-8.32,7.52)	⊕◯◯◯ Very low
a-tDCS-Oz+c-tDCS-Cz vs lf-tONS-Oz					-0.69 (-11.06,9.69)	⊕◯◯◯ Very low
a-tDCS-Oz+c-tDCS-Cz vs STS-Afz					-0.56 (-8.48,7.36)	⊕◯◯◯ Very low
a-tDCS-Oz+c-tDCS-Cz vs Rt-nVNS					-0.56 (-7.53,6.41)	⊕◯◯◯ Very low
a-tDCS-Oz+c-tDCS-Cz vs hf-tONS-Oz					-0.69 (-11.49,10.12)	⊕◯◯◯ Very low
a-tDCS-Oz+c-tDCS-Cz vs af-tONS-Oz					-0.86 (-11.88,10.17)	⊕◯◯◯ Very low
a-tDCS-Oz+c-tDCS-Cz vs a-tDCS-C3+c-tDCS-Fp2					-1.77 (-9.55,6.01)	⊕◯◯◯ Very low
a-tDCS-Oz+c-tDCS-Cz vs Sham/Control	***-1.90 (-2.27,-1.53)**	⊕⊕⊕◯ Medium			-1.90 (-7.40,3.60)	⊕⊕⊕◯ Medium
c-tDCS-Oz+a-tCDS-Cz vs taVNS					0.06 (-10.64,10.76)	⊕◯◯◯ Very low
c-tDCS-Oz+a-tCDS-Cz vs PENS-Fp1Fp2					-0.24 (-10.93,10.46)	⊕◯◯◯ Very low
c-tDCS-Oz+a-tCDS-Cz vs lf-tONS-Oz					-0.52 (-13.15,12.10)	⊕◯◯◯ Very low
c-tDCS-Oz+a-tCDS-Cz vs STS-Afz					-0.40 (-11.09,10.30)	⊕◯◯◯ Very low
c-tDCS-Oz+a-tCDS-Cz vs Rt-nVNS					-0.40 (-10.41,9.61)	⊕◯◯◯ Very low

c-tDCS-Oz+a-tCDS-Cz vs hf-tONS-Oz			-0.52 (-13.50,12.45)	⊕◯◯◯ Very low
c-tDCS-Oz+a-tCDS-Cz vs af-tONS-Oz			-0.70 (-13.86,12.46)	⊕◯◯◯ Very low
c-tDCS-Oz+a-tCDS-Cz vs a-tDCS-C3+c-tDCS-Fp2			-1.61 (-12.20,8.99)	⊕◯◯◯ Very low
c-tDCS-Oz+a-tCDS-Cz vs Sham/Control	-1.74 (-8.94,5.46)	⊕◯◯◯ Very low	-1.74 (-10.79,7.31)	⊕⊕◯◯ Low
taVNS vs PENS-Fp1Fp2			-0.30 (-8.37,7.77)	⊕◯◯◯ Very low
taVNS vs lf-tONS-Oz			-0.59 (-11.08,9.90)	⊕◯◯◯ Very low
taVNS vs STS-Afz			-0.46 (-8.53,7.61)	⊕◯◯◯ Very low
taVNS vs Rt-nVNS			-0.46 (-7.60,6.67)	⊕◯◯◯ Very low
taVNS vs hf-tONS-Oz			-0.59 (-11.50,10.33)	⊕◯◯◯ Very low
taVNS vs af-tONS-Oz			-0.76 (-11.89,10.37)	⊕◯◯◯ Very low
taVNS vs a-tDCS-C3+c-tDCS-Fp2			-1.67 (-9.60,6.26)	⊕◯◯◯ Very low
taVNS vs Sham/Control	***-1.80 (-3.38,-0.22)**	⊕⊕⊕◯ Medium	-1.80 (-7.51,3.91)	⊕⊕◯◯ Low
PENS-Fp1Fp2 vs lf-tONS-Oz			-0.29 (-10.77,10.20)	⊕◯◯◯ Very low
PENS-Fp1Fp2 vs STS-Afz			-0.16 (-8.22,7.90)	⊕◯◯◯ Very low
PENS-Fp1Fp2 vs Rt-nVNS			-0.16 (-7.29,6.97)	⊕◯◯◯ Very low
PENS-Fp1Fp2 vs hf-tONS-Oz			-0.29 (-11.19,10.62)	⊕◯◯◯ Very low
PENS-Fp1Fp2 vs af-tONS-Oz			-0.46 (-11.59,10.67)	⊕◯◯◯ Very low
PENS-Fp1Fp2 vs a-tDCS-C3+c-tDCS-Fp2			-1.37 (-9.30,6.56)	⊕◯◯◯ Very low
PENS-Fp1Fp2 vs Sham/Control	-1.50 (-3.05,0.05)	⊕⊕◯◯ Low	-1.50 (-7.20,4.20)	⊕⊕◯◯ Low
lf-tONS-Oz vs STS-Afz			0.13 (-10.36,10.61)	⊕◯◯◯ Very low
lf-tONS-Oz vs Rt-nVNS			0.12 (-9.66,9.91)	⊕◯◯◯ Very low
lf-tONS-Oz vs hf-tONS-Oz	0.00 (-7.72,7.72)	⊕◯◯◯ Very low	0.00 (-9.47,9.47)	⊕⊕◯◯ Low
lf-tONS-Oz vs af-tONS-Oz	-0.17 (-8.20,7.86)	⊕◯◯◯ Very low	-0.17 (-9.90,9.55)	⊕⊕◯◯ Low
lf-tONS-Oz vs a-tDCS-C3+c-tDCS-Fp2			-1.08 (-11.46,9.30)	⊕◯◯◯ Very low
lf-tONS-Oz vs Sham/Control	-1.21 (-8.10,5.67)	⊕◯◯◯ Very low	-1.21 (-10.01,7.59)	⊕⊕◯◯ Low
STS-Afz vs Rt-nVNS			-0.00 (-7.13,7.13)	⊕◯◯◯ Very low
STS-Afz vs hf-tONS-Oz			-0.13 (-11.03,10.78)	⊕◯◯◯ Very low
STS-Afz vs af-tONS-Oz			-0.30 (-11.43,10.83)	⊕◯◯◯ Very low
STS-Afz vs a-tDCS-C3+c-tDCS-Fp2			-1.21 (-9.14,6.72)	⊕◯◯◯ Very low

STS-Afz vs Sham/Control	-1.34 (-2.89,0.21)	⊕⊕◯◯ Low	-1.34 (-7.04,4.36)	⊕⊕◯◯ Low
Rt-nVNS vs hf-tONS-Oz			-0.12 (-10.36,10.12)	⊕◯◯◯ Very low
Rt-nVNS vs af-tONS-Oz			-0.30 (-10.77,10.17)	⊕◯◯◯ Very low
Rt-nVNS vs a-tDCS-C3+c-tDCS-Fp2			-1.21 (-8.18,5.77)	⊕◯◯◯ Very low
Rt-nVNS vs Sham/Control	-0.71 (-2.06,0.64)	⊕⊕◯◯ Low	-1.34 (-5.62,2.94)	⊕⊕◯◯ Low
hf-tONS-Oz vs af-tONS-Oz	-0.17 (-8.75,8.40)	⊕◯◯◯ Very low	-0.17 (-10.35,10.00)	⊕⊕◯◯ Low
hf-tONS-Oz vs a-tDCS-C3+c-tDCS-Fp2			-1.08 (-11.89,9.73)	⊕◯◯◯ Very low
hf-tONS-Oz vs Sham/Control	-1.21 (-8.72,6.30)	⊕◯◯◯ Very low	-1.21 (-10.51,8.09)	⊕⊕◯◯ Low
af-tONS-Oz vs a-tDCS-C3+c-tDCS-Fp2			-0.91 (-11.94,10.12)	⊕◯◯◯ Very low
af-tONS-Oz vs Sham/Control	-1.04 (-8.87,6.79)	⊕◯◯◯ Very low	-1.04 (-10.60,8.52)	⊕⊕◯◯ Low
a-tDCS-C3+c-tDCS-Fp2 vs Sham/Control	-0.13 (-0.63,0.37)	⊕⊕◯◯ Low	-0.13 (-5.64,5.38)	⊕⊕◯◯ Low

eTable 9B: Quality of evidence for primary outcome: 50% response rate

Comparisons	GRADE					
	Direct		Indirect		Network meta-analysis	
	Odds ratio (95% CI)	The final rating of direct evidence	Co-efficiency (Standard error)	The final rating of indirect evidence	Odds ratio (95% CI)	Overall quality of evidence
hf-tONS-Oz vs lf-tONS-Oz	1.13 (0.53,2.37)	⊕⊕◯◯ Low			1.13 (0.53,2.37)	⊕⊕⊕◯ Medium
hf-tONS-Oz vs af-tONS-Oz	1.13 (0.53,2.37)	⊕⊕◯◯ Low			1.13 (0.53,2.37)	⊕⊕⊕◯ Medium
hf-tONS-Oz vs STS-Afz					2.85 (0.31,26.37)	⊕◯◯◯ Very low
hf-tONS-Oz vs PENS-Fp1Fp2					3.00 (0.32,27.77)	⊕◯◯◯ Very low
hf-tONS-Oz vs hf-TMS-F3					3.73 (0.49,28.22)	⊕◯◯◯ Very low
hf-tONS-Oz vs a-tDCS-Oz+c-tDCS-Cz					4.13 (0.35,49.06)	⊕◯◯◯ Very low
hf-tONS-Oz vs single-hf-TMS-F3					4.26 (0.55,33.26)	⊕◯◯◯ Very low
hf-tONS-Oz vs sTMS-Oz					5.06 (0.66,38.91)	⊕◯◯◯ Very low
hf-tONS-Oz vs Bi-nVNS					5.05 (0.66,38.95)	⊕◯◯◯ Very low
hf-tONS-Oz vs Rt-nVNS					6.91 (0.93,51.46)	⊕◯◯◯ Very low
hf-tONS-Oz vs Sham/Control	***9.00 (1.24,65.16)**	⊕⊕◯◯ Low			***9.00 (1.24,65.16)**	⊕⊕⊕◯ Medium
lf-tONS-Oz vs af-tONS-Oz	1.00 (0.46,2.19)	⊕◯◯◯ Very low			1.00 (0.46,2.19)	⊕⊕⊕◯ Medium
lf-tONS-Oz vs STS-Afz					2.54 (0.27,23.72)	⊕◯◯◯ Very low
lf-tONS-Oz vs PENS-Fp1Fp2					2.67 (0.28,24.98)	⊕◯◯◯ Very low
lf-tONS-Oz vs hf-TMS-F3					3.31 (0.43,25.42)	⊕◯◯◯ Very low
lf-tONS-Oz vs a-tDCS-Oz+c-tDCS-Cz					3.67 (0.30,44.08)	⊕◯◯◯ Very low
lf-tONS-Oz vs single-hf-TMS-F3					3.79 (0.48,29.95)	⊕◯◯◯ Very low
lf-tONS-Oz vs sTMS-Oz					4.50 (0.58,35.04)	⊕◯◯◯ Very low
lf-tONS-Oz vs Bi-nVNS					4.49 (0.58,35.07)	⊕◯◯◯ Very low
lf-tONS-Oz vs Rt-nVNS					6.14 (0.81,46.35)	⊕◯◯◯ Very low
lf-tONS-Oz vs Sham/Control	***8.00 (1.09,58.71)**	⊕⊕◯◯ Low			***8.00 (1.09,58.71)**	⊕⊕⊕◯ Medium
af-tONS-Oz vs STS-Afz					2.54 (0.27,23.72)	⊕◯◯◯ Very low

af-tONS-Oz vs PENS-Fp1Fp2					2.67 (0.28,24.98)	⊕◯◯◯ Very low
af-tONS-Oz vs hf-TMS-F3					3.31 (0.43,25.42)	⊕◯◯◯ Very low
af-tONS-Oz vs a-tDCS-Oz+c-tDCS-Cz					3.67 (0.30,44.08)	⊕◯◯◯ Very low
af-tONS-Oz vs single-hf-TMS-F3					3.79 (0.48,29.95)	⊕◯◯◯ Very low
af-tONS-Oz vs sTMS-Oz					4.50 (0.58,35.04)	⊕◯◯◯ Very low
af-tONS-Oz vs Bi-nVNS					4.49 (0.58,35.07)	⊕◯◯◯ Very low
af-tONS-Oz vs Rt-nVNS					6.14 (0.81,46.35)	⊕◯◯◯ Very low
af-tONS-Oz vs Sham/Control	*8.00 (1.09,58.71)	⊕⊕◯◯ Low			*8.00 (1.09,58.71)	⊕⊕⊕◯ Medium
STS-Afz vs PENS-Fp1Fp2					1.05 (0.25,4.42)	⊕⊕◯◯ Low
STS-Afz vs hf-TMS-F3					1.31 (0.44,3.92)	⊕◯◯◯ Very low
STS-Afz vs a-tDCS-Oz+c-tDCS-Cz					1.45 (0.24,8.74)	⊕⊕◯◯ Low
STS-Afz vs single-hf-TMS-F3					1.49 (0.47,4.73)	⊕⊕◯◯ Low
STS-Afz vs sTMS-Oz					1.77 (0.58,5.47)	⊕⊕◯◯ Low
STS-Afz vs Bi-nVNS					1.77 (0.57,5.48)	⊕⊕◯◯ Low
STS-Afz vs Rt-nVNS					2.42 (0.83,7.04)	⊕⊕◯◯ Low
STS-Afz vs Sham/Control	*3.15 (1.15,8.69)	⊕⊕◯◯ Low			*3.15 (1.15,8.69)	⊕⊕⊕◯ Medium
PENS-Fp1Fp2 vs hf-TMS-F3					1.24 (0.41,3.73)	⊕⊕◯◯ Low
PENS-Fp1Fp2 vs a-tDCS-Oz+c-tDCS-Cz					1.38 (0.23,8.33)	⊕⊕◯◯ Low
PENS-Fp1Fp2 vs single-hf-TMS-F3					1.42 (0.45,4.51)	⊕⊕◯◯ Low
PENS-Fp1Fp2 vs sTMS-Oz					1.69 (0.55,5.21)	⊕⊕◯◯ Low
PENS-Fp1Fp2 vs Bi-nVNS					1.68 (0.54,5.23)	⊕⊕◯◯ Low
PENS-Fp1Fp2 vs Rt-nVNS					2.30 (0.79,6.72)	⊕⊕◯◯ Low
PENS-Fp1Fp2 vs Sham/Control	*3.00 (1.09,8.29)	⊕⊕◯◯ Low			*3.00 (1.09,8.29)	⊕⊕⊕◯ Medium
hf-TMS-F3 vs a-tDCS-Oz+c-tDCS-Cz					1.11 (0.24,5.19)	⊕⊕◯◯ Low
hf-TMS-F3 vs single-hf-TMS-F3	1.14 (0.80,1.63)	⊕⊕◯◯ Low	1.00 (0.00004)	⊕⊕⊕◯ Medium	1.14 (0.80,1.63)	⊕⊕⊕⊕ High
hf-TMS-F3 vs sTMS-Oz					1.36 (0.71,2.59)	⊕⊕◯◯ Low
hf-TMS-F3 vs Bi-nVNS					1.36 (0.70,2.61)	⊕⊕◯◯ Low
hf-TMS-F3 vs Rt-nVNS					*1.85 (1.08,3.18)	⊕⊕⊕◯ Medium
hf-TMS-F3 vs Sham/Control	*2.41 (1.58,3.68)	⊕⊕⊕◯ Medium	1.00 (0.00004)	⊕⊕⊕◯ Medium	*2.41 (1.58,3.68)	⊕⊕⊕⊕ High

a-tDCS-Oz+c-tDCS-Cz vs single-hf-TMS-F3			1.03 (0.21,5.05)	⊕⊕◯◯ Low
a-tDCS-Oz+c-tDCS-Cz vs sTMS-Oz			1.23 (0.26,5.87)	⊕⊕◯◯ Low
a-tDCS-Oz+c-tDCS-Cz vs Bi-nVNS			1.23 (0.26,5.88)	⊕⊕◯◯ Low
a-tDCS-Oz+c-tDCS-Cz vs Rt-nVNS			1.67 (0.36,7.70)	⊕⊕◯◯ Low
a-tDCS-Oz+c-tDCS-Cz vs Sham/Control	2.18 (0.49,9.65)	⊕◯◯◯ Very low	2.18 (0.49,9.65)	⊕⊕⊕◯ Medium
single-hf-TMS-F3 vs sTMS-Oz			1.19 (0.57,2.48)	⊕⊕◯◯ Low
single-hf-TMS-F3 vs Bi-nVNS			1.19 (0.56,2.50)	⊕⊕◯◯ Low
single-hf-TMS-F3 vs Rt-nVNS			1.62 (0.85,3.10)	⊕⊕◯◯ Low
single-hf-TMS-F3 vs Sham/Control			***2.11 (1.22,3.67)**	⊕⊕⊕◯ Medium
sTMS-Oz vs Bi-nVNS			1.00 (0.50,2.01)	⊕⊕◯◯ Low
sTMS-Oz vs Rt-nVNS			1.36 (0.75,2.47)	⊕⊕◯◯ Low
sTMS-Oz vs Sham/Control	***1.78 (1.09,2.90)**	⊕⊕⊕◯ Medium	***1.78 (1.09,2.90)**	⊕⊕⊕◯ Medium
Bi-nVNS vs Rt-nVNS			1.37 (0.75,2.50)	⊕⊕◯◯ Low
Bi-nVNS vs Sham/Control	***1.78 (1.08,2.94)**	⊕⊕⊕◯ Medium	***1.78 (1.08,2.94)**	⊕⊕⊕◯ Medium
Rt-nVNS vs Sham/Control	1.30 (0.93,1.83)	⊕◯◯◯ Very low	1.30 (0.93,1.83)	⊕⊕◯◯ Low

We evaluated the GRADE ratings according to the rationale of the articles published in the BMJ [1] and the Lancet [2]

Abbreviation: 95%CI: 95% confidence interval; af-tONS-Oz: alternating frequency tONS over Oz; a-tDCS-C3+c-tDCS-Fp2: anode tDCS over C3 + cathode tDCS over Fp2; a-tDCS-F3+c-tDCS-Fp2: anode tDCS over F3 + cathode tDCS over Fp2; a-tDCS-Oz+c-tDCS-Cz: anode tDCS over Oz + cathode over Cz; Bi-nVNS: bilateral vagus nerve stimulation; c-tDCS-C4+a-tDCS-arm: cathode tDCS over C4 + anode at left upper arm; c-tDCS-CP4+a-tDCS-arm: cathode tDCS over CP4 + anode at left upper arm; c-tDCS-Oz+a-tCDS-Cz: cathode tDCS over Oz + anode tCDS over Cz; dTMS-F3: deep TMS-F3; ES: effect size; hf-TMS-C3: high frequency rTMS over C3; hf-TMS-F3: high frequency rTMS over F3; hf-tONS-Oz: high frequency tONS over Oz; lf-tONS-Oz: low frequency tONS over Oz; MD: mean difference; NMA: network meta-analysis; nVNS: noninvasive vagus nerve stimulation; PENS: percutaneous electrical nerve stimulation; PENS-Fp1Fp2: percutaneous electrical nerve stimulation over Fp1Fp2; RCT: randomized controlled trial; RR: rate ratio; rTMS: repetitive transcranial magnetic stimulation; Rt-nVNS: right vagus nerve stimulation; Sham/Control: Sham control or waiting list; single-hf-TMS-F3: single session high frequency rTMS over F3; SMD: standardized mean difference; sTMS: single-pulse TMS; sTMS-Oz: single-pulse TMS over Oz; STS: supraorbital transcutaneous stimulation; STS-Afz: supraorbital transcutaneous stimulator over Afz; SUCRA: surface under the cumulative ranking curve; taVNS: transcutaneous auricular vagus nerve stimulation; tDCS: transcranial direct current stimulation; TMS: transcranial magnetic stimulation; tONS: transcutaneous occipital nerve stimulation

References:

[1] Puhan MA, Schunemann HJ, Murad MH, Li T, Brignardello-Petersen R, Singh JA, et al. A GRADE Working Group approach for rating the quality of treatment effect estimates from network meta-analysis. Bmj 2014;349:g5630.

[2] Cipriani A, Furukawa TA, Salanti G, Chaimani A, Atkinson LZ, Ogawa Y, et al. Comparative efficacy and acceptability of 21 antidepressant drugs for the acute treatment of adults with major depressive disorder: a systematic review and network meta-analysis. Lancet 2018.
