# Supplementary material for: Effectiveness and acceptability of noninvasive brain and nerve stimulation techniques for migraine prophylaxis: a network meta-analysis of randomized controlled trials
Source: J Headache Pain. 2022 Feb 20;23(1):28. doi: 10.1186/s10194-022-01401-3 (PMC8903676; doi:10.1186/s10194-022-01401-3)
Supplement: Supplementary file 2 — Additional file 2. [file 10194_2022_1401_MOESM2_ESM.pdf]

Intervention	Study name	SMD	Lower limit	Upper limit	*p* value	Relative weight
		Statistics for each study				
nVNS	Diener, H.C. (2019)	-0.515	-0.677	-0.354	0.000	50.50
nVNS	Zhang, Y. (2021)	-0.207	-0.595	0.181	0.296	28.81
nVNS	Silberstein, S.D. (2016)	-0.049	-0.564	0.466	0.853	20.69
**nVNS**	**nVNS overall**	**-0.330**	**-0.619**	**-0.040**	**0.025**	**100.00**
rTMS	Misra, U.K. (2013)	-0.932	-1.349	-0.515	0.000	41.95
rTMS	Brighina, F. (2004)	-0.805	-2.094	0.484	0.221	25.19
rTMS	Kumar, A. (2021)	0.502	-0.388	1.392	0.269	32.86
**rTMS**	**rTMS overall**	**-0.429**	**-1.397**	**0.539**	**0.385**	**100.00**
tDCS	Rahimi, M.D. (2020)	-0.269	-0.988	0.450	0.463	22.63
tDCS	Antal, A. (2011)	-0.155	-0.924	0.615	0.694	19.84
tDCS	Auvichayapat, P. (2012)	0.136	-0.537	0.809	0.691	25.66
tDCS	Pohl, H. (2020)	0.500	-0.100	1.100	0.102	31.87
**tDCS**	**tDCS overall**	**0.103**	**-0.247**	**0.452**	**0.565**	**100.00**

Interaction test *p* value = 0.151

SMD and 95%CIs

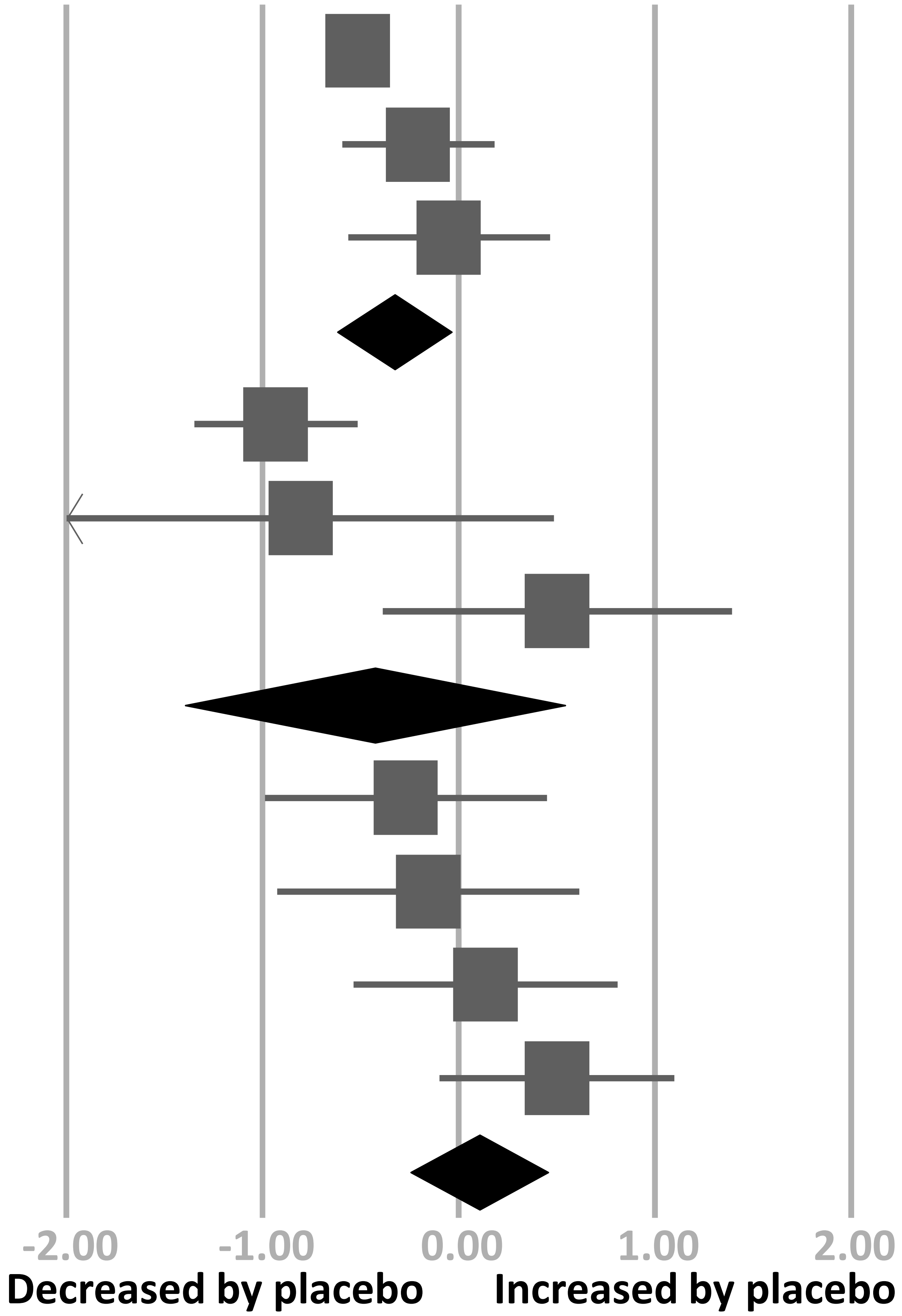

eFigure 1A: Test for transitivity assumption: Changes in monthly migraine days

Intervention	Study name	Event rate	Lower limit	Upper limit	Relative weight
		**Statistics for each study**			
nVNS	Silberstein, S.D. (2016)	0.033	0.005	0.202	8.85
nVNS	Tassorelli C (2018)	0.182	0.118	0.270	42.13
nVNS	Diener, H.C. (2019)	0.251	0.192	0.323	49.02
**nVNS**	**nVNS overall**	**0.188**	**0.108**	**0.305**	**100.00**
rTMS	Misra, U.K. (2013)	0.333	0.215	0.477	85.92
rTMS	Brighina, F. (2004)	0.083	0.005	0.622	14.08
**rTMS**	**rTMS overall**	**0.282**	**0.110**	**0.557**	**100.00**

Event rate and 95%CIs

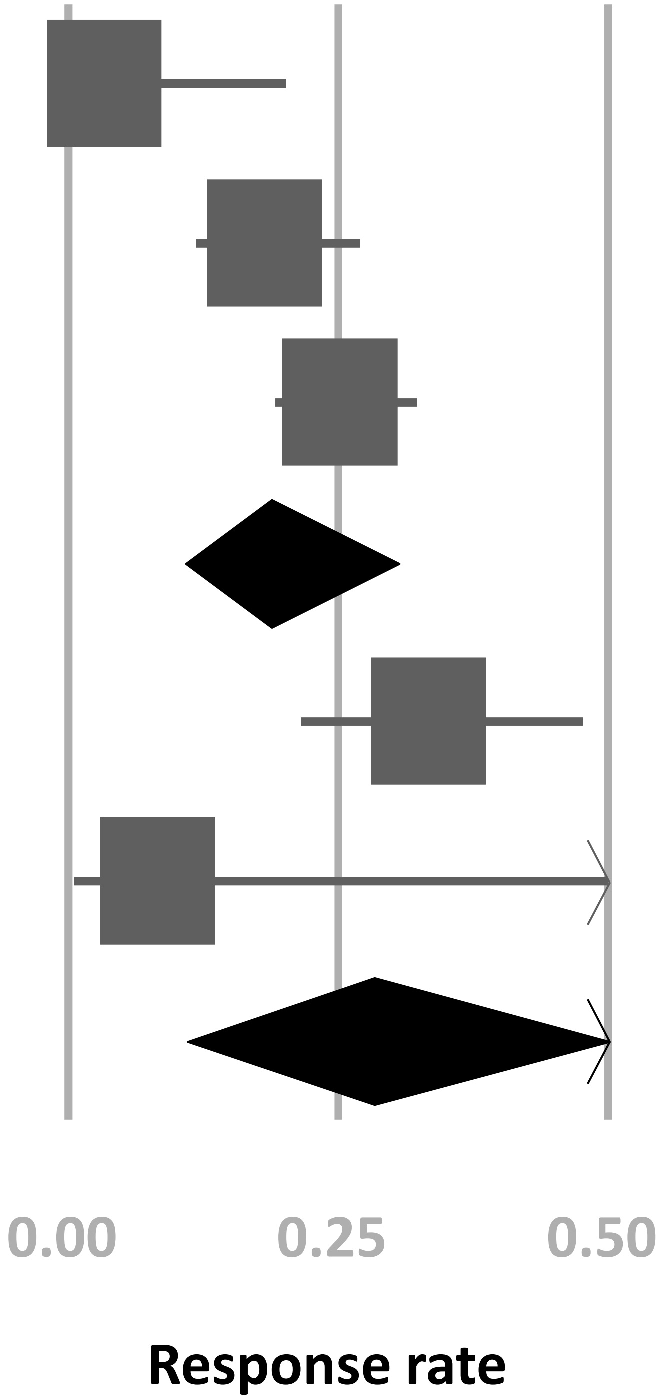

Interaction test *p* value = 0.432

eFigure 1B: Test for transitivity assumption: Response rate

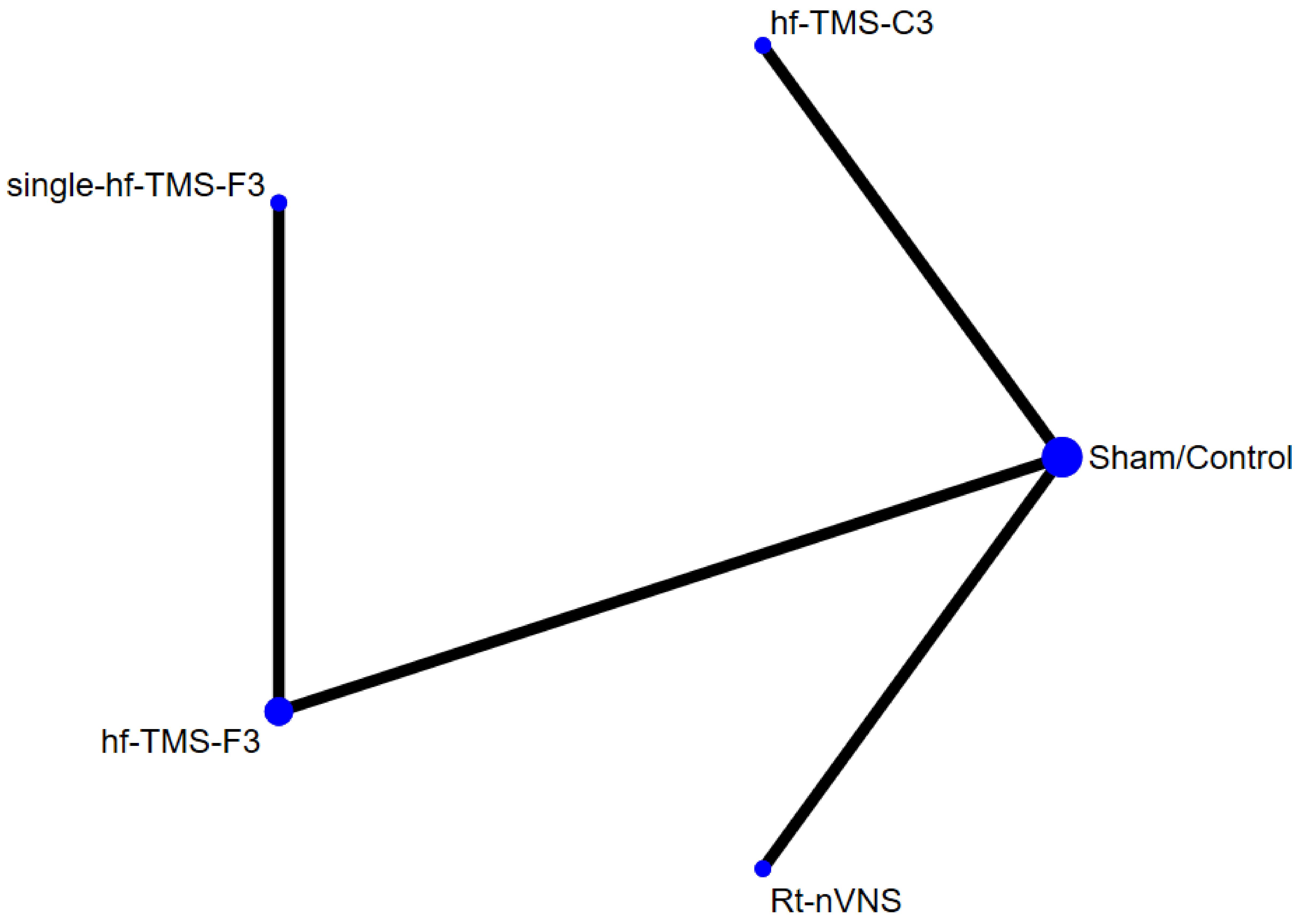

eFigure 2A Network structure of changes in monthly migraine days: chronic migraine

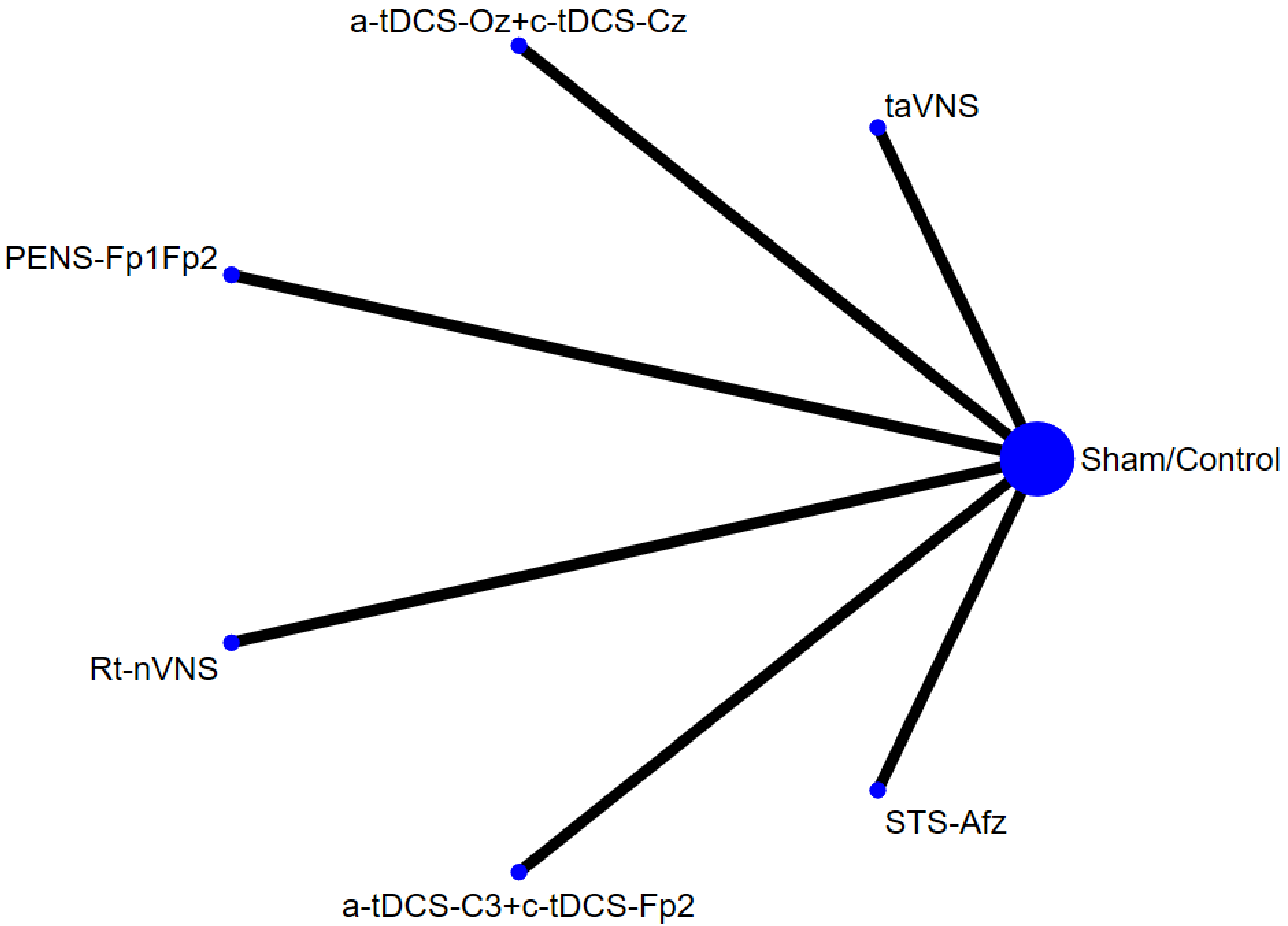

eFigure 2B Network structure of changes in monthly migraine days: episodic migraine

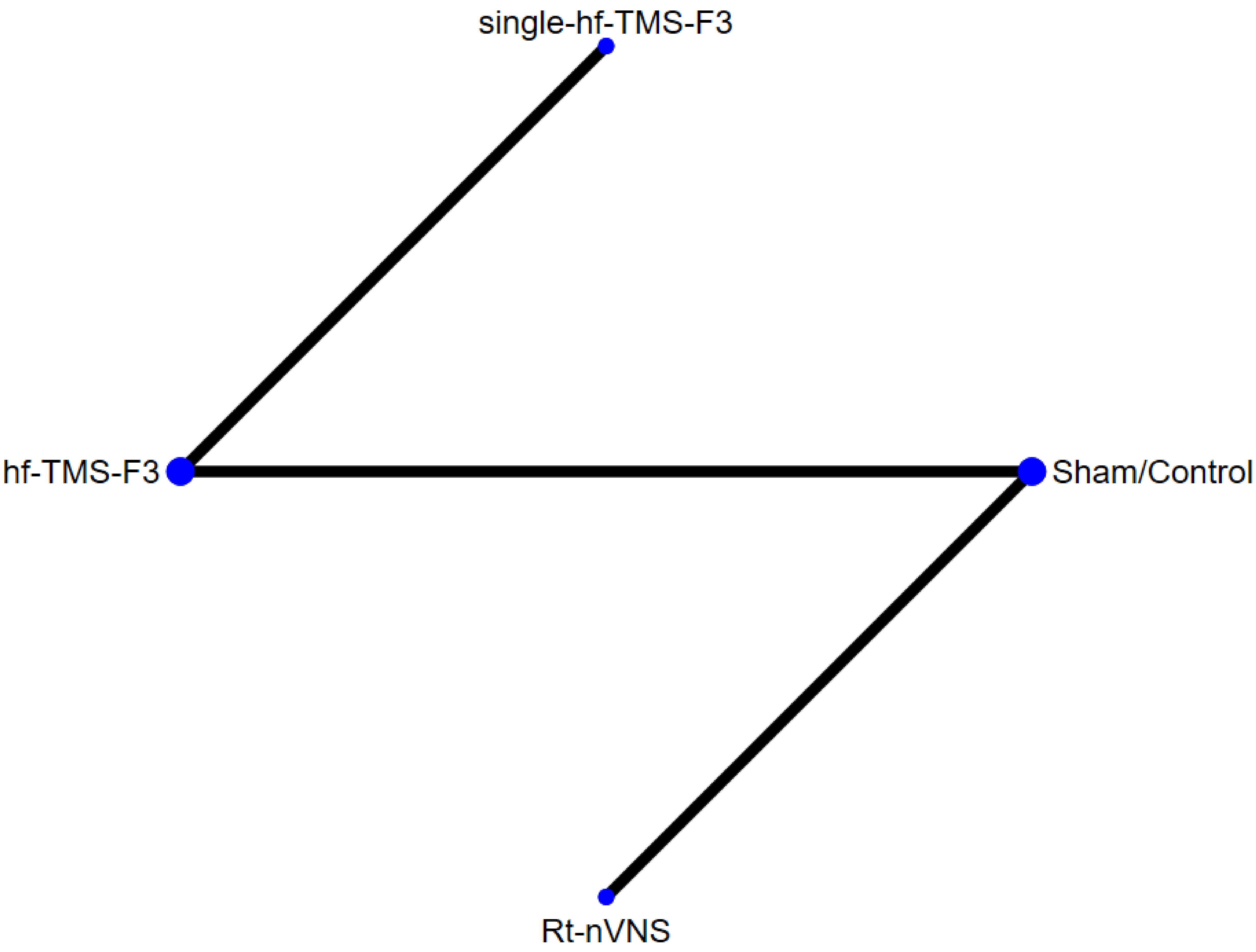

eFigure 2C Network structure of response rate: chronic migraine

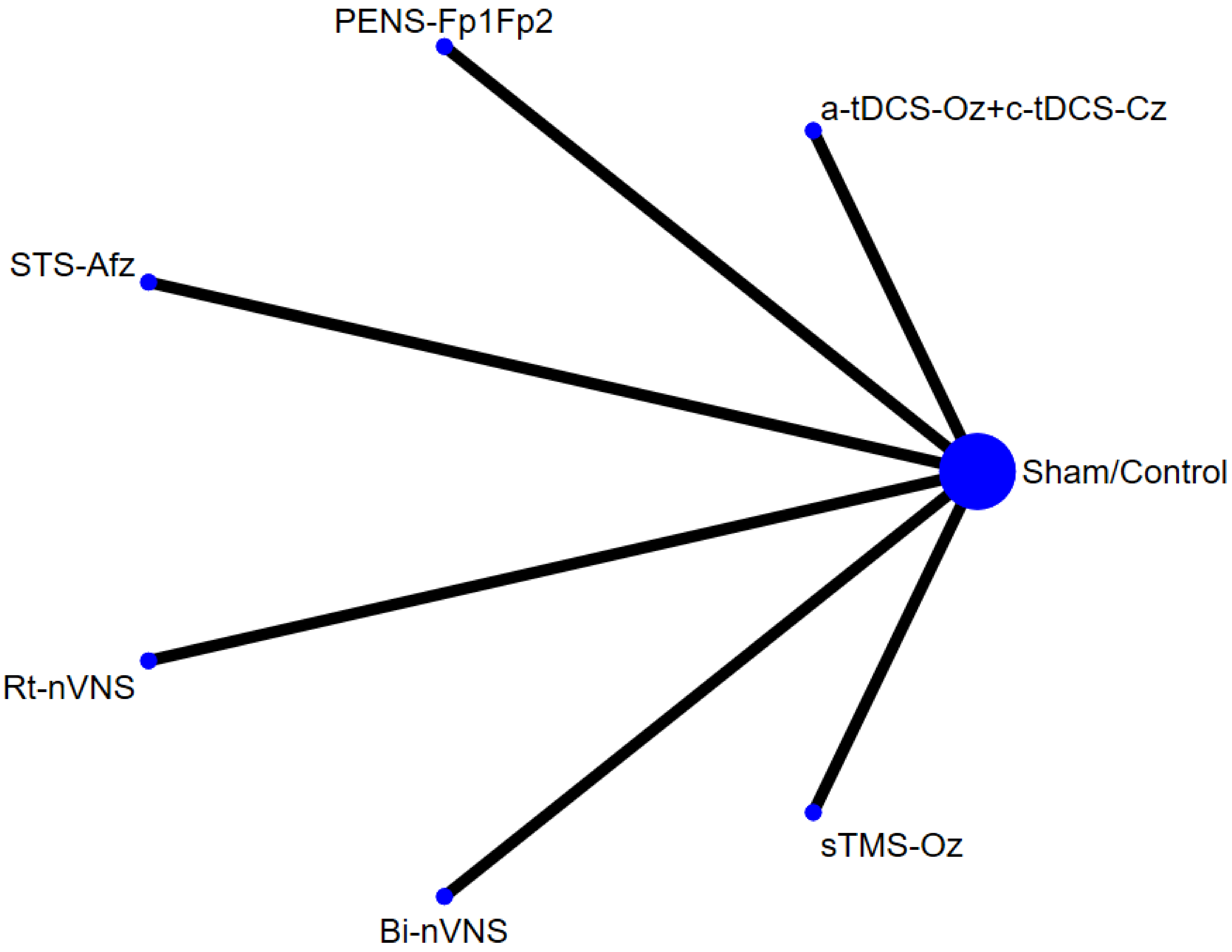

eFigure 2D Network structure of response rate: episodic migraine

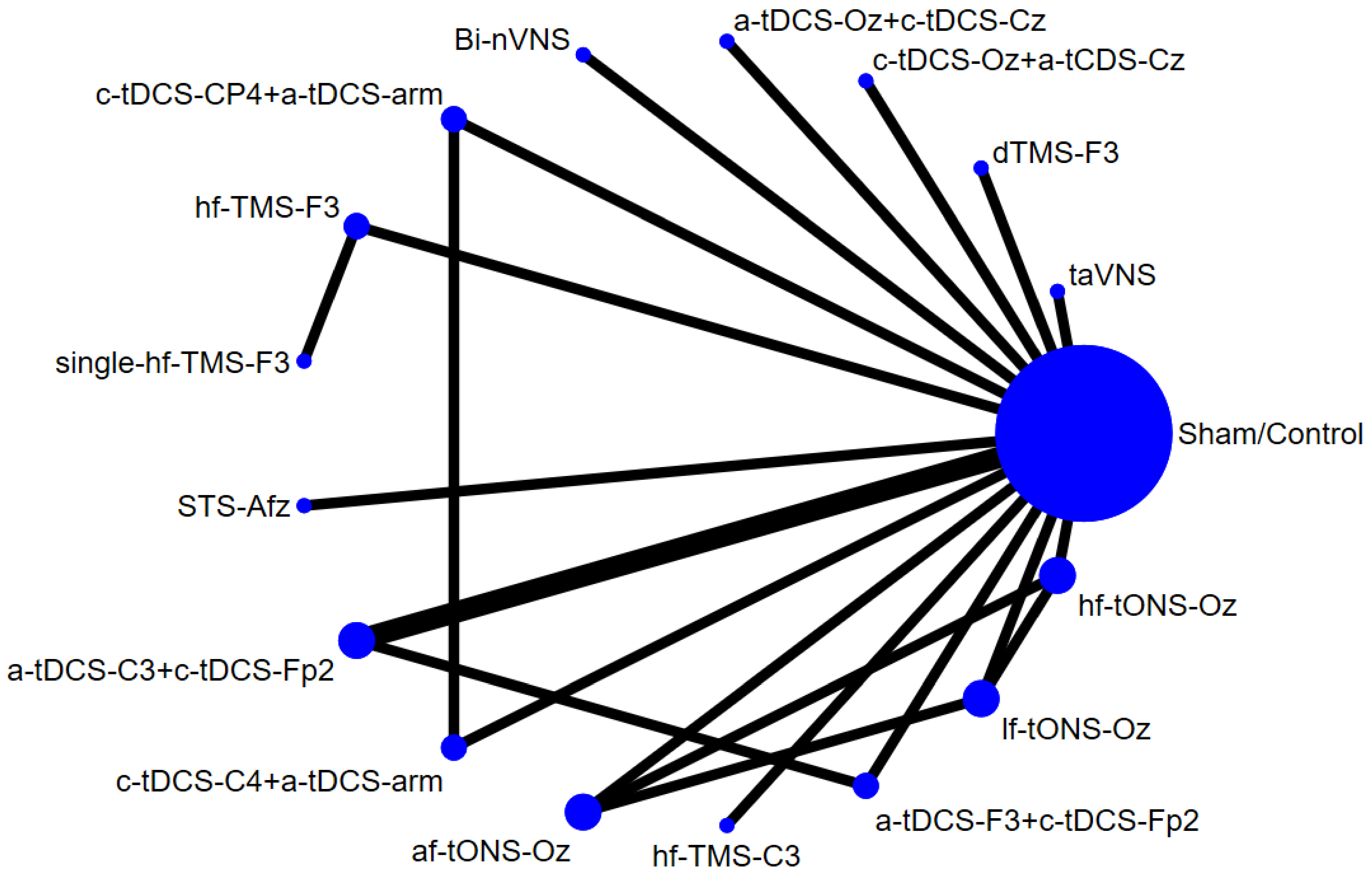

eFigure 2E Network structure of migraine pain severity

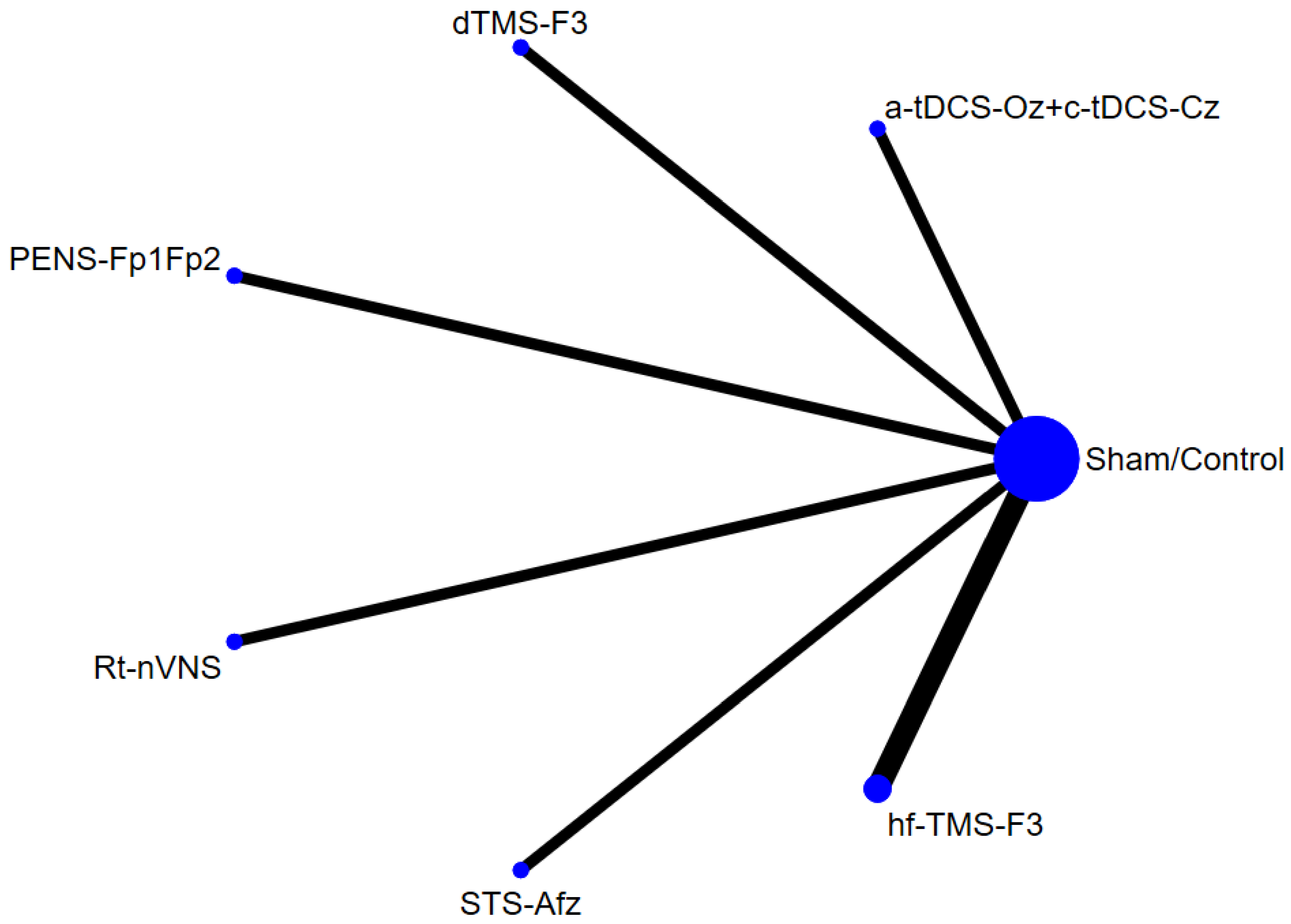

eFigure 2F Network structure of rescue medication

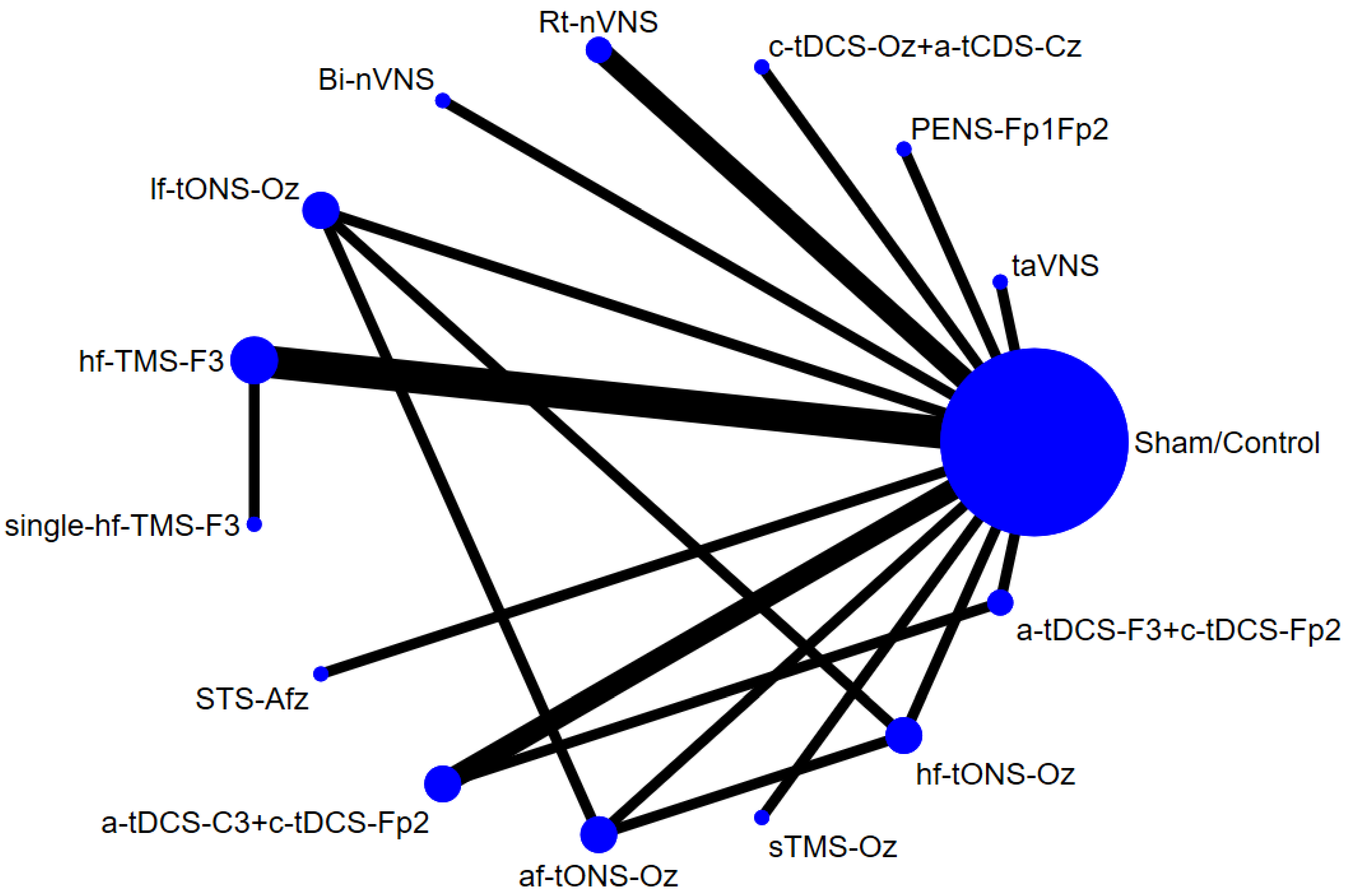

eFigure 2G Network structure of drop-out rate

Figure legend of eFigure 2A-2G

The lines between nodes represent direct comparisons in various trials, and the size of each circle is proportional to the size of the population involved in each specific treatment. The thickness of the lines is proportional to the number of trials connected to the network.

Abbreviation: 95%CI: 95% confidence interval; af-tONS-Oz: alternating frequency tONS over Oz; a-tDCS-C3+c-tDCS-Fp2: anode tDCS over C3 + cathode tDCS over Fp2; a-tDCS-F3+c-tDCS-Fp2: anode tDCS over F3 + cathode tDCS over Fp2; a-tDCS-Oz+c-tDCS-Cz: anode tDCS over Oz + cathode over Cz; Bi-nVNS: bilateral vagus nerve stimulation; c-tDCS-C4+a-tDCS-arm: cathode tDCS over C4 + anode at left upper arm; c-tDCS-CP4+a-tDCS-arm: cathode tDCS over CP4 + anode at left upper arm; c-tDCS-Oz+a-tCDS-Cz: cathode tDCS over Oz + anode tCDS over Cz; dTMS-F3: deep TMS-F3; ES: effect size; hf-TMS-C3: high frequency rTMS over C3; hf-TMS-F3: high frequency rTMS over F3; hf-tONS-Oz: high frequency tONS over Oz; lf-tONS-Oz: low frequency tONS over Oz; MD: mean difference; NMA: network meta-analysis; nVNS: noninvasive vagus nerve stimulation; PENS: percutaneous electrical nerve stimulation; PENS-Fp1Fp2: percutaneous electrical nerve stimulation over Fp1Fp2; RCT: randomized controlled trial; RR: rate ratio; rTMS: repetitive transcranial magnetic stimulation; Rt-nVNS: right vagus nerve stimulation; Sham/Control: Sham control or waiting list; single-hf-TMS-F3: single session high frequency rTMS over F3; SMD: standardized mean difference; sTMS: single-pulse TMS; sTMS-Oz: single-pulse TMS over Oz; STS: supraorbital transcutaneous stimulation; STS-Afz: supraorbital transcutaneous stimulator over Afz; SUCRA: surface under the cumulative ranking curve; taVNS: transcutaneous auricular vagus nerve stimulation; tDCS: transcranial direct current stimulation; TMS: transcranial magnetic stimulation; tONS: transcutaneous occipital nerve stimulation

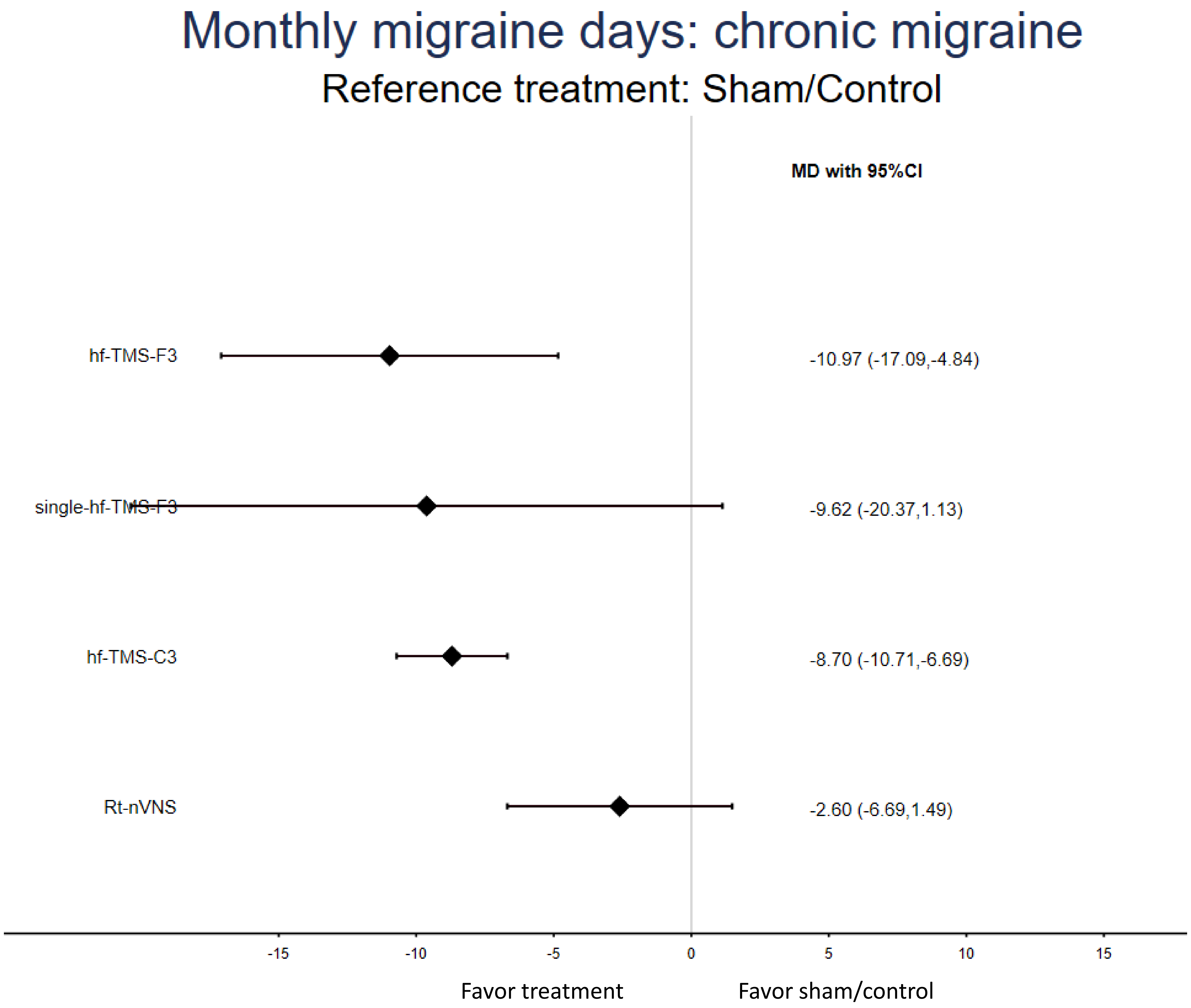

eFigure 3A forest plot of NMA of changes in monthly migraine days: chronic migraine

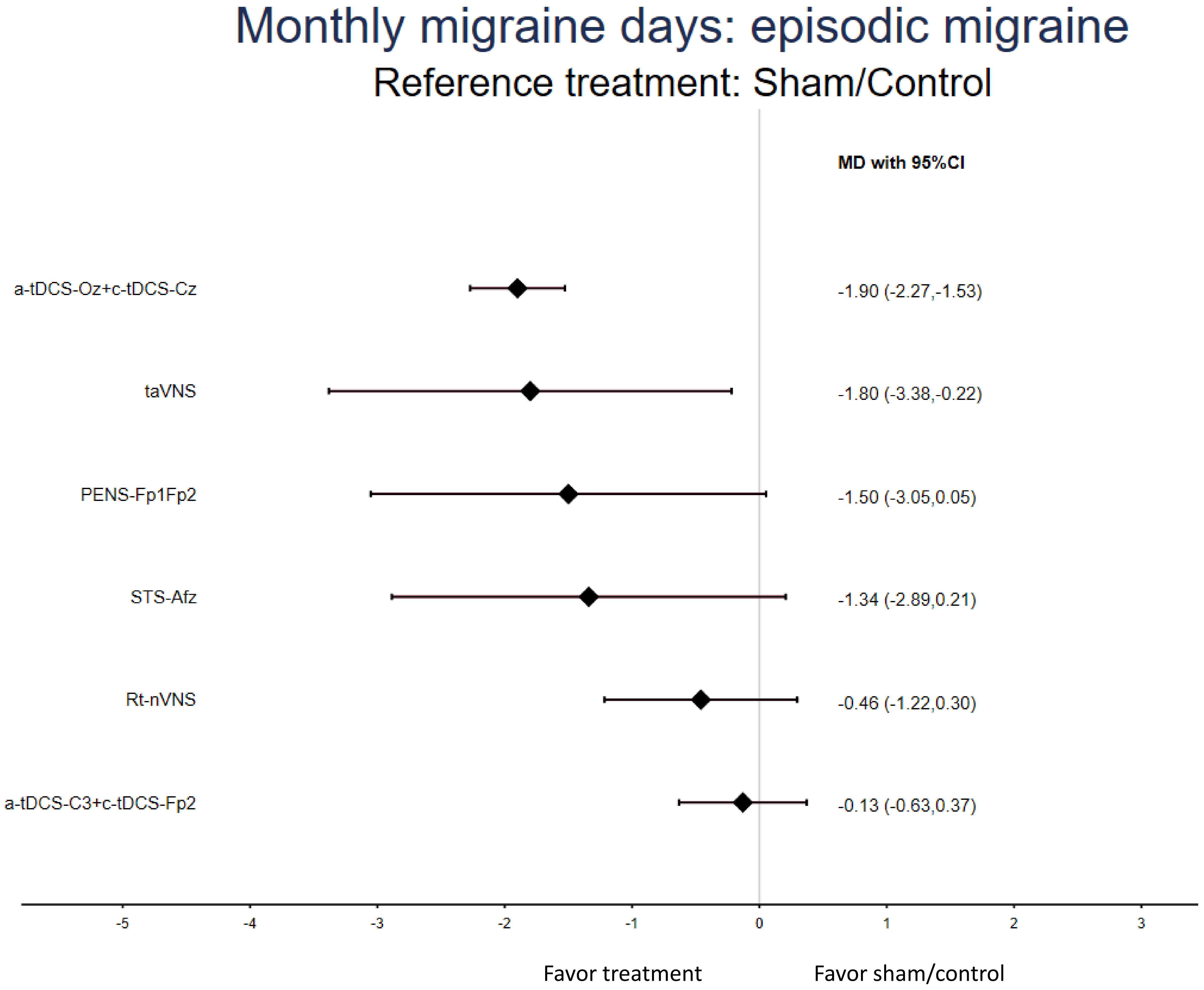

eFigure 3B forest plot of NMA of changes in monthly migraine days: episodic migraine

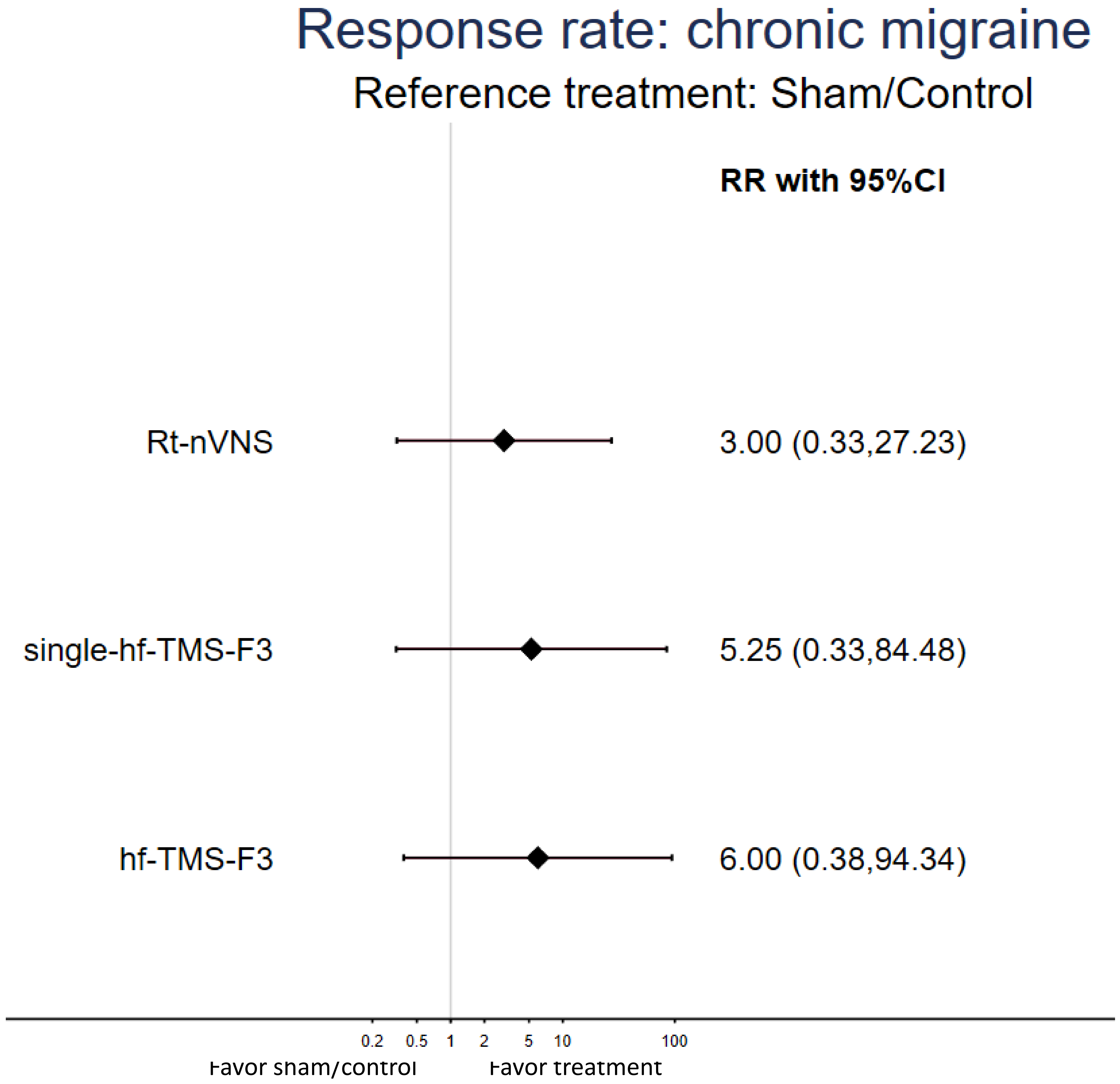

eFigure 3C forest plot of NMA of response rate: chronic migraine

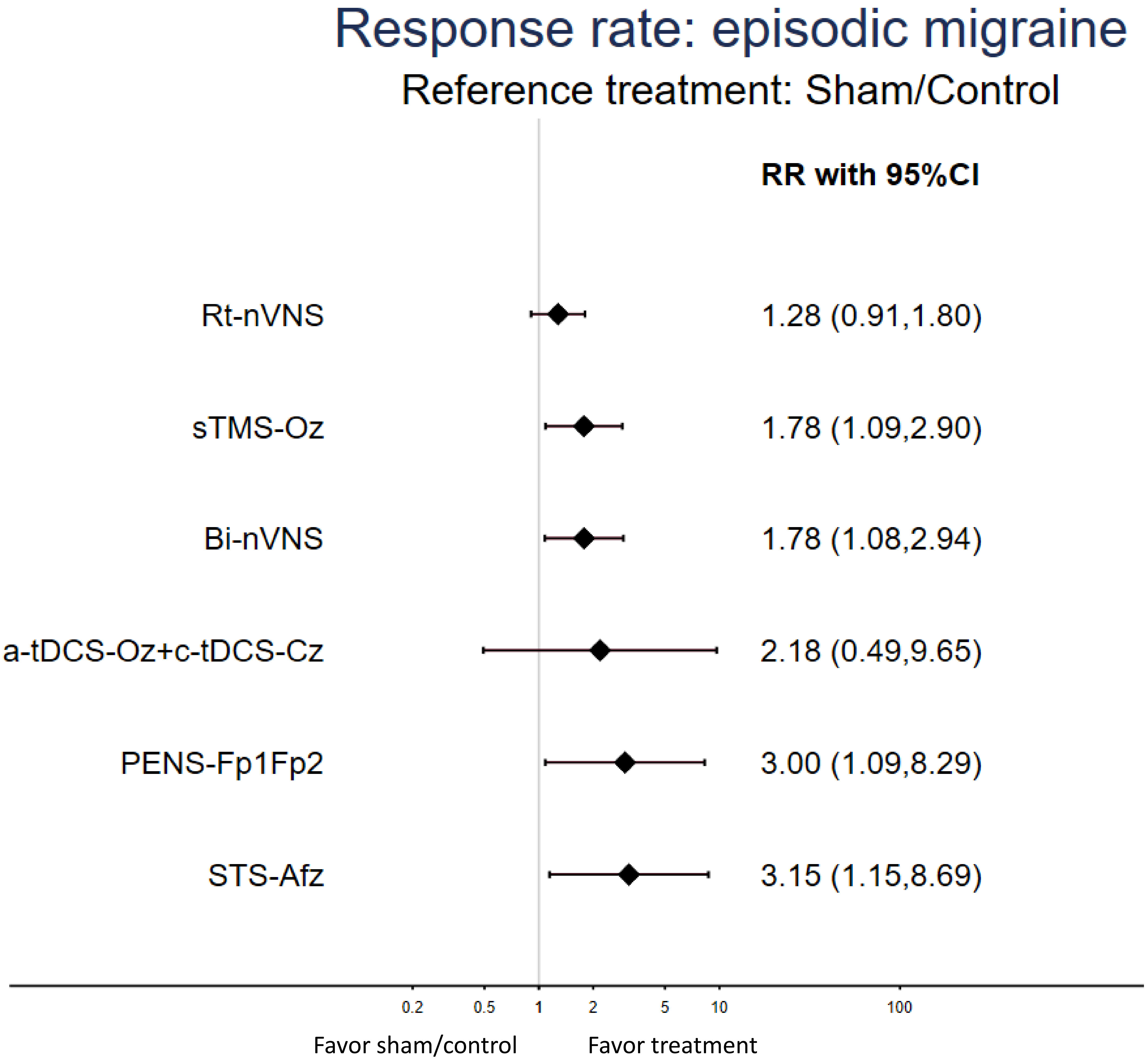

eFigure 3D forest plot of NMA of response rate: episodic migraine

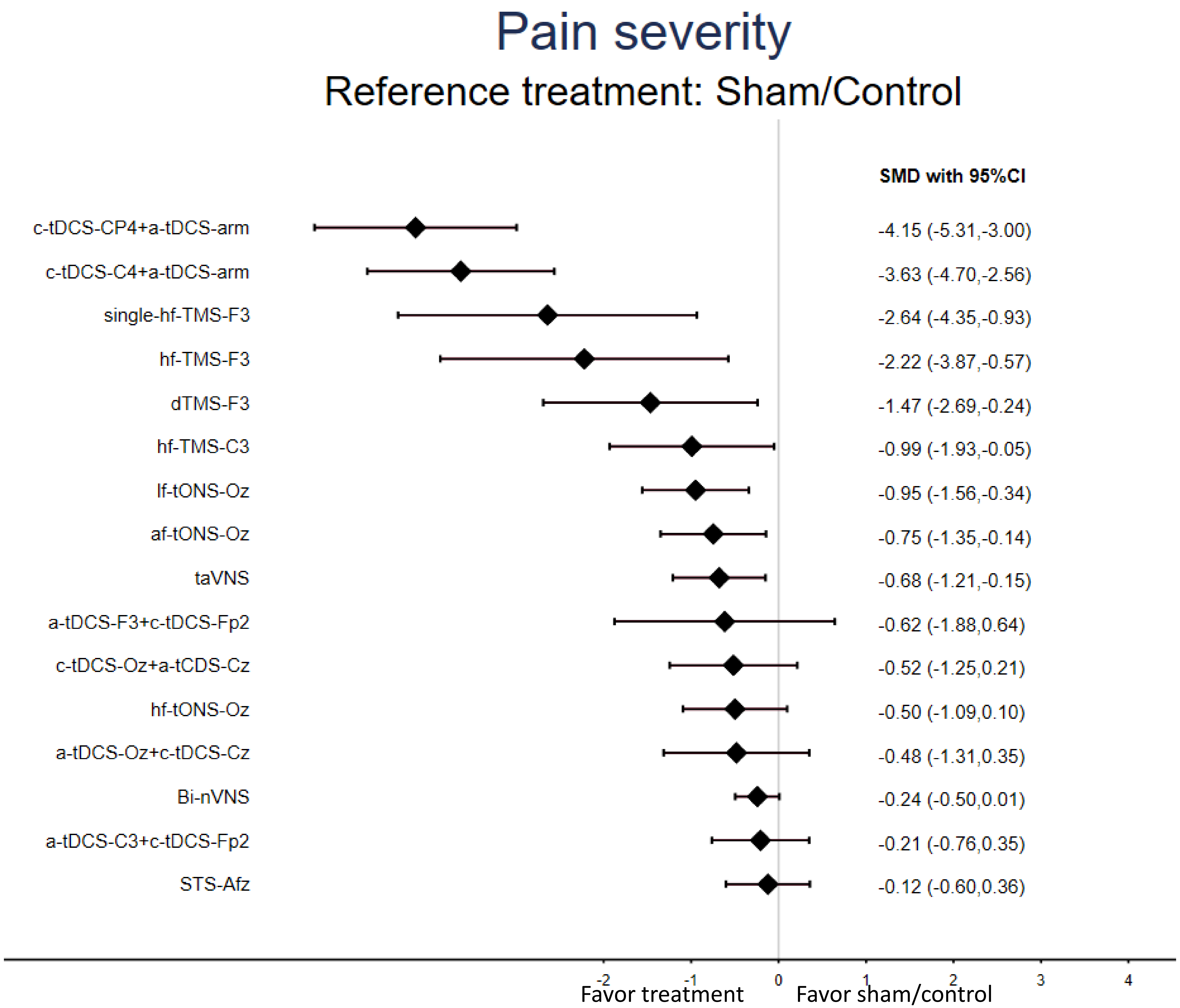

eFigure 3E forest plot of NMA of migraine pain severity

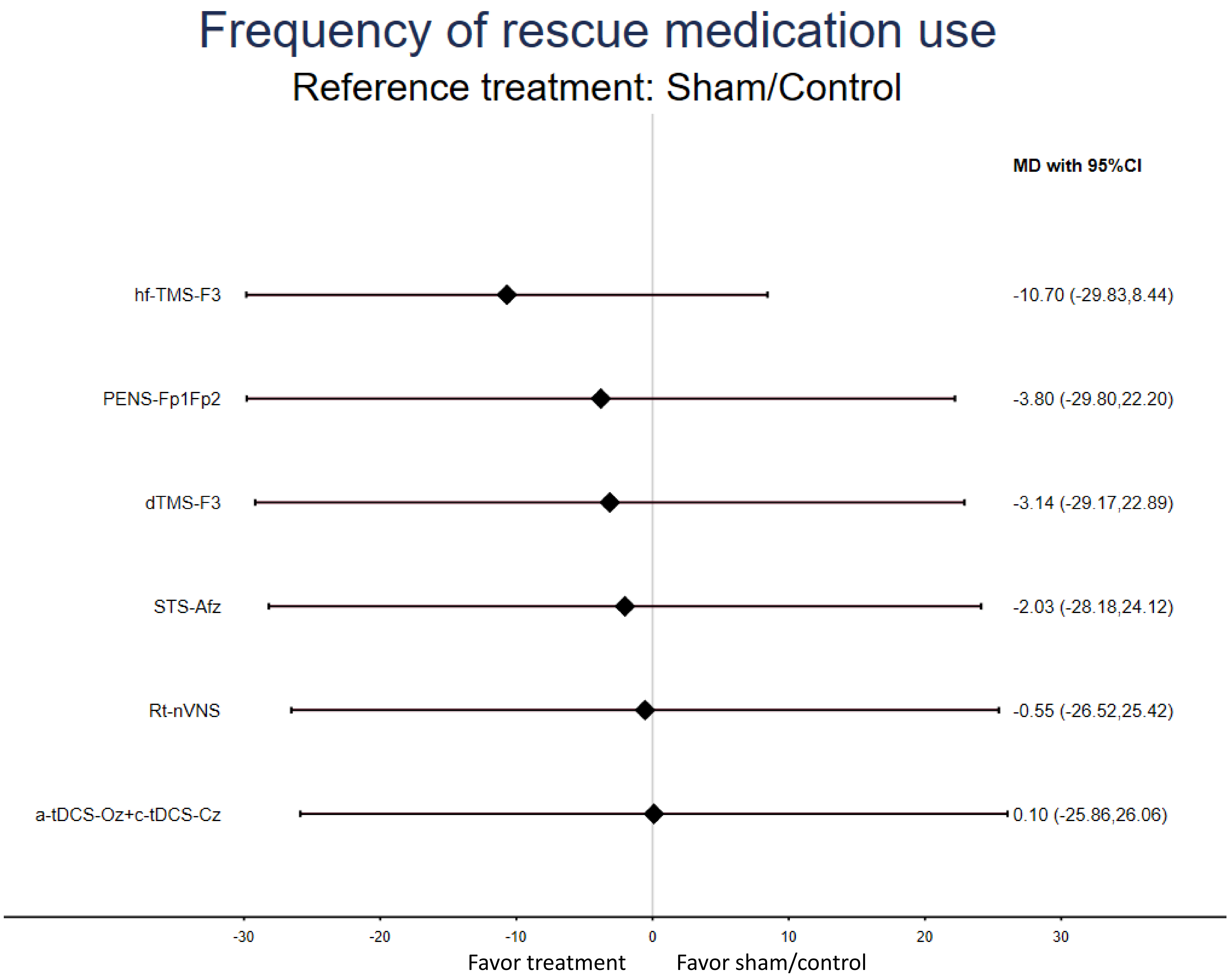

eFigure 3F forest plot of NMA of rescue medication

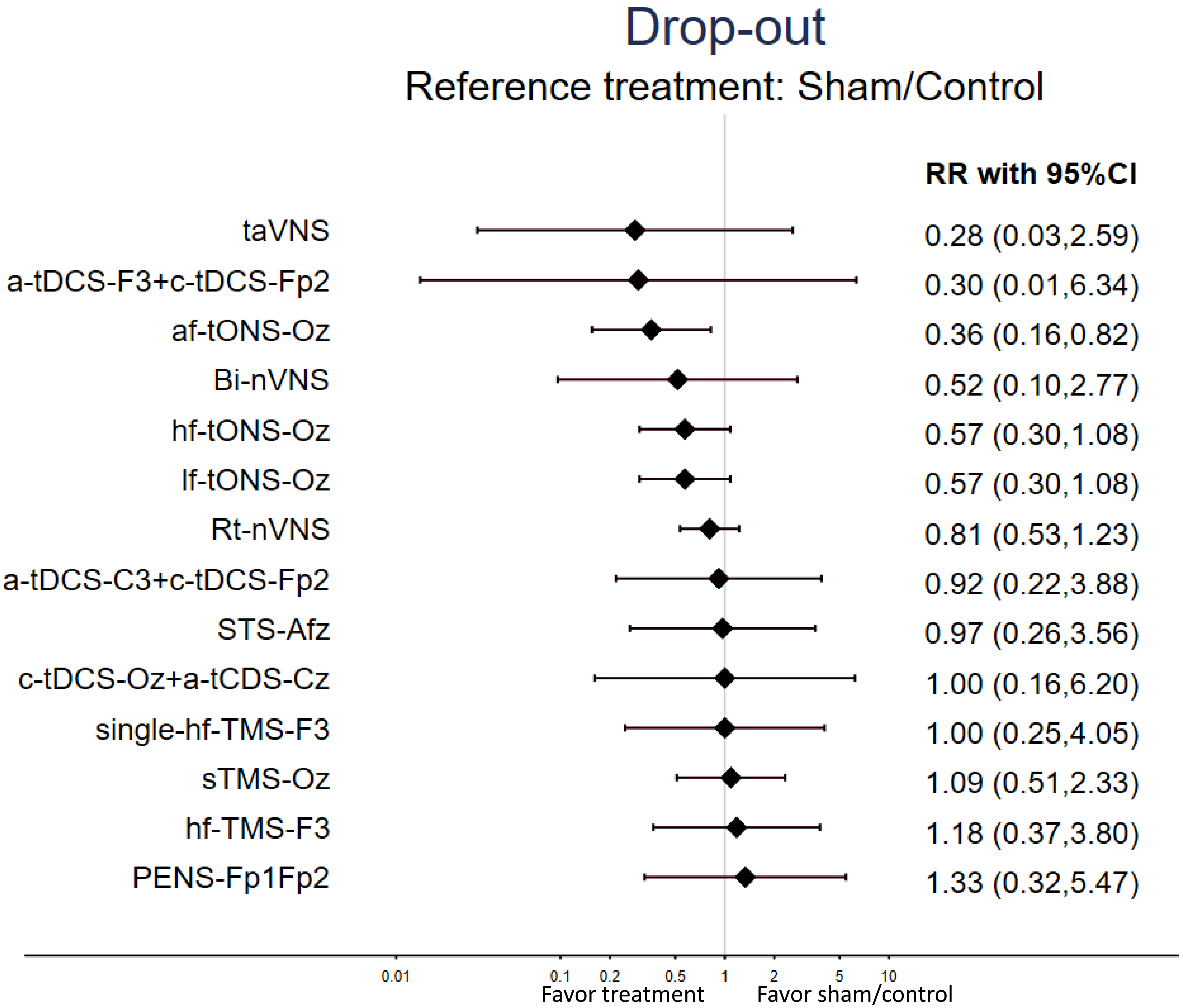

eFigure 3G forest plot of NMA of drop-out rate

Figure legend of eFigure 3A-3G

Abbreviation: 95%CI: 95% confidence interval; af-tONS-Oz: alternating frequency tONS over Oz; a-tDCS-C3+c-tDCS-Fp2: anode tDCS over C3 + cathode tDCS over Fp2; a-tDCS-F3+c-tDCS-Fp2: anode tDCS over F3 + cathode tDCS over Fp2; a-tDCS-Oz+c-tDCS-Cz: anode tDCS over Oz + cathode over Cz; Bi-nVNS: bilateral vagus nerve stimulation; c-tDCS-C4+a-tDCS-arm: cathode tDCS over C4 + anode at left upper arm; c-tDCS-CP4+a-tDCS-arm: cathode tDCS over CP4 + anode at left upper arm; c-tDCS-Oz+a-tCDS-Cz: cathode tDCS over Oz + anode tCDS over Cz; dTMS-F3: deep TMS-F3; ES: effect size; hf-TMS-C3: high frequency rTMS over C3; hf-TMS-F3: high frequency rTMS over F3; hf-tONS-Oz: high frequency tONS over Oz; lf-tONS-Oz: low frequency tONS over Oz; MD: mean difference; NMA: network meta-analysis; nVNS: noninvasive vagus nerve stimulation; PENS: percutaneous electrical nerve stimulation; PENS-Fp1Fp2: percutaneous electrical nerve stimulation over Fp1Fp2; RCT: randomized controlled trial; RR: rate ratio; rTMS: repetitive transcranial magnetic stimulation; Rt-nVNS: right vagus nerve stimulation; Sham/Control: Sham control or waiting list; single-hf-TMS-F3: single session high frequency rTMS over F3; SMD: standardized mean difference; sTMS: single-pulse TMS; sTMS-Oz: single-pulse TMS over Oz; STS: supraorbital transcutaneous stimulation; STS-Afz: supraorbital transcutaneous stimulator over Afz; SUCRA: surface under the cumulative ranking curve; taVNS: transcutaneous auricular vagus nerve stimulation; tDCS: transcranial direct current stimulation; TMS: transcranial magnetic stimulation; tONS: transcutaneous occipital nerve stimulation

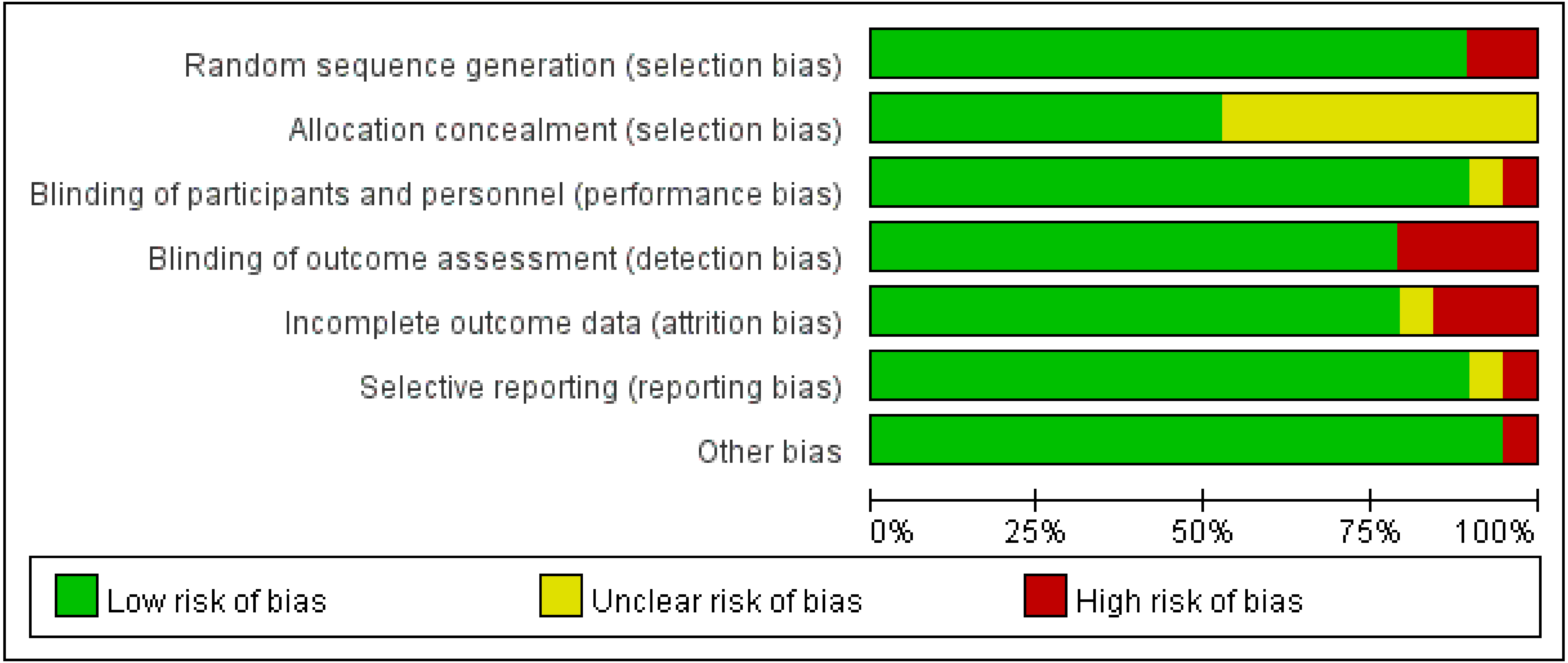

eFigure 4A overview of risk of bias

	Random sequence generation (selection bias)	Allocation concealment (selection bias)	Blinding of participants and personnel (performance bias)	Blinding of outcome assessment (detection bias)	Incomplete outcome data (attrition bias)	Selective reporting (reporting bias)	Other bias
Andrade, S.M. (2017)	−	+	+	+	−	+	+
Antal, A. (2011)	−	?	+	+	+	+	+
Auvichayapat, P. (2012)	+	?	+	+	+	+	+
Brighina, F. (2004)	+	?	+	+	+	+	+
Conforto, A.B. (2014)	+	+	+	+	+	−	−
Diener, H.C. (2019)	+	+	+	+	+	+	+
Kalita, J. (2016)	+	?	+	−	+	+	+
Kumar, A. (2021)	+	+	+	+	?	+	+
Li, H. (2017)	+	+	+	+	+	+	+
Lipton, R.B. (2010)	+	+	+	+	−	+	+
Liu, Y. (2017)	+	?	+	+	+	+	+
Misra, U.K. (2013)	+	?	+	+	+	+	+
Pohl, H. (2020)	+	+	?	+	+	?	+
Rahimi, M.D. (2020)	+	?	+	−	+	+	+
Rapinesi, C. (2016)	+	?	−	−	+	+	+
Schoenen, J. (2013)	+	+	+	+	+	+	+
Silberstein, S.D. (2016)	+	+	+	+	+	+	+
Tassorelli C (2018)	+	+	+	+	−	+	+
Zhang, Y. (2021)	+	?	+	−	+	+	+

eFigure 4B detailed risk of bias in each study

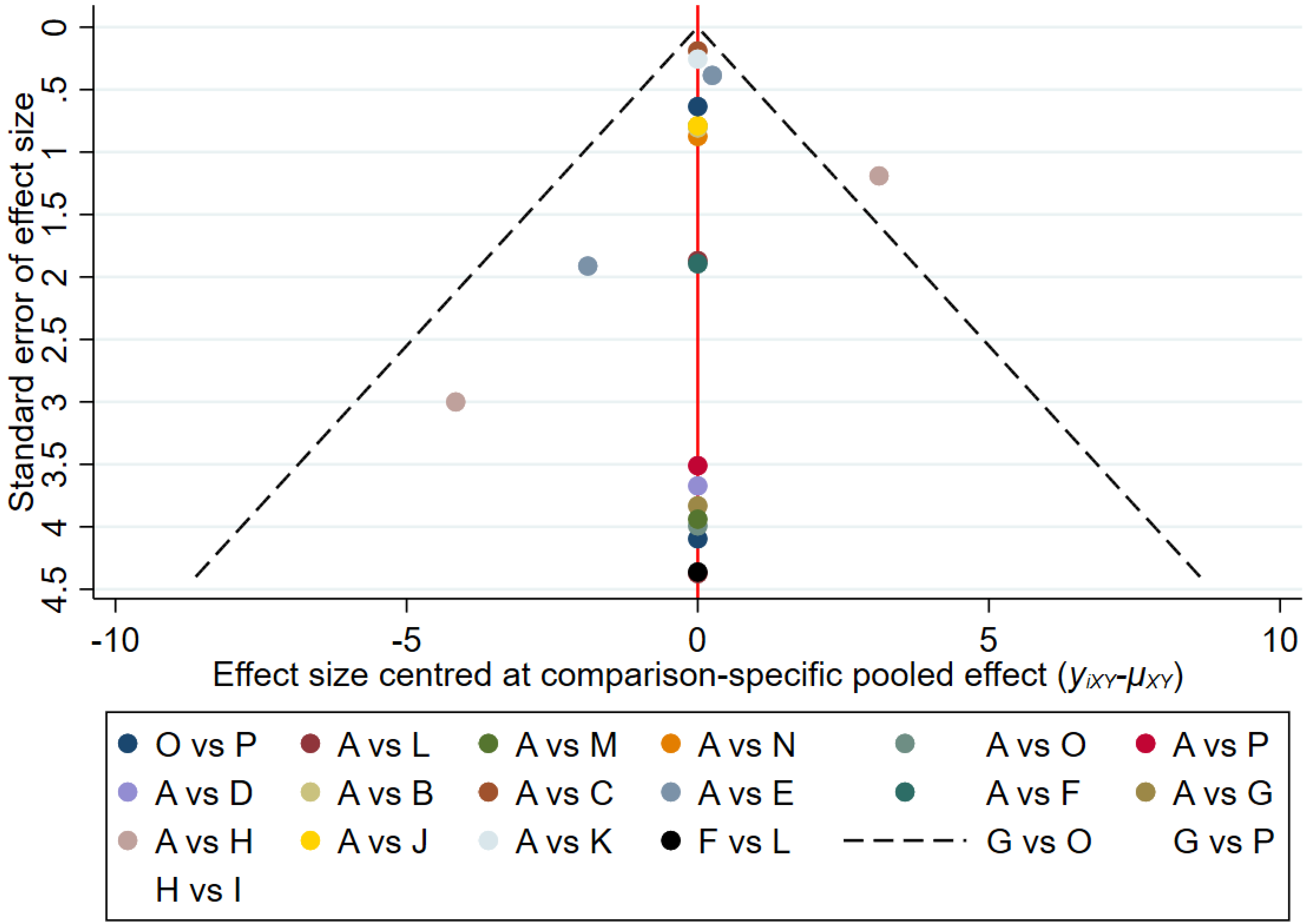

eFigure 5A Funnel plot of changes in monthly migraine days

Treatments used in eFigure 5A

A:	Sham/Control
B:	taVNS
C:	a-tDCS-Oz+c-tDCS-Cz
D:	c-tDCS-Oz+a-tCDS-Cz
E:	Rt-nVNS
F:	c-tDCS-CP4+a-tDCS-arm
G:	hf-tONS-Oz
H:	hf-TMS-F3
I:	single-hf-TMS-F3
J:	STS-Afz
K:	a-tDCS-C3+c-tDCS-Fp2
L:	c-tDCS-C4+a-tDCS-arm
M:	PENS-Fp1Fp2
N:	hf-TMS-C3
O:	af-tONS-Oz
P:	lf-tONS-Oz

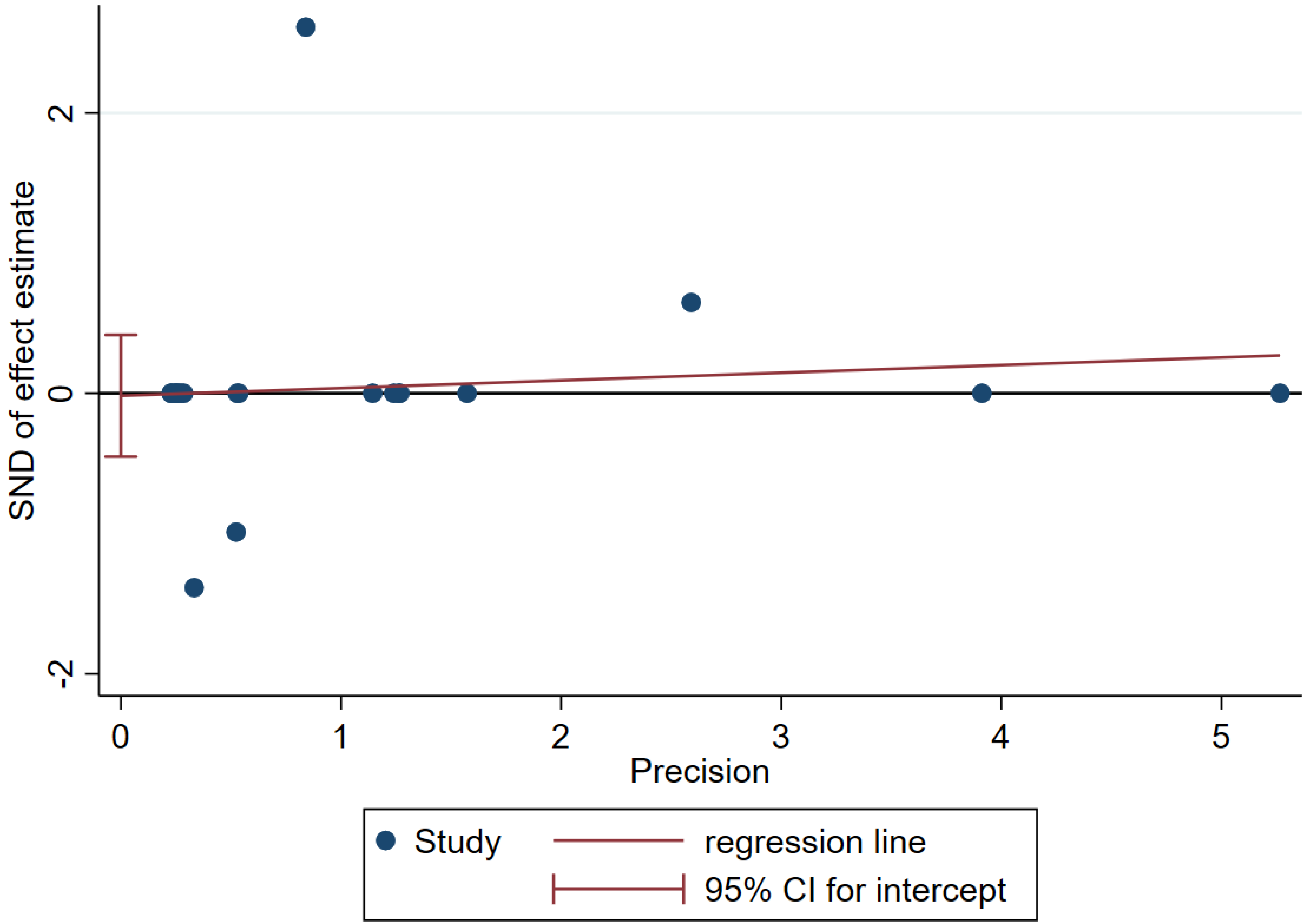

eFigure 5B Egger regression of NMA of changes in monthly migraine days

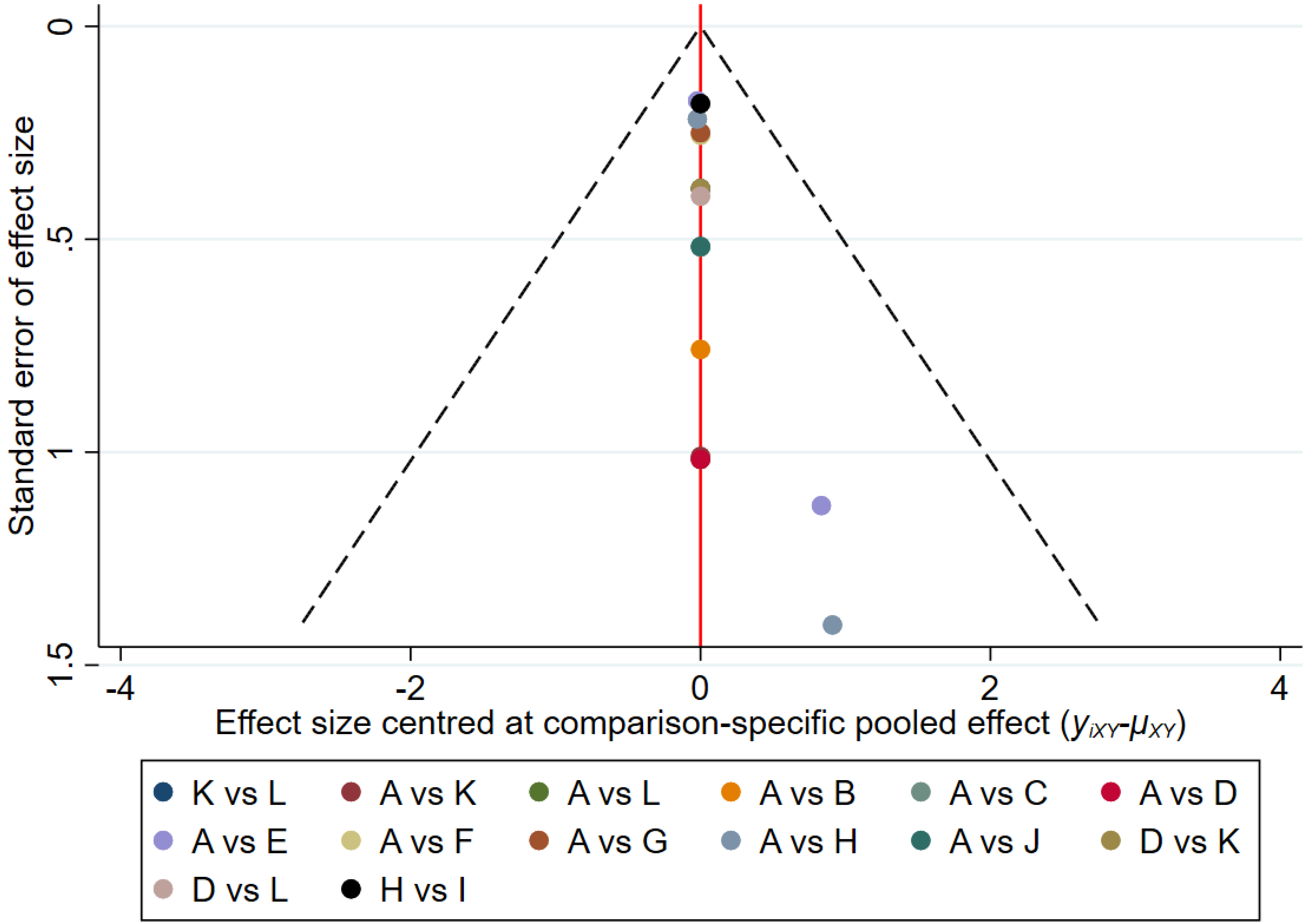

eFigure 5C Funnel plot of response rate

Treatments used in eFigure 5C

A: Sham/Control

B: a-tDCS-Oz+c-tDCS-Cz

C: PENS-Fp1Fp2

D: af-tONS-Oz

E: Rt-nVNS

F: Bi-nVNS

G: sTMS-Oz

H: hf-TMS-F3

I: single-hf-TMS-F3

J: STS-Afz

K: hf-tONS-Oz

L: lf-tONS-Oz

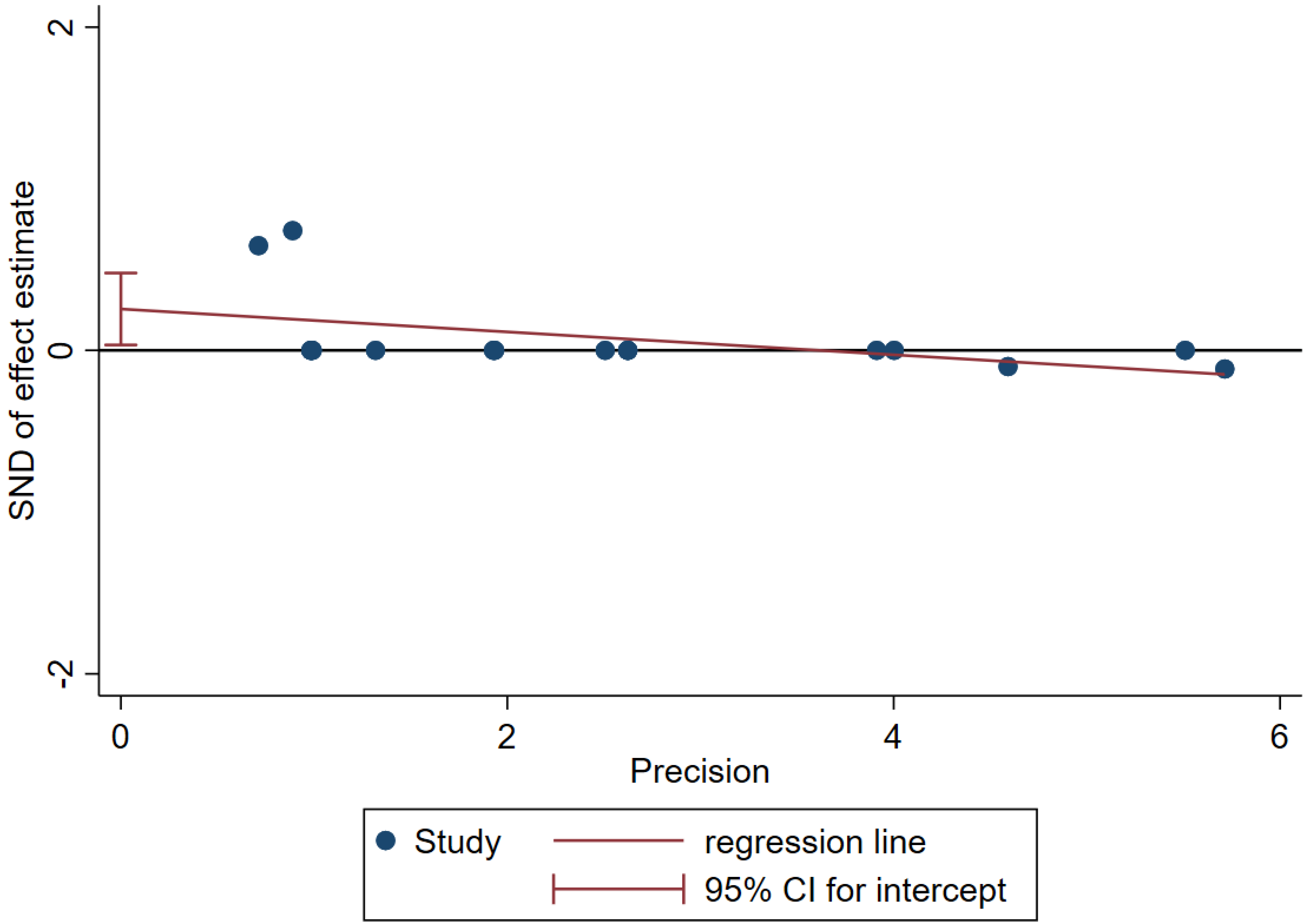

eFigure 5D Egger regression of NMA of response rate

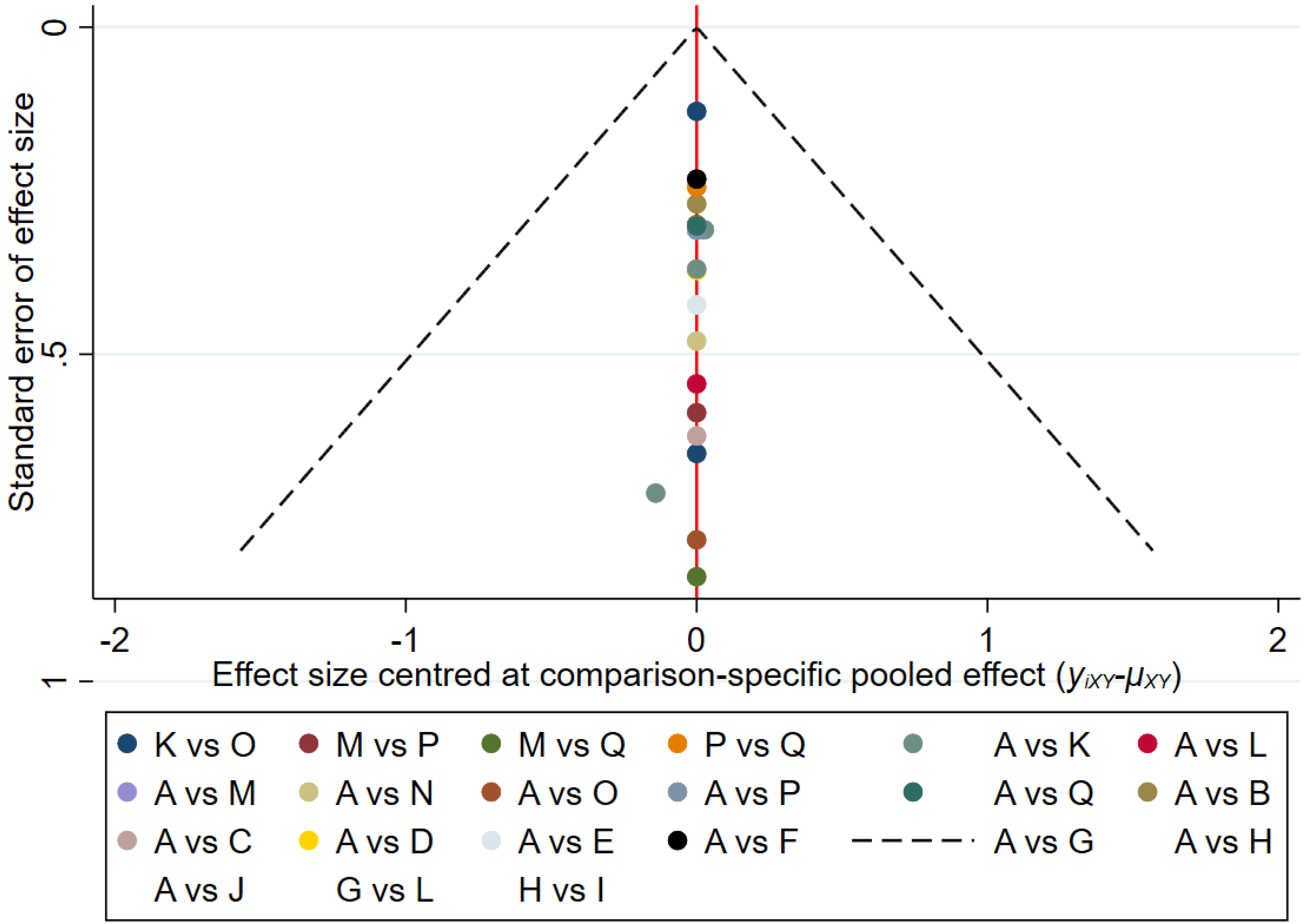

eFigure 5E Funnel plot of migraine pain severity

Treatments used in eFigure 5E

A: Sham/Control
B: taVNS
C: dTMS-F3
D: c-tDCS-Oz+a-tCDS-Cz
E: a-tDCS-Oz+c-tDCS-Cz
F: Bi-nVNS
G: c-tDCS-CP4+a-tDCS-arm
H: hf-TMS-F3
I: single-hf-TMS-F3
J: STS-Afz
K: a-tDCS-C3+c-tDCS-Fp2
L: c-tDCS-C4+a-tDCS-arm
M: af-tONS-Oz
N: hf-TMS-C3
O: a-tDCS-F3+c-tDCS-Fp2
P: lf-tONS-Oz
Q: hf-tONS-Oz

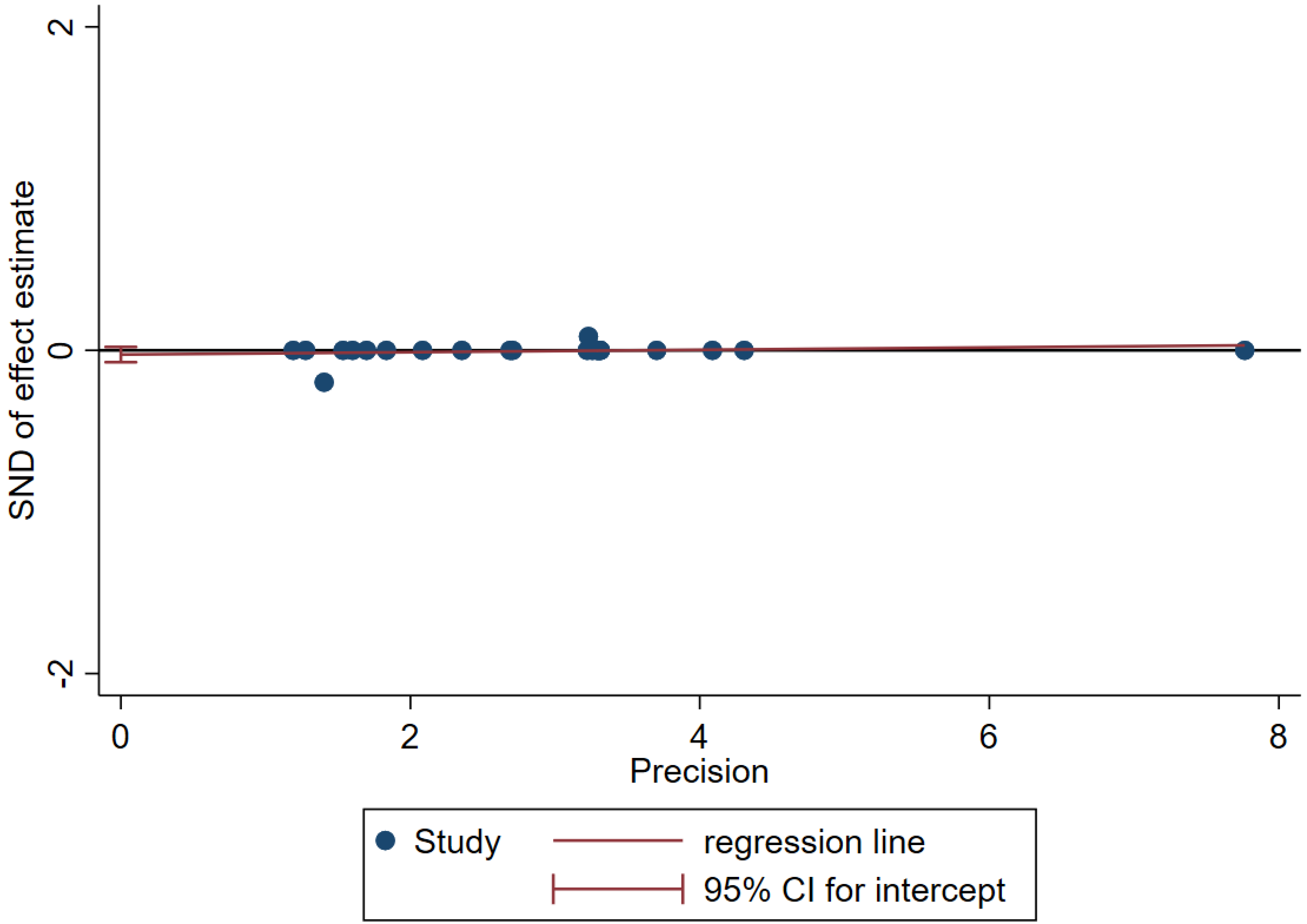

eFigure 5F Egger regression of NMA of migraine pain severity

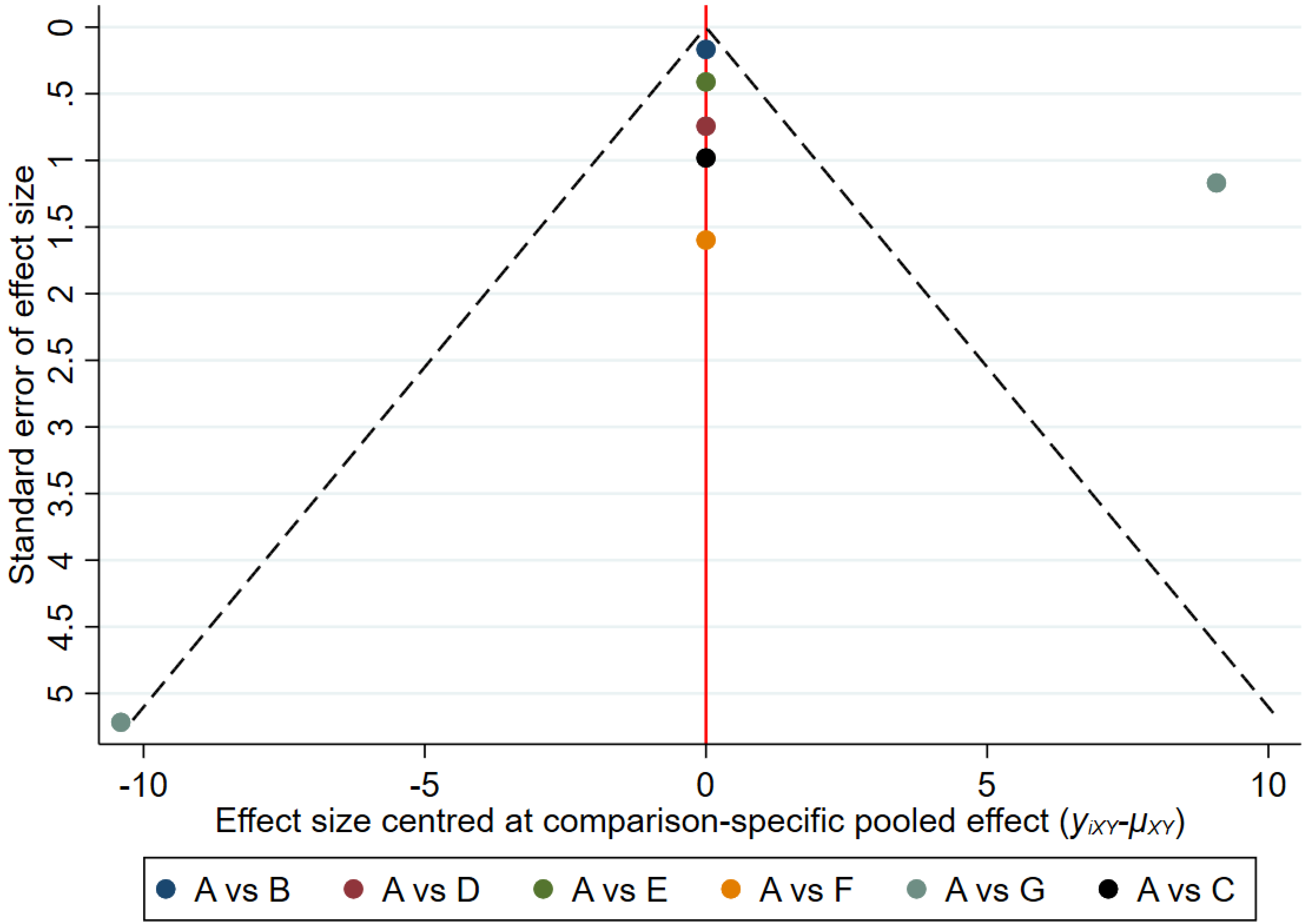

eFigure 5G Funnel plot of rescue medication

Treatments used in eFigure 5G

A: Sham/Control

B: a-tDCS-Oz+c-tDCS-Cz

C: dTMS-F3

D: PENS-Fp1Fp2

E: Rt-nVNS

F: STS-Afz

G: hf-TMS-F3

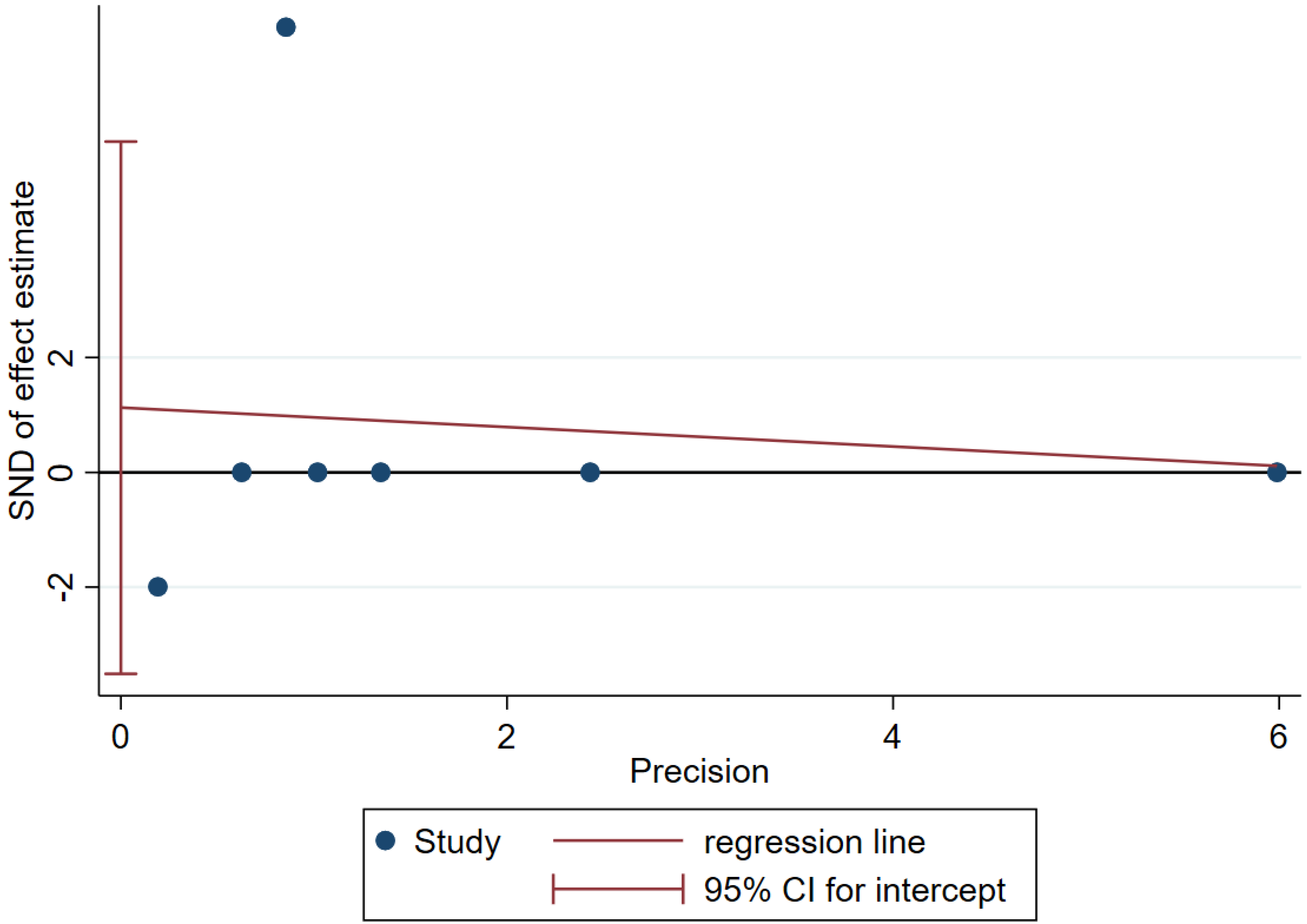

eFigure 5H Egger regression of NMA of rescue medication

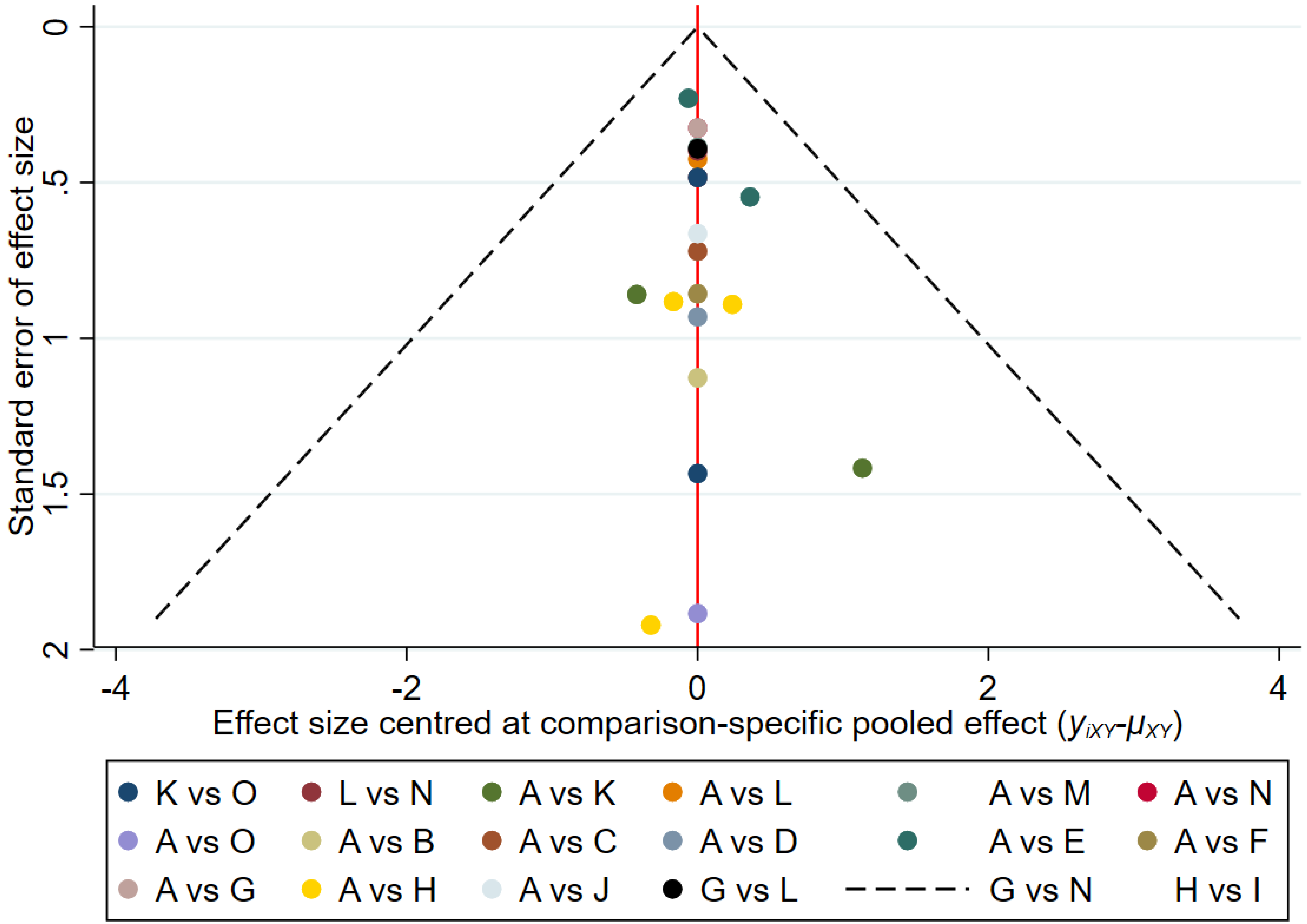

eFigure 5I Funnel plot of drop-out rate

Treatments used in eFigure 5I

A: Sham/Control
B: taVNS
C: PENS-Fp1Fp2
D: c-tDCS-Oz+a-tCDS-Cz
E: Rt-nVNS
F: Bi-nVNS
G: lf-tONS-Oz
H: hf-TMS-F3
I: single-hf-TMS-F3
J: STS-Afz
K: a-tDCS-C3+c-tDCS-Fp2
L: af-tONS-Oz
M: sTMS-Oz
N: hf-tONS-Oz
O: a-tDCS-F3+c-tDCS-Fp2

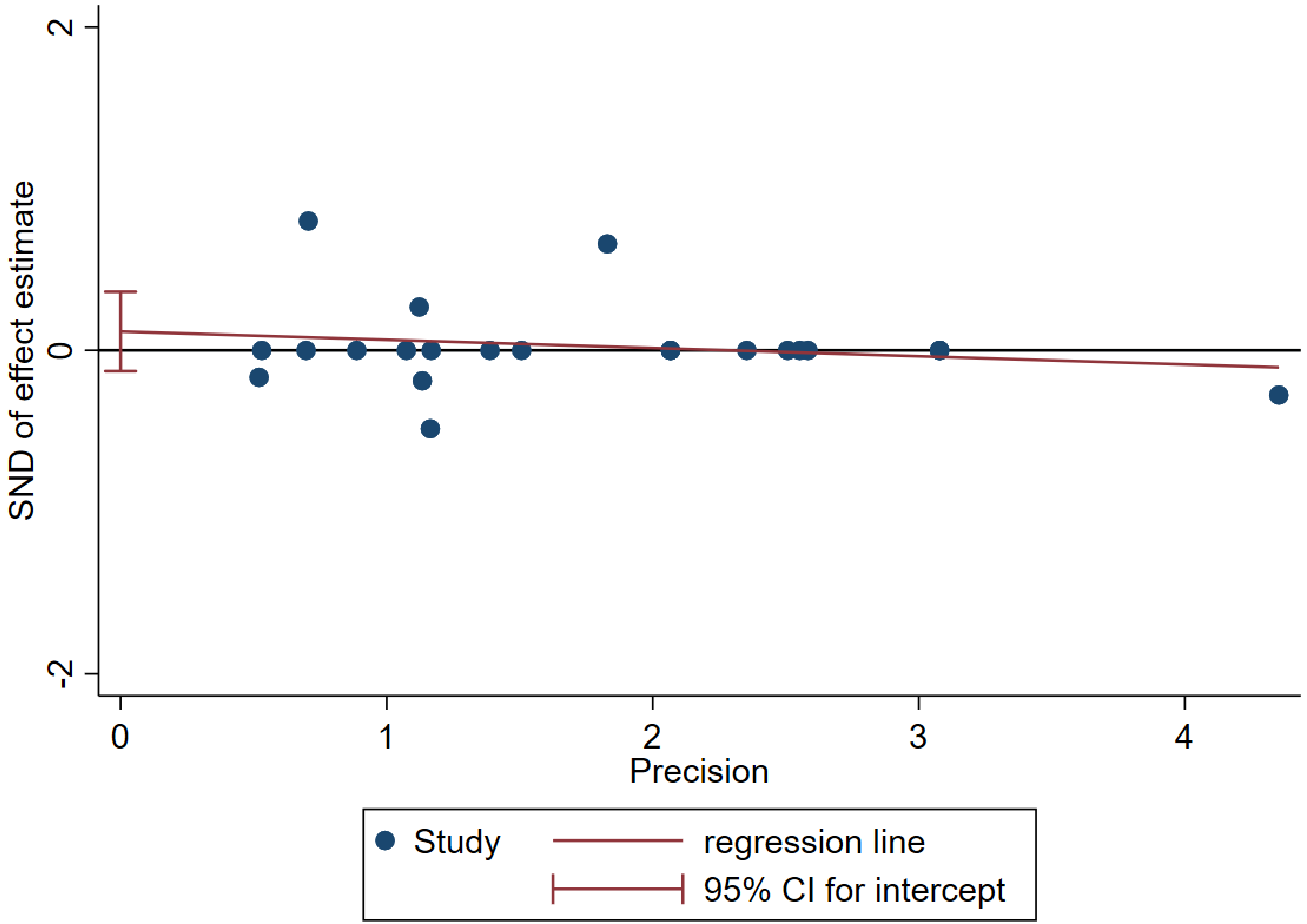

eFigure 5J Egger regression of NMA of drop-out rate

Figure legend of eFigure 5A-5J

Abbreviation: 95%CI: 95% confidence interval; af-tONS-Oz: alternating frequency tONS over Oz; a-tDCS-C3+c-tDCS-Fp2: anode tDCS over C3 + cathode tDCS over Fp2; a-tDCS-F3+c-tDCS-Fp2: anode tDCS over F3 + cathode tDCS over Fp2; a-tDCS-Oz+c-tDCS-Cz: anode tDCS over Oz + cathode over Cz; Bi-nVNS: bilateral vagus nerve stimulation; c-tDCS-C4+a-tDCS-arm: cathode tDCS over C4 + anode at left upper arm; c-tDCS-CP4+a-tDCS-arm: cathode tDCS over CP4 + anode at left upper arm; c-tDCS-Oz+a-tCDS-Cz: cathode tDCS over Oz + anode tCDS over Cz; dTMS-F3: deep TMS-F3; ES: effect size; hf-TMS-C3: high frequency rTMS over C3; hf-TMS-F3: high frequency rTMS over F3; hf-tONS-Oz: high frequency tONS over Oz; lf-tONS-Oz: low frequency tONS over Oz; MD: mean difference; NMA: network meta-analysis; nVNS: noninvasive vagus nerve stimulation; PENS: percutaneous electrical nerve stimulation; PENS-Fp1Fp2: percutaneous electrical nerve stimulation over Fp1Fp2; RCT: randomized controlled trial; RR: rate ratio; rTMS: repetitive transcranial magnetic stimulation; Rt-nVNS: right vagus nerve stimulation; Sham/Control: Sham control or waiting list; single-hf-TMS-F3: single session high frequency rTMS over F3; SMD: standardized mean difference; sTMS: single-pulse TMS; sTMS-Oz: single-pulse TMS over Oz; STS: supraorbital transcutaneous stimulation; STS-Afz: supraorbital transcutaneous stimulator over Afz; SUCRA: surface under the cumulative ranking curve; taVNS: transcutaneous auricular vagus nerve stimulation; tDCS: transcranial direct current stimulation; TMS: transcranial magnetic stimulation; tONS: transcutaneous occipital nerve stimulation
